# Reshaping Phosphatase Substrate Preference for Controlled Biosynthesis Using a “Design–Build–Test–Learn” Framework

**DOI:** 10.1002/advs.202309852

**Published:** 2024-03-19

**Authors:** Jiangong Lu, Xueqin Lv, Wenwen Yu, Jianing Zhang, Jianxing Lu, Yanfeng Liu, Jianghua Li, Guocheng Du, Jian Chen, Long Liu

**Affiliations:** ^1^ Key Laboratory of Carbohydrate Chemistry and Biotechnology Ministry of Education Jiangnan University Wuxi 214122 China; ^2^ Science Center for Future Foods Jiangnan University Wuxi 214122 China; ^3^ Shandong Runde Biotechnology co., LTD Taian 271200 China

**Keywords:** design–build–test–learn framework, *N*‐acetylglucosamine‐6‐phosphate, phosphatase, protein engineering, substrate preference

## Abstract

Biosynthesis is the application of enzymes in microbial cell factories and has emerged as a promising alternative to chemical synthesis. However, natural enzymes with limited catalytic performance often need to be engineered to meet specific needs through a time‐consuming trial‐and‐error process. This study presents a quantum mechanics (QM)‐incorporated design–build–test–learn (DBTL) framework to rationally design phosphatase BT4131, an enzyme with an ambiguous substrate spectrum involved in *N*‐acetylglucosamine (GlcNAc) biosynthesis. First, mutant M1 (L129Q) is designed using force field‐based methods, resulting in a 1.4‐fold increase in substrate preference (*k*_cat_/*K*_m_) toward GlcNAc‐6‐phosphate (GlcNAc6P). QM calculations indicate that the shift in substrate preference is caused by a 13.59 kcal mol^−1^ reduction in activation energy. Furthermore, an iterative computer‐aided design is conducted to stabilize the transition state. As a result, mutant M4 (I49Q/L129Q/G172L) with a 9.5‐fold increase in *k*_cat‐GlcNAc6P_/*K*_m‐GlcNAc6P_ and a 59% decrease in *k*_cat‐Glc6P_/*K*_m‐Glc6P_ is highly desirable compared to the wild type in the GlcNAc‐producing chassis. The GlcNAc titer increases to 217.3 g L^−1^ with a yield of 0.597 g (g glucose)^−1^ in a 50‐L bioreactor, representing the highest reported level. Collectively, this DBTL framework provides an easy yet fascinating approach to the rational design of enzymes for industrially viable biocatalysts.

## Graphical abstract

A design–build–test–learn (DBTL) framework for rational enzyme design is proposed to modify the substrate preference of the phosphatase BT4131. The best mutant, exhibiting significantly enhanced preference for *N*‐acetylglucosamine‐6‐phosphate (GlcNAc6P), is combined with a GlcNAc6P biosensor and applied to microbial cell factories, resulting in a substantial improvement in *N*‐acetylglucosamine biosynthesis.

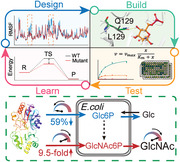

## 1 Introduction

Protein catalysts are the fundamental building blocks of microbial cell factories.^[^ ^1^ ^]^ In some contexts, bio‐based processes are limited by the insufficient performance of enzymes derived from natural evolution.^[^ ^2^ ^]^ Irrational strategy, also known as directed evolution, has achieved impressive accomplishments in protein engineering for production requirements.^[^ ^3^ ^]^ Although this approach allows obtaining positive mutations without prior knowledge of protein structure and catalytic mechanisms, the time‐consuming process of constructing and screening large‐scale mutant libraries poses significant challenges.^[^ ^4^ ^]^ As an alternative to directed evolution, the semirational strategy has significantly narrowed the scope of mutant libraries by identifying key positions for improving library quality based on advances in the resolution of protein 3D structures and catalytic mechanisms.^[^ ^5^ ^]^ For example, mannose biosynthesis has been improved by site‐saturation mutagenesis, which enhances the substrate preference for mannose‐6‐phosphate of the phosphatase from *Thermosipho atlanticus* (Ta‐PST).^[^ ^6^ ^]^ Although successful, the application of multisite iterative mutations is limited by insufficient accuracy.^[^ ^7^ ^]^

The design–build–test–learn (DBTL) framework is well known in synthetic biology and metabolic engineering.^[^ ^8^ ^]^ However, the occurrence and utility of the framework in protein engineering have only recently gained extensive attention.^[^ ^9^ ^]^ With the advent of computational enzyme design,^[^ ^10^ ^]^ the aided algorithms have provided new and deeper insights into the field of protein engineering.^[^ ^9^, ^11^ ^]^ For instance, recent advances in the application of quantum mechanics (QM) calculation based on density functional theory^[^ ^12^ ^]^ (DFT) in analyzing enzyme catalysis mechanisms have provided insights into the high‐energy‐barrier transition state conformation of various reactions.^[^ ^13^ ^]^ A new challenge for the field is to incorporate these aided algorithms directly into the DBTL framework for the iterative rational design of enzymes rather than trial and error.^[^ ^14^ ^]^

**Figure 1:**
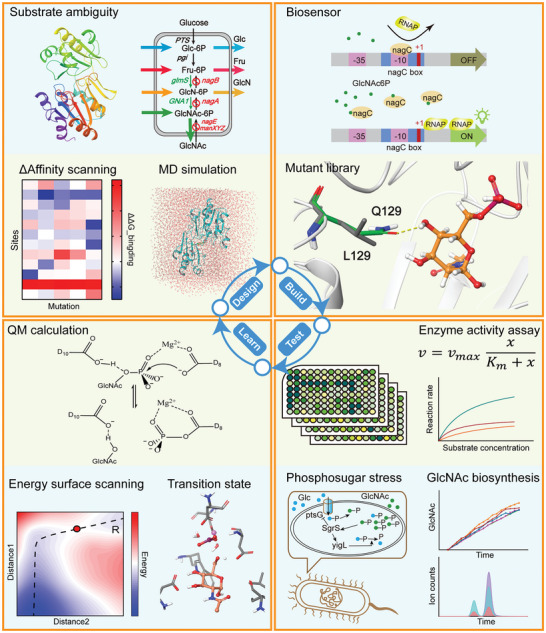
A DBTL framework for phosphatase substrate preference engineering. “Design”: GlcNAc synthesis pathway in *Escherichia coli*. Genes in green font represent overexpressed genes, whereas genes in red font represent knocked‐out genes. Phosphatases exhibit catalytic activity toward Glc6P, fructose‐6‐phosphate (Fru6P), glucosamine‐6‐phosphate (GlcN6P), and GlcNAc6P. The affinity changes of mutant variants with different substrates were predicted through in silico scans. Conformational changes when enzymes bind to different substrates were determined through molecular dynamics simulations. “Build”: Construction of the mutant and a GlcNAc6P‐responsive biosensor. “Test”: In vitro enzyme kinetic parameter assays and the application of enzymes in GlcNAc biosynthesis. “Learn”: Catalytic mechanism of phosphatases and potential energy surface scans to determine reaction pathways and high‐energy transition state conformations. Abbreviations are: PTS, phosphotransferase system; pgi, glucose‐6‐phosphate isomerase; glmS, glutamine–fructose‐6‐phosphate aminotransferase; GNA1, glucosamine‐6‐phosphate *N*‐acetyltransferase; nagA, GlcNAc6P deacetylase; nagB, GlcN6P deaminase; nagE, PTS system N‐acetylglucosamine‐specific EIICBA component; manXYZ, PTS system mannose‐specific EIIAB, EIIC and EIID components; nagC *N*‐acetylglucosamine repressor; RNAP, RNA polymerase; ptsG, PTS system glucose‐specific EIICB component; yigL, pyridoxal phosphatase.

This study presented a novel and efficient QM‐incorporated DBTL iterative framework to improve the performance of phosphatase BT4131 inside *N*‐acetylglucosamine (GlcNAc) cell factories (**Figure** **1**). Phosphatase BT4131 from *Bacteroides thetaiotaomicron* VPI‐5482 exhibited strong catalytic activity toward GlcNAc6P,^[^ ^15^ ^]^ but its stronger activity toward glucose‐6‐phosphate (Glc6P) could create a serious obstacle to efficient GlcNAc synthesis.^[^ ^16^ ^]^ This study started with a mine of the key binding sites responsible for substrate preference differences through molecular dynamics (MD) simulations, predicting the impact of mutations on affinity. Then, mutant L129Q with a 1.4‐fold increase in *k*_cat‐GlcNAc6P_/*K*_M‐GlcNAc6P_ and a 73.1% decrease in *k*_cat‐Glc6P_/*K*_m‐Glc6P_ was designed and tested, and the catalytic efficiency change that depends on activation energy was learned from QM simulation. Focusing on stabilizing high‐energy barrier conformation, an iterative binding energy prediction of mutation in the transition state was conducted. As a result, mutant M4 with the highest improvement in catalytic efficiency was obtained, with a 9.5‐fold increase in *k*_cat‐GlcNAc6P_/*K*_M‐GlcNAc6P_ and a 59% decrease in *k*_cat‐Glc6P_/*K*_m‐Glc6P_ compared to wild type (WT). Ultimately, mutant M4 in the GlcNAc‐producing chassis was heterologously overexpressed, resulting in the highest reported GlcNAc titer (217.3 g L^−1^) with a 25.8% increase and a yield of 0.597 g (g glucose)^−1^ in a 50‐L bioreactor. This iterative framework in rational protein engineering represents significant progress and offers a valuable tool for designing other industrially relevant biocatalysts.

## 2 Results and Discussion

### 2.1 Redesign of the Substrate‐Binding Pocket of BT4131

**Figure 2:**
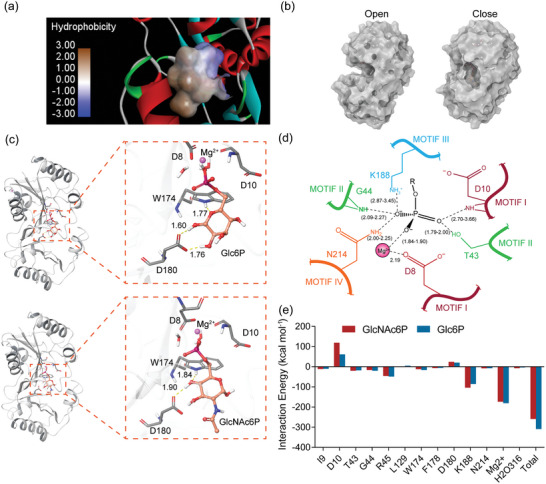
3D structural analysis and molecular docking results. a) Hydrophobicity distribution of the substrate‐binding pocket in BT4131. b) Conformational changes between “open” and “close” states during molecular dynamics simulations. c) Docking results of BT4131 with Glc6P and GlcNAc6P, with yellow dashed lines representing hydrogen bonds in the figure. Only portions of substrate molecules, excluding the phosphate groups, forming hydrogen bonds with the enzyme are labeled. d) Interaction and associated distances between the phosphate group of the substrate molecule and the core structural domain of the enzyme. e) Calculation of the contribution of each interacting residue to the binding energy.

BT4131 consists of two domains, with the larger domain being the “conserved core” domain that contains the active site. The coordination between Mg^2+^ and residues, such as D8 and D10, forms a stable catalytic active center.^[^ ^17^ ^]^ An evident characteristic of the substrate‐binding pocket in BT4131 is that most residues surrounding the ligand are hydrophobic (**Figure** **2**a). Given that BT4131 exhibits greater catalytic activity toward Glc6P with a lesser volume than GlcNAc6P, this study first validated whether steric hindrance is an important factor influencing substrate preference. This validation was achieved through a series of calculations and simulations related to the substrate pocket volume. The two domains of BT4131 resembled “pockets” and “caps,” presenting an “open” conformation without substrate during MD simulation (Figure 2b). As a result, the substrate‐binding pocket of BT4131 had a volume of 138.9 Å^3^ and increased to >300 Å^3^, indicating that adjusting the binding pocket volume does not effectively modulate the catalytic activity. Therefore, this study attempted to regulate substrate preference toward GlcNAc6P by redesigning the polarity distribution of residues comprising the binding pocket.

**Figure 3:**
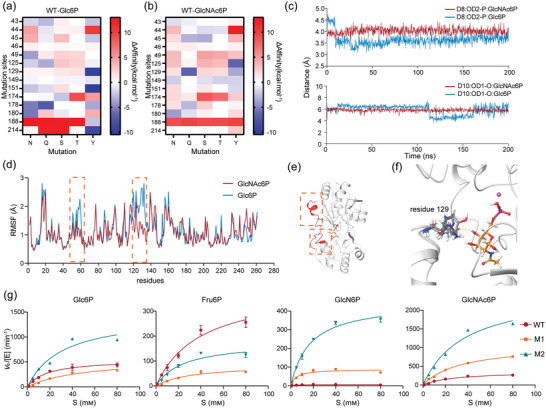
Molecular dynamics (MD) simulations and virtual mutation‐guided enzyme engineering modifications. a) Prediction of affinity changes resulting from the mutation of residue within a 3 Å radius of the substrate to each uncharged polar residue in WT‐Glc6P. b) Prediction of affinity changes resulting from the mutation of residue within a 3 Å radius of the substrate to each uncharged polar residue in WT‐GlcNAc6P. c) Distances between key residues (D8 and D10) and substrate molecules during MD simulations. d) RMSF during MD simulations of BT4131‐Glc6P and BT4131‐GlcNAc6P complexes. Regions within orange dashed lines represent parts where the RMSF values of the two complexes differ significantly. e) Display of the regions with significantly different RMSF values in the 3D structure of the complexes. The relevant residues are highlighted in orange. f) Comparison of the conformations of residue 129 mutants, where the L129Q mutant forms a hydrogen bond with GlcNAc6P. g) In vitro assays of catalytic activity on four phosphosugar substrates by WT, M1, and M2. Data presented as mean values ± SD from three independent biological replicates (*n* = 3).

To obtain binding conformations and analyze interactions between the enzyme and substrates, flexible molecular docking was performed (Figure 2c,d). In the binding pocket of BT4131‐GlcNAc6P and BT4131‐Glc6P complexes, 14 residues (T43, G44, R45, P46, I49, F125, L129, Q149, T151, W174, F178, D180, K188, and N124) within a 3 Å radius of substrate molecules were located and selected for further analysis. To reduce time‐consuming work, an efficient in silico assessment was conducted to identify mutants that could potentially enhance binding with GlcNAc6P while weakening the affinity for Glc6P. Specifically, the assessment process included the initial modeling of mutants, conformational adjustments to the main and side chains of residues, and the calculation of differences in affinity compared to the initial conformation. Owing to the strong polarity of the substrate molecule, the range of the initial assessment of mutation was specified for each type of uncharged polar residue, and the calculated results of Δaffinity_Glc6P_ and Δaffinity_GlcNAc6P_ are shown in **Figure** **3**a,b. In addition, MD simulations of the BT4131‐GlcNAc6P and BT4131‐Glc6P complexes were performed at 310.15 K for 200 ns to further narrow the test range. Glc6P exhibited more conformational instability during the simulation process, consistently adjusting to a conformation closer to the key active residues that appear to contribute to the high catalytic activity of BT4131 toward Glc6P (Figure 3c). Meanwhile, significant differences were observed in root mean square fluctuation (RMSF) values during the simulation process between the two complexes in the ranges of residues 50–60 and 120–135 (Figure 3d,e), and mutant L129Q exhibited positive Δaffinity_Glc6P_ and negative Δaffinity_GlcNAc6P_, especially the lowest ΔΔaffinity (Δaffinity_GlcNAc6P_‐Δaffinity_Glc6P_) among all mutations (−7.31 kcal mol^−1^). The oxygen atom (OE) of glutamine as the acceptor formed a new hydrogen bond in the complex conformation of L129Q (Figure 3f). To investigate whether the mutation site could affect the structural stability of proteins, the stability of mutants was predicted. The Δstability value for L129Q was −14.05 kcal mol^−1^, contributing to improved stability (Figure S1a, Supporting Information). Based on the above results, mutant L129Q was redesigned by site‐directed mutagenesis, generating mutant M1 for in vitro enzyme activity assays (Figure 3g). Results showed that the *k*_cat‐GlcNAc6P_/*K*_m‐GlcNAc6P_ value of M1 reached 31.88 mm ^−1^ min^−1^, exhibiting a 1.4‐fold increase compared to WT, whereas the *k*_cat‐Glc6P_/*K*_m‐Glc6P_ value decreased by 63.5% to 11.32 mm ^−1^ min^−1^. Besides, *k*_cat‐Fru6P_/*K*_m‐Fru6P_ decreased to 2.71 mm ^−1^ min^−1^, representing a 73.1% decrease (**Table** **1**). This positive correlation in activity toward Fru6P and Glc6P may have occurred due to structural similarity. Overall, redesigning polarity distribution effectively altered substrate preference without steric hindrance inside the binding pocket and tunnel.

**Table 1:** *k*_cat_/*K*_m_ values of Wild type and various mutants for different substrates.^a)^

*k*_cat_/*K*_m_ (mm ^−1^ min^−1^)	Wild type	M1 (L129Q)	M2 (L129Q/G172L)	M3 (I49Q/L129Q)	M4 (I49Q/L129Q/G172L)
Glc6P	30.98 ± 1.75	11.32 ± 2.28	46.38 ± 7.36	12.2 ± 3.96	12.58 ± 2.25
Fru6P	10.08 ± 1.67	2.71 ± 0.98	7.25 ± 1.41	1.47 ± 0.34	—^b)^
GlcN6P	2.08 ± 0.48	19 ± 3.7	26.39 ± 1.49	2.09 ± 0.48	—^b)^
GlcNAc6P	13.28 ± 1.83	31.88 ± 1.78	67.48 ± 4.95	74.72 ± 5.64	126.27 ± 7.84

^a)^ Kinetic parameters were determined in an 80‐µL reaction system containing 50 mm HEPES, 5 mm MgCl_2_, 0.1–1 µm purified enzyme, and 1–80 mm substrate. The reaction was performed at 37 °C with shaking at 750 rpm for 10 min. See the Experimental Section for experimental details;

^b)^ “—” means not tested.

### 2.2 Enhancing Affinity through Remote Mutation‐Induced Allosteric Communication

To further improve the substrate preference of M1 for GlcNAc6P, a new round of mutation scanning in silico was conducted using M1‐GlcNAc6P and M1‐Glc6P complexes. The range of this scanning round encompassed two residues upstream of the substrate pocket, aiming to enhance the binding between M1 and GlcNAc6P via downstream conformational changes induced by mutations in upstream residues. Most mutations of residues 42 and 172 led to a decrease in affinity with Glc6P and an increase in affinity with GlcNAc6P (Figure S1, Supporting Information). However, residue 42 induced significant fluctuations in key active residues owing to its proximity to the catalytic core. The instability of the coordination structure comprising D8 and D10 residues with Mg^2+^ has the potential to affect catalytic activity, indicating that increasing affinity alone may not improve the progression of the reaction. Considering the above results, G172N, G172Y, and G172L mutations were selected for construction and initial screening. M1/G172L (M2) exhibited a 4.1‐fold increase (67.48 mm ^−1^ min^−1^) in the *k*_cat‐GlcNAc6P_/*K*_m‐GlcNAc6P_ value compared to WT, highlighting the advantages of catalytic activity (Figure 3g). Unexpectedly, its selectivity for GlcNAc6P did not improve and the *k*_cat‐Glc6P_/*K*_m‐Glc6P_ of M2 increased by 3.1‐fold and 49.7% compared to M1 and WT, respectively, proving that the transmission of conformational changes induced by residue mutations at remote sites can also affect the active center and change the catalytic activity. In addition, a more accurate method combined with an understanding of the catalytic mechanism should be reconstructed to accurately analyze the factor of improvement in catalytic activity for rational enzyme design.

### 2.3 Revealing the Mechanism of Substrate Preference Shift Based on QM Simulation

“Learn” is a crucial segment in the DBTL framework, which demands a mechanistic analysis of the obtained results to efficiently guide the iterative design cycle. QM calculations based on DFT have been employed to elucidate the principles underlying the catalytic activity changes of the mutant.^[^ ^13b^ ^]^ First, mutant M1 with a significant change in substrate selectivity was selected as a target for comparison with the WT. According to the catalytic mechanism of haloalkanoic acid dehalogenase superfamily phosphatases, the dephosphorylation reaction typically consists of two steps: one involving the phosphate group transfer under the nucleophilic attack of D8 and another involving the hydrolytic cleavage of the phosphate group of phosphorylated D8.^[^ ^18^ ^]^ Calculations were conducted solely for the first step of the reaction owing to the identical hydrolysis process of the phosphate group during the catalytic reaction of different substrates.

**Figure 4:**
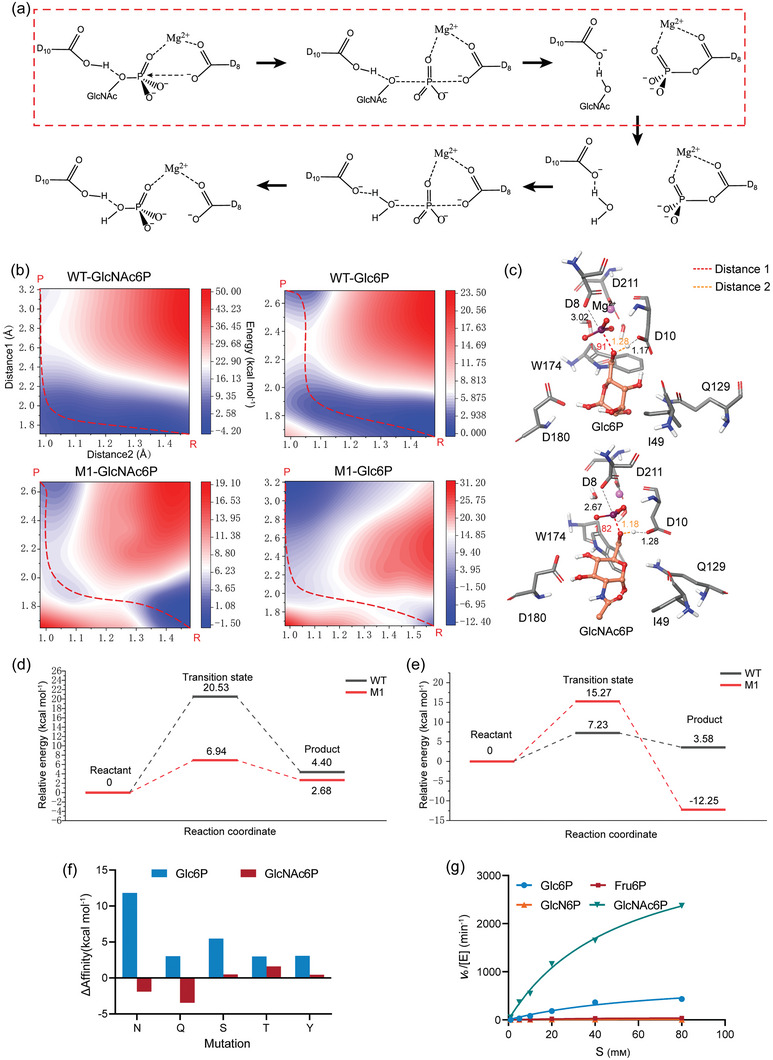
Quantum mechanical calculation results of the catalytic reaction process and the construction of a novel mutant. a) Presentation of the catalytic mechanism of BT4131. b) Potential energy surface scans of the phosphate group transfer reaction in four complexes (WT‐Glc6P, WT‐GlcNAc6P, M1‐Glc6P, and M1‐GlcNAc6P). Distance1 represents the distance between the negatively charged oxygen atom (OD2) of the nucleophilic reagent D8 and the phosphorus atom of the phosphate group. Distance2 represents the distance between the oxygen atom (OF) forming the phosphodiester bond in the substrate molecule and the H^+^ provided by D10. The red dashed line in the figure represents the lowest‐energy reaction path. “P” represents the product, and “R” represents the reactant. c) Transition state conformation of M1‐Glc6P and M1‐GlcNAc6P. d) Energy profile diagram for GlcNAc6P, illustrating the progression from reactants to transition states to products, with the reactant energy set as the zero point. e) Energy profile diagram for Glc6P, depicting the energy changes from reactants to transition states to products, with the reactant energy set as the zero point. f) Prediction of affinity changes resulting from the mutation of residue 49 to each uncharged polar residue in the transition state conformation of M1‐GlcNAc6P and M1‐Glc6P. g) In vitro catalytic activity assay for M3. Data presented as mean values ± SD from three independent biological replicates (*n* = 3).

WT‐GlcNAc6P, WT‐Glc6P, M1‐GlcNAc6P, and M1‐Glc6P complexes underwent potential energy surface scanning to undergo energy changes throughout the phosphate group transfer process and the specific reaction pathways. The distribution of various protonation states of GlcNAc6P and Glc6P at different pH values was analyzed by calculating pKa values. The conformation with two negative charges has the highest proportion at pH 7, followed by the conformation with one negative charge (Figure S2, Supporting Information). The conformation with two negative charges was selected for subsequent QM calculations. In **Figure** **4**a, a 2D energy scan was more suitable for elucidating the reaction process due to the involvement of two variables in this step (the transfer of H^+^ from D10 to the oxygen atom [OF] of GlcNAc and the nucleophilic attack of the phosphate group by D8). In addition, the lowest‐energy reaction pathway was identified, and the conformation at the highest energy point in the pathway was the transition state (Figure 4b). Results revealed that the position of the transition state and the reaction pathways change with residue mutations and that the high‐energy transition state of M1 is closer to the reactant than that of WT. Meanwhile, in the reactant conformation, the phosphorus atom of GlcNAc6P approached the nucleophilic reagent D8 by 0.45 Å under the influence of residue Q129, whereas that of Glc6P moved away by 0.15 Å (all obtained reactant, transition state, and product conformations are shown in Figure S3, Supporting Information). As expected, the above results are also consistent with MD simulation results. Specifically, the distance between Glc6P and D10 became unstable, leading to an obstacle for forming a stable reactive state, whereas the OF of GlcNAc6P can maintain a closer and more stable distance to D10 (Figure S4b, Supporting Information). Furthermore, the sharp decrease in root mean square deviation (RMSD) value for the Glc6P molecule indicated that Glc6P is constrained in an unfavorable conformation, hindering the progression of the reaction (Figure S4c, Supporting Information). Besides, in the transition state structure of M1‐GlcNAc6P, a “polar triangle” formed by D180, W174, and Q129 stabilized the substrate by forming a hydrogen bond (Figure 4c). The obvious conformational change of Glc6P between the transition state and reactant structures was unfavorable for binding the phosphate group to the catalytic core, resulting in increased energy and decreased stability. Subsequently, taking the reactant energy as the zero point, the relative energy changes of the transition state and products for each complex are shown in Figure 4d,e. Compared to WT, there was a 13.59 kcal mol^−1^ decrease in the energy barrier for M1‐GlcNAc6P and an 8.04 kcal mol^−1^ increase in the energy barrier for M1‐Glc6P. In addition, a 15.83 kcal mol^−1^ decrease in the energy of the product structure was observed due to the increased distance to D8 and the enhancement in binding affinity between the glucose molecule and M1 after the detachment of the phosphate group. In summary, the change in selectivity of M1 is attributed to variations in reaction energy barriers.

### 2.4 Stabilizing Transition State Conformation for Iterative Improvement Catalytic Efficiency

To obtain mutants with optimal catalytic efficiency and substrate preference, this study conducted an iterative design of mutations and focused on optimizing the affinity of the transition state structure for the regulation of the reaction activation energy. The hydrophobic residue I49 near the acetyl group has the potential to mutate to a polar residue for forming hydrogen bonds, and it was hypothesized that it is possible to stabilize the transition state structure of GlcNAc6P rather than Glc6P. To this end, mutant affinity predictions were conducted on the transition state structures of M1‐GlcNAc6P and M1‐Glc6P, with the range specified as I49 mutating to five uncharged polar residues. In Figure 4f, mutant I49Q exhibited the highest preference toward GlcNAc6P and a 3.02 kcal mol^−1^ Δaffinity_Glc6P_ in prediction results. Then, the *k*_cat‐GlcNAc6P_/*K*_m‐GlcNAc6P_ of M3 (M1/I49Q) increased by 4.6‐fold to 74.72 mm ^−1^ min^−1^, accompanied by a sharp decrease (60.6%) of *k*_cat‐Glc6P_/*K*_m‐Glc6P_ compared to WT, which showed that Q49 and Q129 have a significant inhibitory effect on Glc6P activity. Based on the above results, all possible combinations were tested and a three‐point mutation I49Q/L129Q/G172L named M4 was built. The catalytic efficiency of mutant M4 significantly increased, with a 9.5‐fold increase in *k*_cat‐GlcNAc6P_/*K*_m‐GlcNAc6P_ (126.27 mm ^−1^ min^−1^) and a 59% decrease in *k*_cat‐Glc6P_/*K*_m‐Glc6P_ (12.58 mm ^−1^ min^−1^) compared to WT, respectively.

The excellent performance of the iterative mutant M4, along with the library containing <10 variants in this study, demonstrated that this approach could effectively reduce the time and cost of mining the enzyme potential and obtain the high‐performance iterative mutation with few trials. Taken together, QM simulation provided more precise structures and analyzed the reaction process from an energy perspective, obtaining efficient iterative mutants under conditions of a short‐term, small‐scale mutant library. Moreover, by correlating catalytic activity data with the new mutant's high‐energy barrier conformation obtained through QM simulation, each iteration became more efficient, contributing to a deeper understanding of the mechanism.

### 2.5 Application of Phosphatase Mutants in GlcNAc Biosynthesis

**Figure 5:**
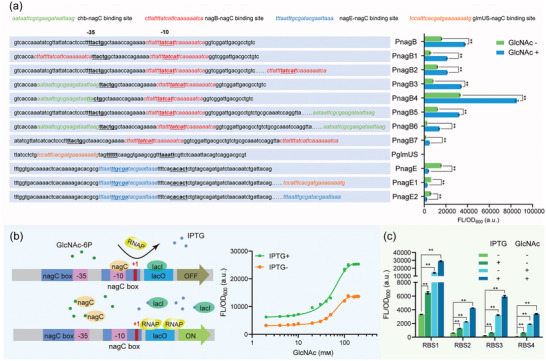
Construction of GlcNAc6P‐responsive biosensors and screening of phosphatases from different sources. a) By inserting different nagC‐binding sequences into various regions of the promoter, the inhibitory effect of nagC is enhanced, optimizing its response range. The response range is indicated by fluorescence intensity. b) By inserting a lactose operon, it is regulated by GlcNAc6P and isopropyl‐beta‐D‐thiogalactopyranoside (IPTG). Cultures with different GlcNAc concentrations are grown with and without IPTG, and their dynamic response ranges are tested. c) Further optimization of the response range is achieved by replacing the RBS sequence. Data presented as mean values ± SD from three independent biological replicates (*n* = 3). Two‐tailed‐Student's *t*‐test evaluated significance, ^*^ *P* < 0.05, ^**^ *P* < 0.01.

Encouraged by the successful engineering of phosphatase BT4131 in vitro using the QM‐incorporated DBTL framework, this study sought to evaluate its universality in microbial cell factories. First, a genetic circuit based on the GlcNAc6P‐responsive transcription factor nagC was designed to dynamically regulate the expression intensity of phosphatase to maximize the performance of mutant M4.^[^ ^19^ ^]^ Specifically, the intracellular GlcNAc6P concentration was coupled with the expression intensity of phosphatase to achieve efficient GlcNAc6P dephosphorylation while minimizing its influence on the intermediate and normal metabolism of various phosphates. Various nagC‐binding sites were inserted into regions such as −35, −10, and +1 of PnagB and PnagE promoters, resulting in nine hybrid promoters with different dynamic response ranges. WT promoters PnagB, PnagE, and PglmUS and the aforementioned hybrid promoters were placed upstream of sfGFP, and their response ranges were indicated by fluorescence intensity. The response range of promoter PnagB3 with the chb–nagC‐binding site inserted upstream of the −35 region was 8130–34039, indicating an intensity of activation close to WT nagB and a larger response range (**Figure** **5**a). In contrast, PnagE exhibited the opposite behavior, decreasing fluorescence intensity with the addition of extracellular GlcNAc. Considering all factors, PnagB3 was chosen for further study. To further reduce the impact of dephosphorylation on early cell growth, a lactose operon (lacO) site was inserted downstream of PnagB3, resulting in the PnagB3‐lacO promoter, which is under dual regulation by GlcNAc6P and IPTG (Figure 5b). By replacing four different ribosome binding site (RBS) sequences, more biosensors with different response ranges were obtained (Figure 5c).

**Figure 6:**
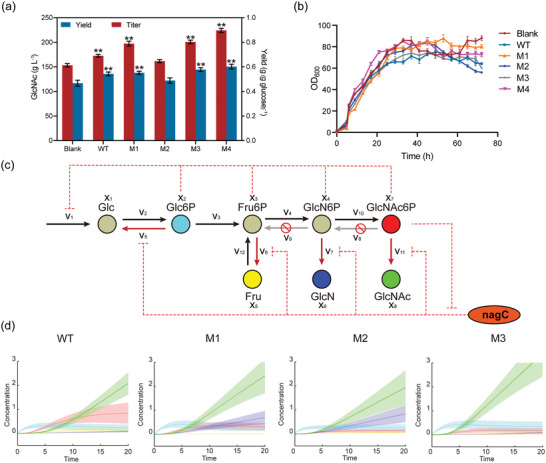
Application of phosphatase mutants in GlcNAc biosynthesis and analysis of the kinetic model. a) The GlcNAc titer and yield in the 50‐L bioreactor fermentation validation of the GlcNAc‐producing strain after integrating phosphatases. b) Growth curves for the aforementioned strains in the fermentation validation. c) Schematic representation of the kinetic model for the GlcNAc synthesis process based on the Michaelis–Menten equation. d) Model simulation results, with different colored lines representing the substances consistent with (c). Data presented as mean values ± SD from three independent biological replicates (*n* = 3). One‐way ANOVA followed by post‐hoc Dunnett's test evaluated significance, ^*^ *P* < 0.05, ^**^ *P* < 0.01.

Subsequently, each positive mutant was integrated into the genome of GlcNAc‐producing strains under the dynamic control of the GlcNAc6P biosensor PnagB3‐lacO. Production testing results showed that GlcNAc titers for strains integrating M1, M3, and M4 increased to 197.3, 200.8, and 217.3 g L^−1^, representing percentage increases of 14.2%, 16.3%, and 25.8% compared to WT, respectively (**Figure** **6**a). Besides, the yield of the three above strains increased respectively from 0.543 to 0.552, 0.548, and 0.597 g (g glucose)^−1^. Conversely, due to a general increase in three types of precursor activity, M2 decreased the GlcNAc titer to 161.6 g L^−1^, representing a 6.4% reduction compared to WT. The growth curve (OD_600 nm_) of the fermentation process (Figure 6b) demonstrated that the secondary growth phenomenon induced by phosphosugar pressure^[^ ^20^ ^]^ in production strains is generally alleviated in strains integrating phosphatase. The intracellular phosphosugar concentration was measured to verify this hypothesis, and the intracellular GlcNAc6P concentration decreased by 65.4% (12.7 µm gDCW^−1^) compared to the chassis strain (Figure S5a, Supporting Information). The high catalytic activity of GNA1^[^ ^21^ ^]^ in GlcNAc‐producing strains led to a low intracellular GlcN6P concentration. Moreover, the intracellular Glc6P concentration increased fourfold in the early fermentation stages, enhancing the strain's metabolic activity. Phosphatase addition did not reduce the intracellular Glc6P concentration but led to an increase. It is inferred that the reason was the relief of phosphosugar stress,^[^ ^20^, ^22^ ^]^ resulting in an accelerated glucose uptake rate by cells.^[^ ^23^ ^]^ In previous studies, although product synthesis and cell growth were balanced by utilizing mixed carbon sources, it did not address the inhibition of glucose uptake rate caused by intracellular GlcNAc6P accumulation.^[^ ^24^ ^]^ This significantly limited the efficiency of GlcNAc synthesis.

To demonstrate that the advantages of the mutant observed in vitro are also exhibited in living cells, a dynamic model was constructed. This model integrated the GlcNAc synthesis pathway, characterized by Michaelis–Menten equations, with the dynamic feedback system of nagC, represented by Hill kinetics equations, to simulate the GlcNAc synthesis process (Figure 6c). Phosphate pressure is regulated through negative feedback on glucose transport velocity by four different phosphosugar concentrations. The dynamic response of the biosensor is achieved by negative feedback regulation, with GlcNAc6P concentration influencing the rates of four different phosphosugar dephosphorylation reactions. In the simulation results of M2, Glc6P was repressed to extremely low concentrations, disrupting the reaction rate and leading to a decrease in GlcNAc concentration compared to M1. In the simulation results of M3, due to a significant preference for GlcNAc6P, the GlcNAc concentration was the highest among them without fructose and glucosamine accumulation, while the GlcNAc6P concentration remained consistently low. Results demonstrated that the substrate preference of phosphatase plays a crucial role in GlcNAc biosynthesis. In conclusion, we designed a phosphatase mutant with significant selectivity and catalytic activity, enabling efficient GlcNAc synthesis in vitro and in living cells.

## 3 Conclusion

In this study, a DBTL framework for efficiently guiding rational enzyme engineering based on combining the force field‐based method with QM simulations was applied to improve the catalytic efficiency of phosphatase to promote GlcNAc biosynthesis. The positive single‐point mutant was obtained under the guidance of fast mutant binding energy prediction using the force field method. Then, the mechanism underlying the substrate preference shift was revealed by elucidating the reaction pathway and analyzing high‐energy transition state conformations. Subsequent iterative designs aimed at stabilizing the transition state conformation to reduce the reaction energy barrier further enhanced the catalytic efficiency. Finally, the obtained phosphatase mutant with high activity and selectivity exhibited powerful potential for promoting GlcNAc biosynthesis in vitro and in living cells. This approach eliminated the necessity of exploring various combinations of multipoint mutations and the dependence on high‐throughput screening protocols. With continuous advancements in algorithms, it is anticipated that our framework will more efficiently explore the potential of protein catalysts.

## 4 Experimental Section

### Strains and Plasmids

Unless otherwise stated, all chemicals used in this study were purchased from commercial suppliers and used as received. *E. coli* BL21 (DE3) and *DH5α* were used as the expression and cloning hosts, respectively. The GlcNAc production strain was a genetically engineered *E. coli* constructed in a previous study.^[^ ^25^ ^]^ It involved the knockout of *nagABE*, *manXYZ*, L‐fucose isomerase (*fucI*), L‐fuculokinase (*fucK)*, D‐lactate dehydrogenase and pyruvate dehydrogenase. Additionally, two copies of T7‐lacO‐*GNA1* and one of T7‐lacO‐*glmS* were integrated at the *manXYZ*, *nagABE*, and *fucIK* sites. The pET‐28a plasmid was used for protein expression, whereas the pKD46 and pCP20 plasmids were used for gene integration.

### Intracellular Phosphosugar Detection

Acetonitrile/methanol/water (H_2_O; 40:40:20) solution was used to quench and extract whole‐cell samples. After determining the total metabolite concentration for overnight and freeze‐dried samples, the samples were resuspended in H_2_O and centrifuged (10000 × *g*, 15 min) before measuring intracellular metabolite concentrations. Ultra‐high‐performance liquid chromatography (UHPLC)–tandem mass spectrometry (MS) was used to detect the metabolites.

An Agilent 1290 Infinity II UHPLC system (Agilent Technologies) was used for LC. The column oven temperature was 30 °C. A 0.2‐µm inline filter (Agilent Technologies) and an Amide column (ACQUITY UPLC BEH Amide 1.7 µm, 2.1 mm × 100 mm) were used. The injection volume for all methods was 3 µL. LC solvent A was H_2_O with 10 mm ammonium acetate (NH_4_CH_3_CO_2_) and 0.3% (v/v) ammonium hydroxide (NH_4_OH). LC solvent B was acetonitrile/H_2_O (9:1) with 10 mm ammonium acetate and 0.3% (v/v) NH_4_OH. The gradients were 0 min 100% B, 1.75 min 89% B, 5 min 80% B, 6 min 65% B, 7 min 65% B, 8 min 100% B, and 15 min 100% B. The flow rate was 0.4 mL min^−1^.

An Agilent 6495C triple quadrupole MS (Agilent Technologies) was used for MS. The source gas temperature was set to 200 °C, with 14 L min^−1^ drying gas and a nebulizer pressure of 24 psi. The sheath gas temperature was set to 300 °C and the flow rate at 11 L min^−1^. Electrospray nozzle and capillary voltages were set to 500 and 2500 V, respectively. Dwell times of 50 ms were used for multiple reaction monitoring assays of four metabolites. The *m/z* of four metabolites were 300 > 199.1 (GlcNAc6P), 258 > 97.1 (GlcN6P), and 259 > 97.1 (Fru6P or Glc6P) in negative ionization mode. F6P and G6P were structural isomers. With reference to specific literature, the liquid‐phase method was optimized, the two compounds were successfully separated, and a signal‐to‐noise ratio meeting the quantification limit was achieved (Figure S5b, Supporting Information).^[^ ^26^ ^]^

### Protein Expression and Purification

Recombinant *E. coli* cells were cultured at 37 °C in Luria–Bertani (LB) medium (containing 10 g L^−1^ tryptone, 5 g L^−1^ yeast extract, and 10 g L^−1^ NaCl) supplemented with kanamycin (50 µg mL^−1^). When the optical density at 600 nm (OD_600 nm_) reached 0.8–1.0, IPTG was added to a final concentration of 0.4 mm to induce protein expression. After incubation at 16 °C for 16 h, cells were harvested, washed twice, and suspended in 50 mm HEPES (pH 7.0). The suspended cells were lysed by sonication and centrifuged at 9000 × *g* at 4 °C for 30 min.

For further purification, the crude enzyme solution was loaded onto a Ni‐NTA (nickel ion affinity chromatography) superflow column at a 1 mL min^−1^ flow rate. Unbound proteins were eluted with a washing buffer (50 mm HEPES, 50 mm NaCl, 50 mm imidazole, pH 7.0), and the target proteins were eluted with an elution buffer (50 mm HEPES, 50 mm NaCl, 200 mm imidazole, pH 7.0).

### Enzyme Activity Assay And Kinetic Analysis

Before conducting kinetic studies, the recombinant enzyme was dialyzed against 50 mm HEPES (pH 7.0) containing 5 mm MgCl_2_. Steady‐state kinetic parameters (*K*_m_ and *k*_cat_) for phosphorylated substrates were determined by measuring initial reaction velocities at different substrate concentrations (1–80 mm). Protein concentrations were determined using the A280 method, and absorbance measurements were performed with a Perkin–Elmer λ25 ultraviolet‐visible spectrophotometer. These initial velocities were measured in 50 mm HEPES buffer (pH 7.0) containing 0.1–1 µm enzyme and 5 mm MgCl_2_; an 80‐µL reaction mixture was added to a 96‐well plate, and the plate was incubated at 37°C with shaking at 750 rpm for 10 min. Phosphatase activity was tested using the Malachite Green Phosphate Assay Kit (Catalog No. MAK307) from Sigma‐Aldrich.

Data were fitted to the Michaelis–Menten equation:

(1) $$\;v_{\left(0\right)}=v_{\max}\;\frac{x}{K_{m}+x}$$

where *v*_(0)_ is the initial velocity, *v*_*max*_ is the maximum velocity, *x* is the substrate concentration, and *K_m_* is the Michaelis–Menten constant for the substrate. The *k*_cat_ value was calculated from *v*_*max*_and the protein subunit concentration [E] in the assay using the following equation: *k*_cat_ = *v*_*max*_/[E].

### Fluorescence Intensity Assay

Recombinant *E. coli* strains containing fluorescent proteins were precultured in LB medium for 10 h and inoculated into 200‐µL LB medium at a 1% ratio in 96‐well plates (Corning 3603). The 96‐well plates were cultured at 37 °C with shaking at 750 rpm. The fluorescence of super fold‐green fluorescent proteins (sfGFP) was detected by a Cytation microplate reader (BioTek; excitation, 480 nm; emission, 516 nm).

To calculate the relative fluorescence intensity, the background optical density of the medium (ODbg) and the background fluorescence of the strain without fluorescent protein expression (FPbg) were subtracted and the following formula was applied for correction:

(2) $$\;{\left(\frac{\mathrm{FP}}{\mathrm{OD}}\right)}_{\mathrm{corrected}}=\frac{\mathrm{FP}-FP_{bg}}{\mathrm{OD}-OD_{bg}}\;$$

### Molecular Docking

Hydrophobicity analysis of the binding pocket was performed using Discovery Studio 2022. The molecular docking module in Schrödinger 2021–4 was used to predict the conformation of the enzyme–substrate complex. The substrate‐binding pocket location and volume were predicted and calculated using the SiteMap module. The center of the molecular docking box was determined at the mass centers of D10, P46, I50, N69, D211, and N214 in the crystal structure of BT4131 (PDB ID: 1YMQ).

### MD Simulations

MD simulations were performed using Desmond for the enzyme without a substrate, employing the OPLS2005 force field.^[^ ^27^ ^]^ The modeling system was enclosed within a cubic box filled with a SPC water model (with a box boundary 10 Å away from the enzyme). Subsequently, the system was neutralized using Na^+^ or Cl^−^ and subjected to energy minimization using the steepest descent method. Finally, a 200 ns MD simulation was conducted at 310.15 K. Atomic trajectories were analyzed using Maestro to generate RMSF, RMSD, and distances between specified atoms.

### QM Calculations

QM calculations of active sites for WT‐Glc6P, WT‐GlcNAc6P, M1‐Glc6P, and M1‐GlcNAc6P were conducted using the Jaguar module of Schrödinger 2021–4. The QM region was defined to include residues 8–10, 43–45, 49, 129, 174, 178, 180, 188, 211, and 214, in addition to the Mg^2+^ ion and the two H_2_O molecules coordinated with it. To obtain more accurate energies, single‐point calculations were performed using the 6–31G** basis set. Dispersion effects were evaluated using the B3LYP‐D3 method. The amino acid residues were truncated at α‐carbons, and hydrogen atoms were added to saturate the model. The positions of truncated carbons were fixed during the conformation optimization to prevent unrealistic movement. In the energy scanning, the distance range between the oxygen atoms in the phospholipid bonds (OF) of the phosphosugar substrate and the H^+^ provided by D10 was chosen to be from 1.58 to 0.98 Å, and the distance range between the OF and the phosphorus atom was from 1.65 Å to their distance in the final product. The scanning method was relaxed scanning, optimizing each conformation at each process step.

### Residue Mutation Scanning In Silico

The stability and substrate affinity of mutants were predicted by the residue scanning calculation module of Schrödinger 2021–4. The refinement method was set to “side‐chain prediction with backbone sampling,” and the cutoff was set to 5 Å.

### Gene Integration

Each phosphatase gene was integrated at *ldhA* sites. Through λ‐Red homologous recombination (HR), the integration of BT4131 and mutants was achieved. HR fragments required for integration had to be constructed first. Gene integration required ligating four DNA fragments, including the upstream homology arm, the downstream homology arm, the target gene, and the resistance gene, with FRT sites at both ends. Because there were fewer fragments in this case, overlapping extension polymerase chain reaction (PCR) could be used to achieve efficient ligation. In the region where the segments needed to be connected, a suitable overlap region was designed based on the annealing temperature. Once all fragments were obtained, further fusion amplification could be performed to obtain the required recombinant fragments. When integrating genes into the genome, four or more fragments were required for ligation. Overlapping extension PCR was challenging to ensure efficiency and accuracy. The seamless cloning kit was used to ligate multiple fragments into one vector. Then, PCR was used to amplify the required recombinant fragments. In this study, the 800–1000‐bp homology arm could achieve effective recombination efficiency for knockout or integration.

To induce the expression of the λ‐Red HR system, the strain with pKD46 was cultured in LB medium with 50 mm arabinose for >1 h at 30 °C until the OD_600 nm_ reached 0.6. Thereafter, it could be transformed into electrotransformed or chemically transformed competent cells. The previously constructed HR fragments were transformed into these competent cells. The successfully knocked‐out or integrated strains were selected through colony PCR, and the pCP20 plasmid was transformed into these strains to eliminate the antibiotic resistance gene flanked by FRT sites. Both pKD46 and pCP20 plasmids were temperature‐sensitive plasmids. To eliminate these two plasmids, the strains could be cultured in an antibiotic‐free LB medium at 37–42 °C overnight.

### Fed‐Batch Fermentation in a 30‐L Bioreactor

The seed medium contained 10 g L^−1^ glucose, 3 g L^−1^ yeast extract, 1 g L^−1^ glycerin, 8 g L^−1^ KH_2_PO_4_, 10 g L^−1^ K_2_HPO_4_, 1 g L^−1^ citric acid monohydrate, 0.5 g L^−1^ MgSO_4_·7H_2_O, 5 g L^−1^ (NH_4_)_2_SO_4_, 0.02 g L^−1^ CaCl_2_, and 1 mL L^−1^ mixture of trace elements. The trace element mixture included 0.1 g L^−1^ CoCl_2_·6H_2_O, 0.1 g L^−1^ CuSO_4_·5H_2_O, 5 g L^−1^ FeSO_4_·7H_2_O, 0.33 g L^−1^ MnSO_4_·H_2_O, and 3.8 g L^−1^ ZnSO_4_·7H_2_O. The pH was adjusted to 7.0 by adding ammonia. The colonies on the plates grown overnight were cultured in liquid LB medium for 10–12 h at 37 °C and 220 rpm. According to the 3% inoculum volume, it was transferred to a baffled shake flask containing fresh seed culture medium and incubated at 37 °C for 10–12 h at 220 rpm.

The 50‐L bioreactor fermentation medium contained 5 g L^−1^ glucose, 4.8 g L^−1^ yeast extract, 1 g L^−1^ glycerin, 6.67 g L^−1^ KH_2_PO_4_, 2.8 g L^−1^ K_2_HPO_4_, 3.5 g L^−1^ citric acid monohydrate, 2.48 g L^−1^ MgSO_4_·7H_2_O, 4 g L^−1^ (NH_4_)_2_SO_4_, 0.02 g L^−1^ CaCl_2_, 0.1 mg L^−1^ V_H_, 0.5 mg L^−1^ V_B1_, and 1 mL L^−1^ mixture of trace elements. The seeds were added to the bioreactor at an inoculation rate of 10%, and the total volume after inoculation was 15 L. The pH was automatically adjusted by adding ammonia water, and it was always maintained at ≈7.0 during the fermentation process. By adjusting the ventilation, the stirring speed and the glucose flow acceleration were controlled to maintain the dissolved oxygen at 30%–50%. After the initial glucose was exhausted, a 65% glucose solution was fed into the bioreactor. The fermentation process lasted 55–65 h at 37 °C. Fed‐batch cultivation was performed with an initial glucose concentration of 5.0 g L^−1^. When glucose was depleted, 14–17‐L concentrated glucose (850 g L^−1^) was added to the medium. The expression of key genes and phosphatase was induced by adding IPTG to a final concentration of 0.4 mm after culturing for 8 h.

### GlcNAc Analytic Methods

The GlcNAc concentrations in the fermentation broth were measured via HPLC using an HPX‐87H column (Bio‐Rad Hercules, CA, USA) and a refractive index detector. Dilute sulfuric acid at a concentration of 5 mm was used as the mobile phase with a flow rate of 0.6 mL min^−1^ at 35 °C. The glucose and glutamate concentrations in the supernatant were measured using a glucose–glutamate analyzer (SBA‐40C; Biology Institute of Shandong Academy of Sciences, Jinan, China).

### Kinetic Model Simulation

To perform a dynamic simulation of the relative concentrations of the concerned metabolites or the relative fluxes in the concerned pathways, the linear pathway kinetic model based on the Michaelis–Menten equation was employed. The construction of kinetic models and the performance of all dynamic simulations were achieved in MATLAB R2019a. The linear pathway is shown in Figure 6a. In the model, x_(1)_–x_(8)_ represent the relative concentrations of the concerned metabolites or the relative fluxes in the concerned pathways, and v_(1)_–v_(12)_ are the reaction rates for each step of the enzymatic reaction. The value of v_max‐Glc6P_ of WT was set at 0.5, and the v_max_ for other substrates and mutants was represented as 0.5 by multiplying the ratio of the respective ratios of their actual value with v_max‐Glc6P_ of WT. The v_max_ value for other nonphosphatase‐catalyzed reactions ranged from 0.6 to 1. *K*_m_ parameters of nonphosphatase‐catalyzed reactions were randomly sampled between 0.5 and 2.0. The *K*_m‐Glc6P_ of WT was randomly sampled between 0.5 and 2.0, and the *K*_m_ values for other substrates and mutants were represented by multiplying the ratio of their actual values with the *K*_m‐Glc6P_ of WT by this range. To describe the kinetics of reactions, Michaelis–Menten kinetics was used as follows:

(3) $$\;v_{\left(1\right)}=v_{\left(1\right)\max\;}$$

(4) $$\;v_{\left(2\right)}=v_{\left(2\right)\max\;}\;\frac{x_{\left(1\right)}}{K_{m\left(2\right)}+x_{\left(1\right)}}$$

(5) $$\;v_{\left(3\right)}=v_{\left(3\right)\max\;}\;\frac{x_{\left(2\right)}}{K_{m\left(3\right)}+x_{\left(2\right)}}$$

(6) $$\;v_{\left(4\right)}=v_{\left(4\right)\max\;}\;\frac{x_{\left(3\right)}}{K_{m\left(4\right)}+x_{\left(3\right)}}$$

(7) $$\;v_{\left(5\right)}=v_{\left(5\right)\max\;}\;\frac{x_{\left(2\right)}}{K_{m\left(5\right)}+x_{\left(2\right)}}$$

(8) $$\;v_{\left(6\right)}=v_{\left(6\right)\max\;}\;\frac{x_{\left(3\right)}}{K_{m\left(6\right)}+x_{\left(3\right)}}$$

(9) $$\;v_{\left(7\right)}=v_{\left(7\right)\max\;}\;\frac{x_{\left(4\right)}}{K_{m\left(7\right)}+x_{\left(4\right)}}$$

(10) $$\;v_{\left(8\right)}=v_{\left(8\right)\max\;}\;\frac{x_{\left(7\right)}}{K_{m\left(8\right)}+x_{\left(7\right)}}$$

(11) $$\;v_{\left(9\right)}=v_{\left(9\right)\max\;}\;\frac{x_{\left(4\right)}}{K_{m\left(9\right)}+x_{\left(4\right)}}$$

(12) $$\;v_{\left(10\right)}=v_{\left(10\right)\max\;}\;\frac{x_{\left(8\right)}}{K_{m\left(10\right)}+x_{\left(8\right)}}$$

(13) $$\;v_{\left(11\right)}=v_{\left(11\right)\max\;}\;\frac{x_{\left(7\right)}}{K_{m\left(11\right)}+x_{\left(7\right)}}$$

(14) $$\;v_{\left(12\right)}=v_{\left(12\right)\max\;}\;\frac{x_{\left(5\right)}}{K_{m\left(12\right)}+x_{\left(5\right)}}$$

Mass balances for all metabolites result in differential equations:

(15) $$\;\frac{dx_{\left(1\right)}}{dt}=v_{\left(1\right)}\;-v_{\left(2\right)}+v_{\left(5\right)}$$

(16) $$\;\frac{dx_{\left(2\right)}}{dt}=v_{\left(2\right)}\;-v_{\left(3\right)-}v_{\left(5\right)}$$

(17) $$\;\frac{dx_{\left(3\right)}}{dt}=v_{\left(3\right)}\;-v_{\left(4\right)}-v_{\left(6\right)}+v_{\left(9\right)}+v_{\left(12\right)}$$

(18) $$\;\frac{dx_{\left(4\right)}}{dt}=v_{\left(4\right)}\;-v_{\left(7\right)}+v_{\left(8\right)}-v_{\left(9\right)}-v_{\left(10\right)}$$

(19) $$\;\frac{dx_{\left(5\right)}}{dt}=v_{\left(6\right)}\;-v_{\left(12\right)}$$

(20) $$\;\frac{dx_{\left(6\right)}}{dt}=v_{\left(7\right)}\;$$

(21) $$\;\frac{dx_{\left(7\right)}}{dt}=v_{\left(10\right)}\;-v_{\left(8\right)}-v_{\left(11\right)}$$

(22) $$\;\frac{dx_{\left(8\right)}}{dt}=v_{\left(11\right)}\;$$

Hill kinetics was employed to describe the regulation of a GlcNAc6P‐responsive biosensor. The equation for reactions v_(5)_–v_(7)_ and v_(11)_ catalyzed by phosphatases is as follows. The Hill coefficient *n* was sampled between 0 and 4, *p* was set as the initial v_max_ of each reaction, and *K_a_* was between 0 and 1.

(23) $$\;v_{5}=\;\left(p+v_{\left(5\right)\max\;}\frac{{x_{\left(7\right)}}^{n}}{{K_{a}}^{n}+{x_{\left(7\right)}}^{n}}\right)\frac{x_{\left(2\right)}}{K_{m\left(5\right)}+x_{\left(2\right)}}$$

(24) $$\;v_{6}=\;\left(p+v_{\left(6\right)\max\;}\frac{{x_{\left(7\right)}}^{n}}{{K_{a}}^{n}+{x_{\left(7\right)}}^{n}}\right)\frac{x_{\left(3\right)}}{K_{m\left(6\right)}+x_{\left(3\right)}}$$

(25) $$\;v_{7}=\;\left(p+v_{\left(7\right)\max\;}\frac{{x_{\left(7\right)}}^{n}}{{K_{a}}^{n}+{x_{\left(7\right)}}^{n}}\right)\frac{x_{\left(4\right)}}{K_{m\left(7\right)}+x_{\left(4\right)}}$$

(26) $$\;v_{11}=\;\left(p+v_{\left(11\right)\max\;}\frac{{x_{\left(7\right)}}^{n}}{{K_{a}}^{n}+{x_{\left(7\right)}}^{n}}\right)\frac{x\left(7\right)}{K_{m\left(11\right)}+x\left(7\right)}$$

Hill kinetics was also employed to describe the impact of phosphosugar pressure on glucose transport. The Hill coefficient n was sampled between 0 and 4, and Ki was between 0 and 1.

(27) $$\;v\left(1\right)=v\left(1\right)max\;\;\left(\frac{{K_{i}}^{n}}{{K_{i}}^{n}+{\left(x_{\left(2\right)}+x_{\left(3\right)}+x_{\left(4\right)}+x_{\left(7\right)}\right)}^{n}}\right)$$

### Statistical Analysis

All experiments were independently conducted at least three times, and the data were displayed as mean values ± standard deviation (SD). GraphPad Prism 9.0.0 was used for statistical analysis. Differences between two groups were determined by two‐tailed Student's *t*‐test, and one‐way ANOVA followed by post‐hoc Dunnett's test for multiple groups. Statistical significance is indicated as * for *P* < 0.05 and ** for *P* < 0.01, respectively.

## Conflict of Interest

The authors declare no conflict of interest.

## Supporting information

Supporting Information

## Data Availability

The data that support the findings of this study are available from the corresponding author upon reasonable request.
