# Supplementary material for: Reshaping Phosphatase Substrate Preference for Controlled Biosynthesis Using a “Design–Build–Test–Learn” Framework
Source: Adv Sci (Weinh). 2024 Mar 19;11(22):2309852. doi: 10.1002/advs.202309852 (PMC11165480; doi:10.1002/advs.202309852)
Supplement: Supplementary file 1 — Supporting Information [file ADVS-11-2309852-s001.pdf]

ADVANCED SCIENCE Open Access

Supporting Information

for *Adv. Sci.*, DOI 10.1002/advs.202309852

Reshaping Phosphatase Substrate Preference for Controlled Biosynthesis Using a "Design–Build–Test–Learn" Framework

*Jiangong Lu, Xueqin Lv, Wenwen Yu, Jianing Zhang, Jianxing Lu, Yanfeng Liu, Jianghua Li, Guocheng Du, Jian Chen and Long Liu**

Supporting Information

Reshaping Phosphatase Substrate Preference for Controlled Biosynthesis using a "Design–Build–Test–Learn" Framework

*Jiangong Lu, Xueqin Lv, Wenwen Yu, Jianing Zhang, Jianxing Lu, Yanfeng Liu, Jianghua Li, Guocheng Du, Jian Chen, Long Liu**

Jiangong Lu, X. Lv, W. Yu, J. Zhang, Y. Liu, G. Du, L. Liu

Key Laboratory of Carbohydrate Chemistry and Biotechnology, Ministry of Education

Jiangnan University

Wuxi 214122, China.

E-mail: longliu@jiangnan.edu.cn

Jiangong Lu, X. Lv, W. Yu, J. Zhang, Y. Liu, J. Li, G. Du, J. Chen, L. Liu

Science Center for Future Foods,

Jiangnan University,

Wuxi 214122, China.

Jianxing Lu

Shandong Runde Biotechnology co., LTD

Taian 271200, China.

Contents

Supplementary Figures 1-7

Supplementary Tables 1-4

Supplementary Notes 1-2

Supplementary Figures

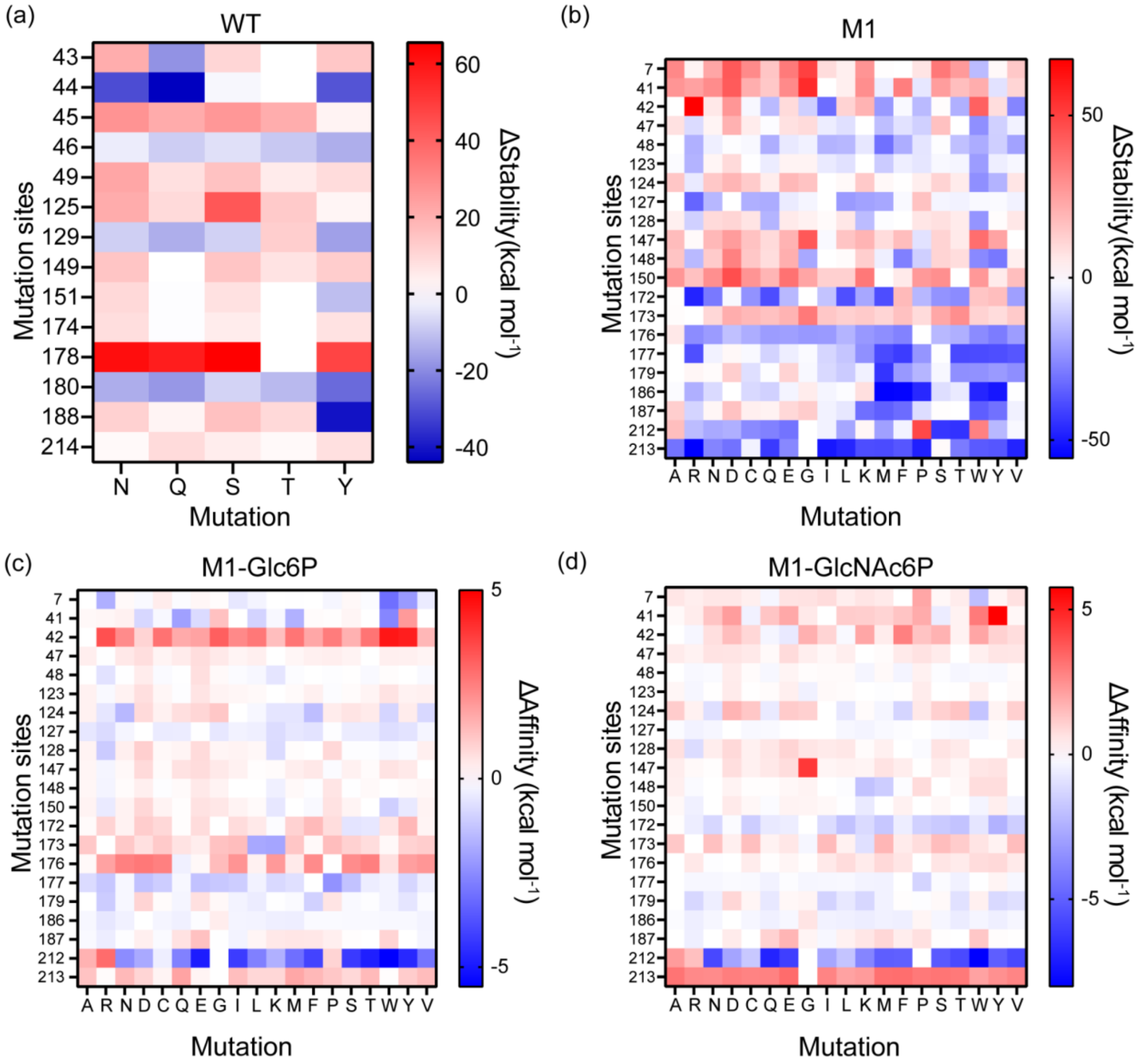

Figure S1. *In silico* Δaffinity and Δstability scanning of mutants. (a) The calculation results of Δstability of residue mutations of the binding pocket. (b) The calculation results of Δstability of upstream residue mutations on the binding pocket. (c) The calculation results of Δaffinity of upstream residue mutations on the binding pocket taking Glc6P as a substrate. (d) The calculation results of Δaffinity of upstream residue mutations on the binding pocket taking GlcNAc6P as a substrate.

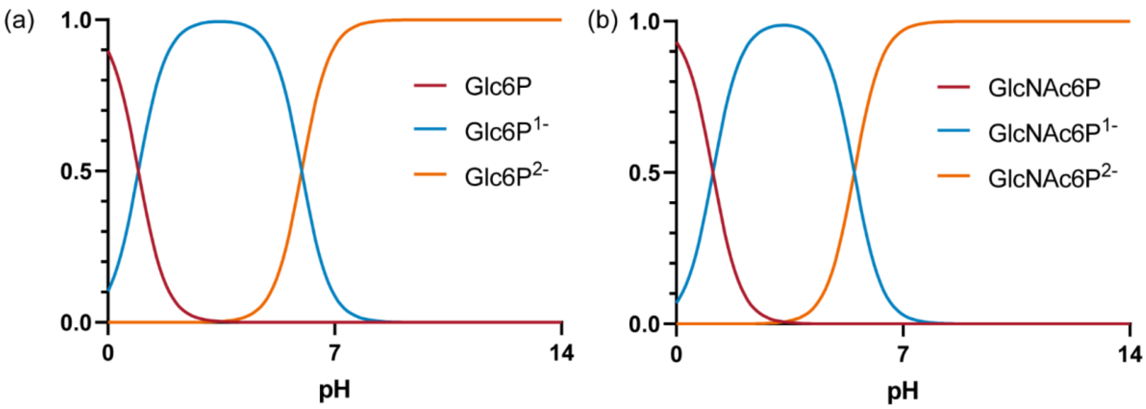

Figure S2. Variation in protonation state distribution of Glc6P and GlcNAc6P with changing pH. (a) Represents the protonation state distribution of Glc6P. (b) Represents the protonation state distribution of GlcNAc6P. The pKa values are obtained through QM calculations. Glc6P has a pKa1 of 1.54 and a pKa2 of 6.11. GlcNAc6P has a pKa1 of 1.13 and a pKa2 of 5.49.

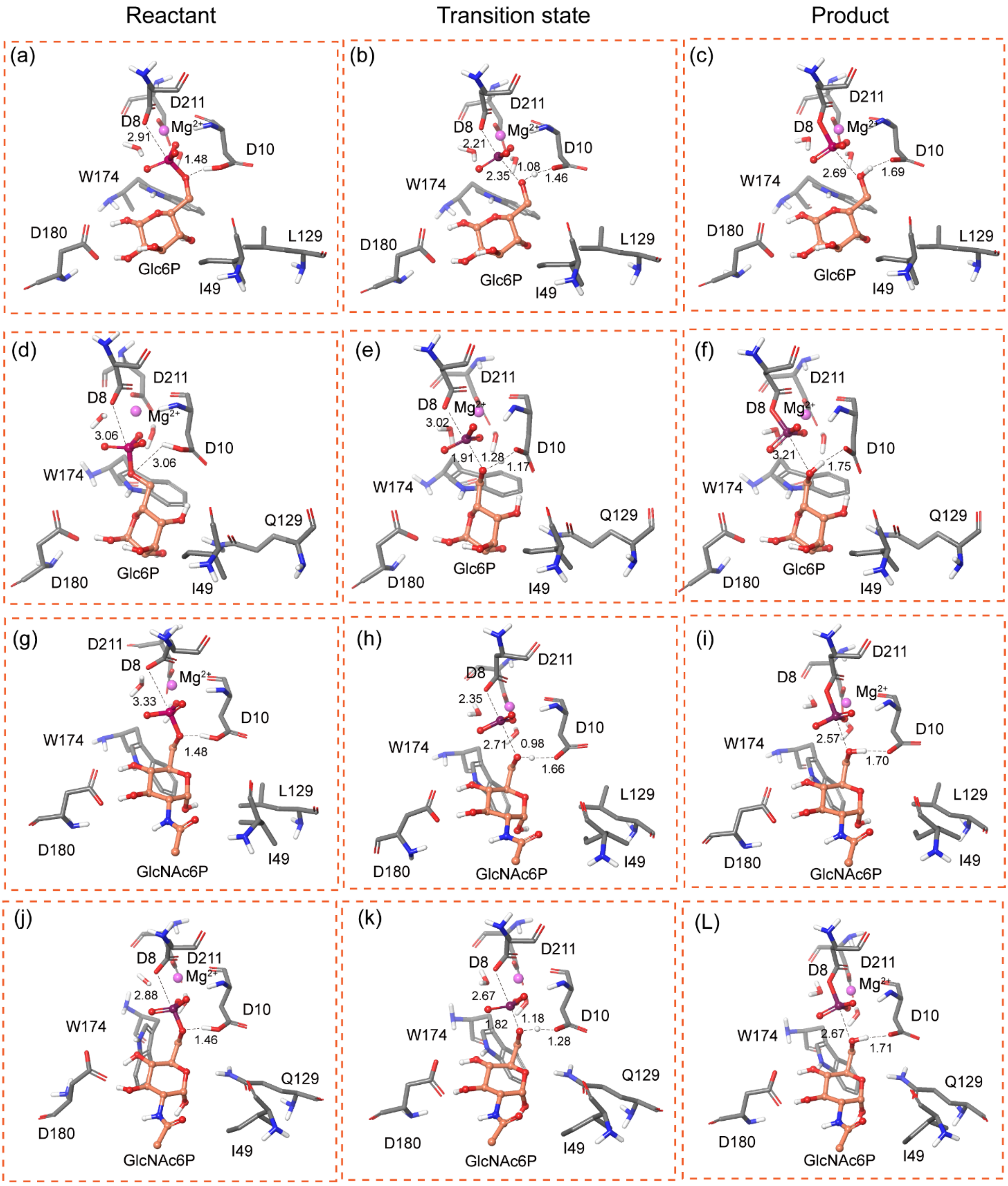

Figure S3. The reactant, transition state, and product conformations obtained through QM calculations for conformational optimization and potential energy surface scanning. (a)(b)(c) Corresponds to WT-Glc6P, (d)(e)(f) Corresponds to M1-Glc6P, (g)(h)(i) Corresponds to WT-GlcNAc6P, and (j)(k)(l) corresponds to M1-GlcNAc6P.

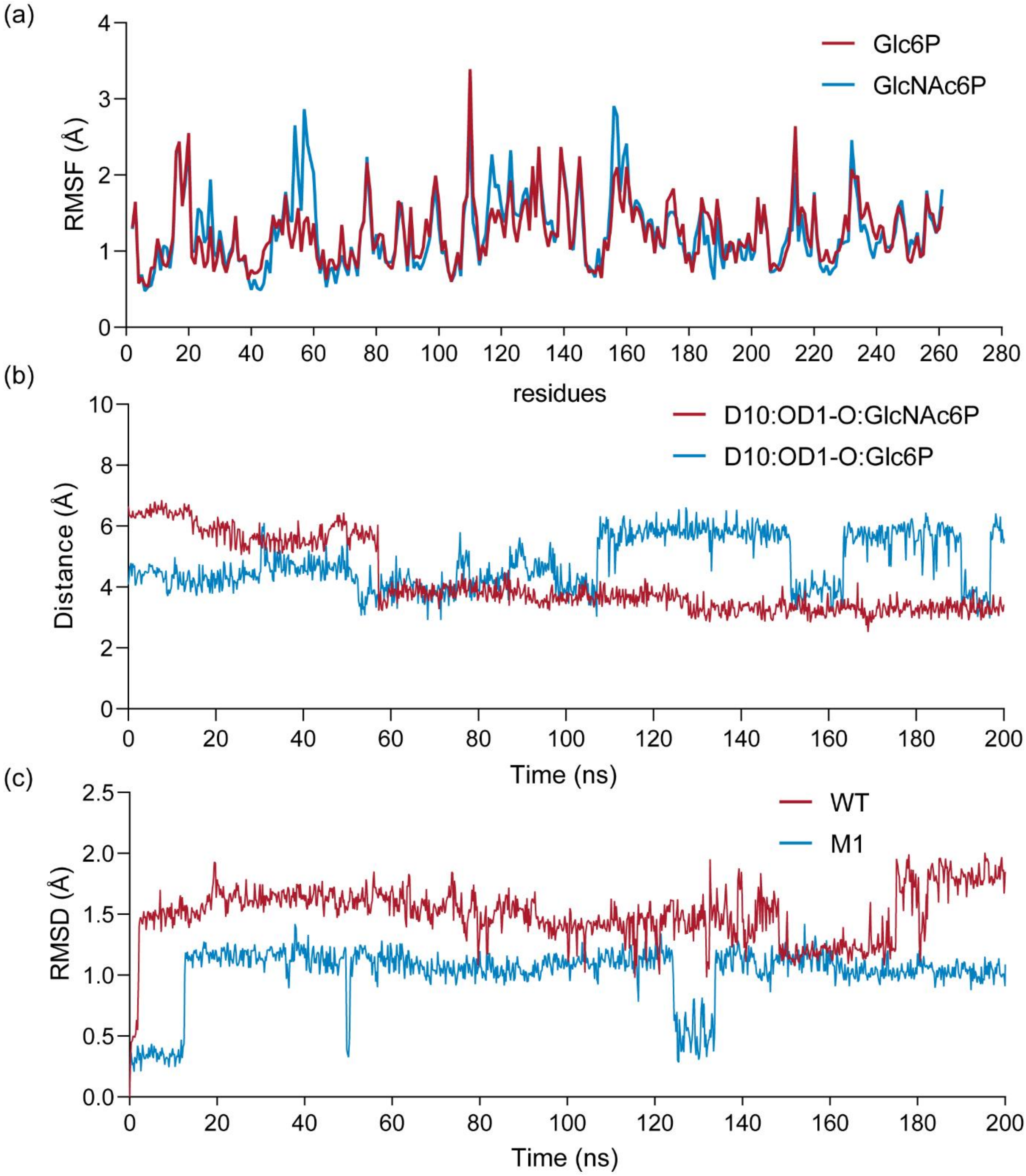

Figure S4. Molecular dynamics simulations of WT-Glc6P, M1-Glc6P, and M1-GlcNAc6P. (a) The RMSF during the simulation of M1-Glc6P and M1-GlcNAc6P. (b) The distance between OD1 of D10 and the OF of phosphosugar substrate during the simulation of M1-Glc6P and M1-GlcNAc6P.(c) The RMSD values of the substrate ligands during the simulation of WT-Glc6P and M1-Glc6P.

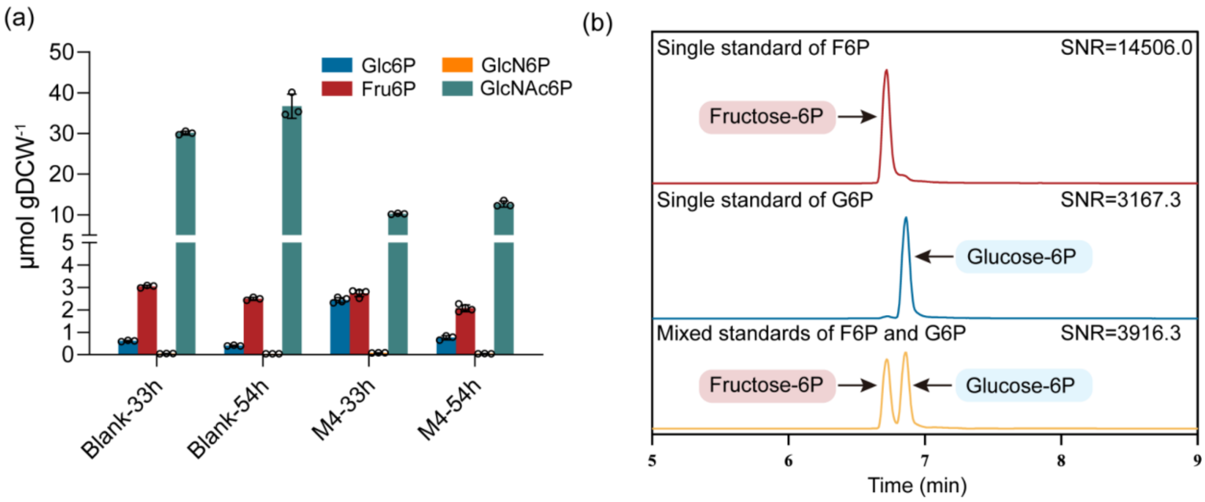

Figure S5. Measurement of intracellular phosphate sugar concentration in the GlcNAc-producing strain integrated M4. (a) Measurement of intracellular phosphosugar concentration in the GlcNAc-producing strain before and after M4 integration. (b) LC-MS analysis of Fru6P and Glc6P. Structural isomers Fru6P and Glc6P were successfully separated, and a signal-to-noise ratio (SNR) meeting the quantification limit was achieved. Data presented as mean values ± SD from three independent biological replicates (n = 3). Black circles represent individual data points.

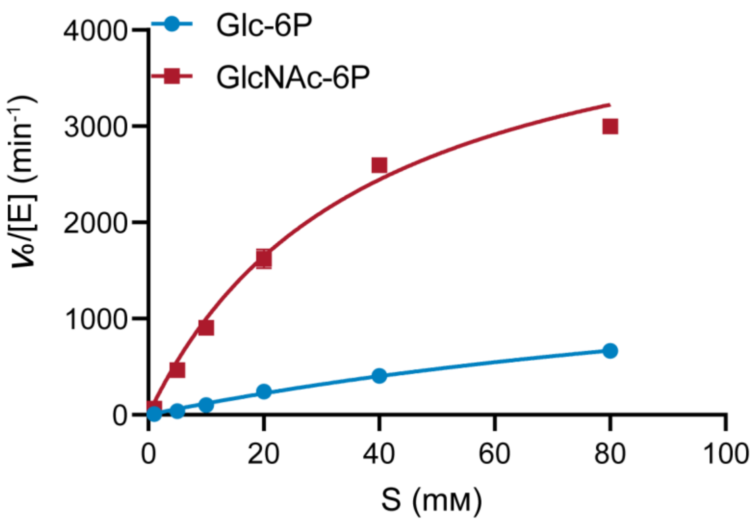

Figure S6. In vitro assay of catalytic activity of GlcNAc6P and Glc6P by M4. Data presented as mean values ± SD from three independent biological replicates (n = 3).

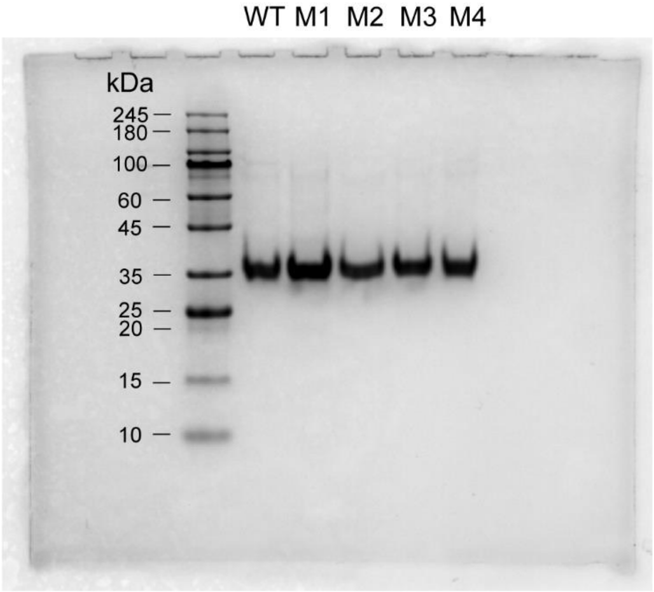

Figure S7. SDS-PAGE analysis.

Supplementary Tables

Table S1. Primers used in the experimental process

Primer	Sequence
L129Q.for	AAAAAATTTTTTATGATTTTCAGCATGTGAACGTGATTCCGACCG
L129Q.rev	AAAATCATAAAAAATTTTTTTCACCATTTCGTTCGGC
I49Q.for	TTTTTATTGCGACCGGCCGCCCGAAAGCGCAAATTAACAACCTG AGCGAACTGCA
I49Q.rev	GCGGCCGGTCGCAATAAAAA
G172L.for	CGCTGGTATCCGGCGTTTGC
G172L.rev	GCAAACGCCGGATACCAGCGCAGAATTTCGCAGGTCGGAATGCT C
ldha-up.for	ATATAGTTCCTCCTTTCAGCTAGCGAACAGTCACTTGCCG
ldha-up.rev	CGTCAATACCAGTCGCGGTG
PnagB.for	CACCGCGACTGGTATTGACGGTCACCAAATAATTCGCGAAGATA ATTAAGTTACTG
PnagB.rev	TATTCACCTCAATAAGTAAAATGTAAGCCGTTGG
BT4131.for	TTTACTTATTGAGGTGAATAATGACCAAAGCGCTGTTTTTTGATA TTG
BT4131.rev	ATTGCGGTATCGCACTGGATTTAGATGATACCAAAGTGTTTCATC GCTTTG
CMR-FRT.For	ATCCAGTGCGATACCGCAATG
CMR-FRT.Rev	GTTGTAGGCTGGAGCTGCTTC
ldha-down.for	AAGCAGCTCCAGCCTACAACGATCATAGGCTGGAACACGGACT
ldha-down.rev	TAGTAGGTTGAGGCCGTTGAGCAATTTCGCCAGACAAGCAGA
pnagB-laco.for	TGTTATCCGCTCACAATTCCGACAGGCGTCAATCCGACC
pnagB-laco.rev	GGAATTGTGAGCGGATAACAATTCCTGCGCAAATCCAGGTTACG CTTA

sfgfp-28a.rev	ATATAGTTCCTCCTTTCAGCTTATTTGTAGAGCTCATCCATGCCATGT
sfgfp-nag.for	ATGAGCAAAGGAGAAGAACTTTTCACT
yqaB.for	TTTACTTATTGAGGTGAATAATGTACGAGCGTTATGCAGGTTTAAT
yqaB.rev	ATTGCGGTATCGCACTGGATTCACAGCAAGCGAACATCCAC
yihX.for	TTTACTTATTGAGGTGAATAATGCTCTATATCTTTGATTTAGGTAATGTGATTGTC
yihX.rev	ATTGCGGTATCGCACTGGATTTAGCATAACACCTTCGCGAAATAGTCC
BT1666.for	TTTACTTATTGAGGTGAATAATGATCAAGGTTCTGTTACTGGATGTGG
BT1666.rev	ATTGCGGTATCGCACTGGATTCAAATTACGCCGAAGTGCTTTAATGC

Table S2. RBS sequences used in the construction of GlcNAc6P-responsive biosensor

RBS	Sequence
RBSnagB	TATTCACCTCAATAAGTAAAATGTAAGCCGTTGGCGGATTAGGCATCTTTAAGCGTAACCTGGATTTGCGCA
RBS1	CATGGTATATCTCCTTCTTAAAGTTAAACAAAATTATTTCTAGAGG
RBS2	CATGGTATATTTCTCCTCTTTATTATTTCTAGAGG
RBS3	CATGGTATAACCTCCTTAATTATTTCTAGAGG
RBS4	CATGGTATACCTCCTTTATTATTTCTAGAGG

Table S3. The k_{cat} value determined under shaking at 750 rpm at 37°C. [a)]

k_{cat}(95%CI) (min^{-1})	WT	M1	M2	M3	M4

Glc6P	552.4 (490.5-628.1)	573.7 (480.6-709.2)	1454.4 (1176.2-1874.0)	853.8 (648.5-1258.7)	1985.2 (1589.4-2679.6)
Fru6P	396.6 (337.9-479.2)	85.0 (70.8-105.5)	178.1 (151.1-214.4)	55.2 (41.6-81.2)	—[b)]
GlcN6P	7.1 (6.0-8.5)	89.2 (79.4-100.1)	448.9 (411.8-491.5)	8.1 (6.6-9.8)	—[b)]
GlcNAc6P	364.1 (326.0-410.7)	1084.1 (1021.7-1155.3)	2525.5 (2151-3057)	3913.3 (3544.0-4366.7)	4735.3 (4113.0-5553.6)

[a)] Kinetic parameters were determined in an 80-μL reaction system containing 50 mM HEPES, 5 mM $MgCl_2$, 0.1–1 μM purified enzyme, and 1–80 mM substrate. The reaction was carried out at 37°C with shaking at 750 rpm for 10 min. See the Experimental Methods for experimental details. [b)] "—" means not tested.

Table S4. The K_m value determined under shaking at 750 rpm at 37°C.[a]

K_m(95%CI) (mM)	WT	M1	M2	M3	M4
Glc6P	17.83 (12.79-24.94)	50.69 (36.23-73.42)	31.35 (19.30-52.38)	69.97 (42.87-128.50)	157.80 (115.40-233.70)
Fru6P	39.36 (27.78-57.31)	31.40 (20.48-49.63)	24.58 (16.28-37.67)	37.55 (19.85-78.61)	—[b)]
GlcN6P	3.42 (1.42-7.21)	4.69 (2.89-7.32)	17.03 (13.36-21.72)	3.86 (1.51-8.44)	—[b)]

GlcNAc6P	27.42 (20.95-36.30)	34.00 (29.56-39.26)	37.42 (26.23-55.22)	52.37 (43.53-63.69)	37.50 (28.25-50.45)

[a] Kinetic parameters were determined in an 80-μL reaction system containing 50 mM HEPES, 5 mM $MgCl_2$, 0.1–1 μM purified enzyme, and 1–80 mM substrate. The reaction was carried out at 37°C with shaking at 750 rpm for 10 min. See the Experimental Methods for experimental details. [b] "—" means not tested.

Supplementary Notes

Gene sequence of BT4131

ATGACCAAAGCGCTGTTTTTTGATATTGATGGCACCCTGGTGAGCTTTGAAACCCACCGCATTCCGAGCAGCACCATTGAAGCGCTGGAAGCGGCGCATGCGAAAGGCCTGAAAATTTTTATTGCGACCGGCCGCCCGAAAGCGATTATTAACAACCTGAGCGAACTGCAAGATCGCAACCTGATTGATGGCTATATTACCATGAACGGCGCGTATTGCTTTGTGGGCGAAGAAGTGATTTATAAAAGCGCGATTCCGCAAGAAGAAGTGAAAGCGATGGCGGCGTTTTGCGAAAAAAAAGGCGTGCCGTGCATTTTTGTGGAAGAACATAACATTAGCGTGTGTCAGCCGAACGAAATGGTGAAAAAAATTTTTTATGATTTTCTGCATGTGAACGTGATTCCGACCGTGAGTTTCGAAGAAGCGAGCAACAAAGAAGTGATTCAGATGACCCCGTTTATTACCGAAGAGGAAGAAAAAGAAGTGCTGCCGAGCATTCCGACCTGCGAAATTGGCCGCTGGTATCCGGCGTTTGCGGATGTGACCGCGAAAGGCGATACCAAACAGAAAGGCATTGATGAAATTATTCGCCATTTTGGCATTAAACTGGAAGAAACCATGAGCTTTGGCGATGGCGGCAACGATATTAGCATGCTGCGCCATGCGGCGATTGGCGTGGCGATGGGCCAAGCGAAAGAAGATGTGAAAGCGGCCGCGGATTATGTGACCGCGCCGATTGATGAAGATGGCATTAGCAAAGCGATGAAACACTTTGGTATCATCTAA

Cartesian coordinates of stationary points

The reactant, product and the transition state in catalytic mechanisms of the WT-Glc6P, WT-GlcNAc6P, M1-Glc6P and M1-GlcNAc6P

WT-G6P-R

N	8.73110	0.64600	43.62160
C	9.00640	-0.48470	42.72470
C	7.72990	-0.78740	41.93620
O	6.62000	-0.65680	42.45570
C	9.54560	-1.77880	43.37330
C	10.46230	-2.54130	42.39140
O	10.55300	-3.79830	42.55440
O	11.06540	-1.84880	41.53480
N	7.92260	-1.20160	40.65680
C	6.79160	-1.44920	39.77650
C	6.65740	-2.90680	39.33260
O	5.57720	-3.31100	38.89600
C	6.80890	-0.43300	38.59280
C	6.53450	0.97920	39.15720
C	5.81260	-0.77760	37.47700
C	6.92300	2.12560	38.21900
N	7.74820	-3.71220	39.43860
C	7.64180	-5.08140	39.02490
C	8.23510	-6.02790	40.02950
O	8.98240	-5.72610	40.93560
C	8.34960	-5.40800	37.66110
C	7.85640	-4.43670	36.61790
O	6.92180	-4.64220	35.85400
O	8.47540	-3.26070	36.59530
N	11.39590	1.62310	41.79220
C	11.46240	1.16000	40.39360
C	12.86970	1.46670	39.88210
O	13.45640	2.48990	40.25940
C	10.39660	1.82070	39.46740
O	10.41200	1.21440	38.18340
C	10.57150	3.32210	39.27640
N	13.36710	0.59700	38.97290
C	14.48140	0.96640	38.12190
C	14.07430	1.40650	36.70390
O	14.93900	1.58810	35.84700
N	12.74780	1.58090	36.47540
C	12.28500	2.06100	35.18730
C	12.71900	3.49910	34.90610
O	13.01850	4.31290	35.74670
C	10.74110	2.00070	35.11460
C	10.20520	0.56320	35.06230
C	8.69350	0.50740	35.32060
N	8.12130	-0.83470	35.14190
C	7.51440	-1.29330	34.05210
N	7.47280	-0.56230	32.91660
N	6.93320	-2.50120	34.03640
N	8.91810	3.22850	29.20540
C	8.43410	2.12610	30.03760
C	7.85410	2.73790	31.30290
O	7.73950	2.17990	32.37940
C	9.39650	0.93330	30.28790
C	10.64860	1.34440	31.09080
C	9.74670	0.25860	28.95440
C	11.57940	0.16990	31.41500
N	7.81260	-6.31990	28.52460
C	6.98860	-5.39480	29.30790
C	5.82360	-4.95300	28.41500
O	5.33730	-3.84280	28.42010
C	7.71970	-4.20300	29.96200
C	8.93410	-4.58110	30.83580
C	9.67650	-3.32490	31.32000
C	8.55400	-5.47180	32.02810
N	17.72210	-6.92430	39.50330
C	17.07160	-8.22750	39.70190
C	17.41230	-9.21820	38.58740
O	17.86580	-10.32850	38.77310
C	15.53600	-8.02390	39.88550
C	14.85150	-7.14840	38.87020
C	14.76150	-5.77600	38.92310
C	14.08810	-7.54950	37.71430
N	13.97140	-5.29920	37.89820
C	13.57250	-6.36290	37.11520
C	13.76580	-8.78860	37.13920
C	12.78650	-6.38900	35.96110
C	12.97870	-8.81950	35.99370

C	12.50000	-7.63080	35.40660	C	15.88630	-2.20520	41.14030
N	20.83380	-5.51160	31.42220	O	14.99340	-2.46720	41.97900
C	20.80420	-5.14930	32.84050	N	15.66460	-2.16500	39.82030
C	21.16910	-6.30470	33.76570	Mg	11.21350	-5.07050	41.11590
O	21.59400	-7.37450	33.38070	O	11.36710	-6.26850	39.40660
C	19.47070	-4.48200	33.25850	O	13.05480	-4.09240	40.79340
C	18.38130	-5.41150	33.77260	H	7.74550	0.59600	43.88040
C	18.12270	-6.67300	33.21650	H	9.77500	-0.19250	42.01370
C	17.59090	-4.99180	34.85170	H	8.75350	-2.43330	43.74660
C	17.10020	-7.48030	33.71830	H	10.16580	-1.51110	44.24330
C	16.56260	-5.79340	35.34890	H	8.85640	-1.20250	40.23910
C	16.30900	-7.04470	34.78260	H	5.89070	-1.27140	40.36960
N	21.56980	-2.37700	35.65650	H	7.82940	-0.46170	38.19070
C	21.11900	-1.00380	35.85370	H	7.07860	1.10550	40.09850
C	22.31370	-0.06940	35.97250	H	5.46880	1.04900	39.41550
O	23.46030	-0.42790	36.14100	H	5.83430	-0.00830	36.69810
C	20.13730	-0.80170	37.04780	H	6.02720	-1.74040	37.01070
C	18.78360	-1.52970	36.82100	H	4.79440	-0.84090	37.87250
O	18.60800	-2.02710	35.67290	H	6.69350	3.09510	38.67450
O	17.95100	-1.56460	37.77480	H	7.99710	2.10240	38.00870
N	18.10440	2.81680	45.89960	H	6.38780	2.08130	37.26370
C	16.91940	3.66180	45.71620	H	8.68050	-3.39970	39.72590
C	16.87140	4.65230	46.87650	H	6.57520	-5.30730	38.90920
O	15.85680	5.04060	47.41300	H	8.09840	-6.42310	37.34660
C	15.57830	2.93910	45.52420	H	9.43140	-5.32060	37.80060
C	15.56870	2.03750	44.28870	H	11.85470	2.53240	41.84010
C	14.24150	1.29560	44.10130	H	11.27640	0.08330	40.37450
C	14.33820	0.33120	42.92490	H	9.42660	1.62740	39.95580
N	13.03700	-0.36400	42.69560	H	10.30400	0.21840	38.31280
N	10.84740	-7.65870	44.83940	H	9.73430	3.71510	38.69440
C	11.89140	-8.59860	44.42690	H	10.59530	3.84530	40.23530
C	13.23500	-8.09740	44.94460	H	11.49880	3.54130	38.74300
O	14.30540	-8.32420	44.42330	H	12.92510	-0.32780	38.84900
C	11.96270	-8.99060	42.94260	H	15.19890	0.14710	38.02570
C	12.05090	-7.86170	41.90570	H	15.00620	1.80860	38.57990
O	11.73260	-6.66920	42.26870	H	12.06250	1.47030	37.22550
O	12.36970	-8.21530	40.74840	H	12.71570	1.43010	34.40160
N	17.38740	-4.17270	42.78760	H	10.39640	2.55430	34.23120
C	17.80110	-2.77480	42.76960	H	10.33630	2.51410	35.99340
C	17.55610	-2.17980	44.16760	H	10.69960	-0.03090	35.83340
O	17.29190	-1.01720	44.39830	H	10.44270	0.10820	34.09380
C	17.30320	-1.87910	41.59930	H	8.15330	1.21760	34.68790

H	8.50330	0.79660	36.35420	H	18.73440	-7.04810	32.40030
H	8.21080	-1.50460	35.90490	H	17.78050	-4.02600	35.30770
H	6.78700	-0.82020	32.22420	H	16.92240	-8.45680	33.27390
H	7.76640	0.41150	32.88050	H	15.96480	-5.42190	36.17420
H	6.63790	-2.87750	33.14910	H	15.50640	-7.66670	35.16840
H	6.93300	-3.17020	34.82320	H	22.31130	-2.58670	36.32040
H	9.82500	3.55100	29.53410	H	20.56520	-0.68880	34.95760
H	7.53870	1.72740	29.52750	H	19.90460	0.26060	37.20310
H	8.84830	0.20210	30.89380	H	20.58730	-1.16200	37.98030
H	10.32700	1.81420	32.02730	H	17.90290	2.08620	46.58070
H	11.21180	2.10830	30.53600	H	17.11670	4.28740	44.82870
H	10.30500	-0.66620	29.11550	H	14.77860	3.68620	45.46860
H	8.84390	0.00290	28.38640	H	15.37300	2.34320	46.42450
H	10.36640	0.91220	28.32960	H	16.37180	1.29530	44.36230
H	12.36310	0.44810	32.12510	H	15.77040	2.63930	43.39090
H	11.01110	-0.65820	31.85390	H	13.42450	2.01070	43.93910
H	12.07520	-0.21690	30.51940	H	14.00100	0.73440	45.01680
H	8.43280	-5.79880	27.90800	H	15.10140	-0.42510	43.09980
H	6.50780	-5.99970	30.09330	H	14.56480	0.87080	42.00100
H	6.99220	-3.63120	30.55610	H	12.28260	0.37790	42.45300
H	8.04540	-3.52220	29.16330	H	13.06170	-1.05930	41.93890
H	9.63200	-5.14790	30.20300	H	12.73530	-0.88750	43.51730
H	10.56050	-3.58770	31.91210	H	10.87610	-6.87590	44.18510
H	10.00930	-2.70820	30.47740	H	11.73580	-9.52780	45.00310
H	9.02740	-2.70120	31.94720	H	11.06590	-9.56560	42.67910
H	9.44170	-5.73580	32.61140	H	12.81770	-9.64990	42.77300
H	7.86340	-4.95820	32.70830	H	16.37260	-4.22830	42.76470
H	8.07590	-6.40740	31.71790	H	18.90270	-2.77270	42.71220
H	18.71780	-7.04630	39.33190	H	17.98830	-1.93970	40.74720
H	17.45700	-8.67410	40.62570	H	17.30410	-0.83340	41.93520
H	15.40120	-7.57930	40.87610	H	14.73130	-2.32780	39.44960
H	15.04170	-8.99970	39.92930	H	16.41930	-2.00560	39.15730
H	15.15290	-5.09420	39.65900	H	11.93420	-5.81060	38.77000
H	13.87310	-4.32440	37.63020	H	11.87150	-7.07680	39.74090
H	14.12580	-9.70980	37.58600	H	13.62880	-3.68650	41.46850
H	12.41960	-5.47240	35.51690	H	12.82370	-3.33840	40.20090
H	12.72790	-9.77470	35.54040	O	12.52730	-2.10260	38.89090
H	11.89620	-7.68450	34.50460	P	11.05080	-2.48110	38.69490
H	21.49610	-6.27890	31.30870	O	10.53570	-3.65080	39.54400
H	21.59560	-4.40110	33.02430	O	10.06810	-1.29090	38.61240
H	19.11450	-3.92600	32.38230	O	10.87120	-3.19870	37.21780
H	19.66580	-3.71940	34.01620	C	11.23470	-2.85890	35.87510

C	12.60020	-2.26350	35.58700
H	12.64360	-1.22060	35.92600
O	13.65840	-3.00330	36.18950
C	14.89910	-2.28000	36.07730
H	14.74470	-1.25290	36.44140
O	15.79020	-2.97870	36.87950
C	15.30920	-2.21630	34.59100
H	15.53610	-3.24900	34.28190
O	16.38950	-1.34390	34.38180
C	14.16500	-1.66340	33.71610
H	14.40520	-1.87040	32.66310
O	13.98870	-0.26990	33.92010
C	12.82190	-2.31830	34.05630
H	12.02750	-1.77390	33.53790
O	12.75590	-3.66040	33.57550
H	11.12280	-3.78870	35.31420
H	10.50330	-2.15000	35.46980
H	16.59910	-2.43200	37.07720
H	17.19200	-1.65860	34.86890
H	14.82520	0.10830	34.24810
H	13.40430	-4.17700	34.07990
H	9.40260	-3.28010	37.04470
H	9.26840	0.54600	44.47860
H	7.96580	-7.09320	39.86590
H	10.43910	1.70590	42.14000
H	12.68790	3.76630	33.82170
H	9.04640	2.92010	28.24700
H	7.50710	3.78280	31.17230
H	8.40850	-6.87040	29.13560
H	5.45340	-5.75900	27.74410
H	17.32620	-6.46790	38.68210
H	17.23170	-8.83120	37.55550
H	19.92950	-5.89220	31.15620
H	21.06970	-6.08280	34.85190
H	20.78450	-2.99650	35.82980
H	22.05280	1.01370	35.92180
H	18.33360	2.33570	45.03470
H	17.87840	4.99820	47.20160
H	9.93690	-8.09490	44.71760
H	13.15060	-7.50370	45.88280
H	17.73300	-4.68080	41.97660
H	17.67440	-2.92880	44.97890

WT-Glc6P-TS

N	8.89750	0.64950	43.89010
C	9.16350	-0.43640	42.94390
C	7.83710	-0.80390	42.25260
O	6.77090	-0.73190	42.86220
C	9.81690	-1.70340	43.54400
C	10.52010	-2.53690	42.46750
O	10.54570	-3.78330	42.58080
O	11.06540	-1.84880	41.53480
N	7.95530	-1.20520	40.96160
C	6.78850	-1.43320	40.11510
C	6.58410	-2.89490	39.70230
O	5.45560	-3.28930	39.40930
C	6.85000	-0.46910	38.88900
C	6.64070	0.97540	39.39300
C	5.84750	-0.83380	37.78620
C	7.03140	2.06420	38.38930
N	7.68540	-3.68700	39.64480
C	7.59390	-5.02070	39.11990
C	8.23510	-6.02790	40.02950
O	8.99780	-5.77280	40.94240
C	8.28660	-5.22150	37.72750
C	7.81200	-4.15180	36.75300
O	6.90680	-4.40360	35.93200
O	8.38380	-3.00390	36.87130
N	11.61820	1.70310	41.80460
C	11.64230	1.27060	40.39790
C	13.03550	1.57550	39.84620
O	13.62720	2.60310	40.19310
C	10.54880	1.92240	39.49890
O	10.50510	1.26260	38.23790
C	10.73550	3.40950	39.23470
N	13.51710	0.68830	38.94090
C	14.60290	1.03130	38.03960
C	14.14370	1.42000	36.62180
O	14.97970	1.60010	35.73800
N	12.80800	1.55640	36.42650
C	12.30690	2.04310	35.15490
C	12.71900	3.49910	34.90610
O	13.02160	4.28730	35.77020
C	10.76300	1.94290	35.11340

C	10.24030	0.49580	35.07010	C	18.11140	-6.60960	33.30120
C	8.73480	0.43340	35.37910	C	17.62900	-4.97870	35.00360
N	8.14320	-0.89110	35.17490	C	17.09430	-7.42540	33.79870
C	7.44860	-1.27860	34.11060	C	16.60310	-5.78710	35.49280
N	7.36050	-0.50280	33.00000	C	16.33160	-7.02040	34.89450
N	6.83160	-2.46450	34.09690	N	21.56100	-2.38970	35.80950
N	8.89940	3.08110	29.16580	C	21.11330	-1.00380	35.89930
C	8.38070	2.04210	30.05670	C	22.31370	-0.06940	35.97250
C	7.85410	2.73790	31.30290	O	23.45120	-0.42130	36.20020
O	7.75260	2.24150	32.40970	C	20.11120	-0.71730	37.05800
C	9.29310	0.81790	30.33920	C	18.76220	-1.46520	36.86260
C	10.58920	1.20990	31.08290	O	18.57800	-1.98990	35.73010
C	9.56840	0.06060	29.02930	O	17.94120	-1.48610	37.82810
C	11.48270	0.01290	31.42870	N	18.18140	2.85040	45.93170
N	7.71240	-6.43450	28.68580	C	16.98020	3.66330	45.71800
C	6.89960	-5.43760	29.39160	C	16.87140	4.65230	46.87650
C	5.82360	-4.95300	28.41500	O	15.82950	5.03980	47.35690
O	5.40760	-3.81600	28.35720	C	15.66510	2.90330	45.48330
C	7.66030	-4.27310	30.06050	C	15.70850	2.03850	44.22190
C	8.79620	-4.69800	31.01530	C	14.42460	1.23130	43.99810
C	9.58060	-3.47240	31.51060	C	14.56860	0.36170	42.75360
C	8.29550	-5.52920	32.20630	N	13.30170	-0.38260	42.46180
N	17.83680	-6.90020	39.40080	N	10.85030	-7.64040	44.87960
C	17.18070	-8.18350	39.68960	C	11.87980	-8.59000	44.45070
C	17.41230	-9.21820	38.58740	C	13.23500	-8.09740	44.94460
O	17.81430	-10.34640	38.78380	O	14.29310	-8.31010	44.39320
C	15.66810	-7.93850	39.97090	C	11.92400	-8.97630	42.96410
C	14.93020	-7.14560	38.92830	C	12.05000	-7.84030	41.93960
C	14.78350	-5.77890	38.90450	O	11.69660	-6.65490	42.29670
C	14.18410	-7.64470	37.79990	O	12.42810	-8.17840	40.79770
N	13.95840	-5.39340	37.86460	N	17.57890	-4.19780	42.81450
C	13.61320	-6.51970	37.13990	C	17.94160	-2.78570	42.81110
C	13.93770	-8.92890	37.28920	C	17.55610	-2.17980	44.16760
C	12.84360	-6.64690	35.98130	O	17.27660	-1.01330	44.35950
C	13.16260	-9.05980	36.14260	C	17.50060	-1.93150	41.59620
C	12.62750	-7.92970	35.49200	C	16.08290	-2.24100	41.12750
N	20.71050	-5.52460	31.46010	O	15.18430	-2.52390	41.95530
C	20.77740	-5.14100	32.87010	N	15.87210	-2.15530	39.81060
C	21.16910	-6.30470	33.76570	Mg	11.21350	-5.07050	41.11590
O	21.46640	-7.40930	33.36120	O	11.41810	-6.25770	39.42060
C	19.49790	-4.44890	33.40260	O	13.14260	-4.22350	40.98190
C	18.39610	-5.37260	33.89820	H	7.93230	0.54670	44.20340

H	9.85390	-0.08350	42.17860	H	7.45830	1.66480	29.58140
H	9.10060	-2.32340	44.08890	H	8.73080	0.14440	30.99840
H	10.58760	-1.39150	44.26550	H	10.31920	1.73190	32.00870
H	8.85680	-1.12740	40.49070	H	11.16470	1.92570	30.47700
H	5.90720	-1.19320	40.71430	H	10.06870	-0.89060	29.22080
H	7.86290	-0.56060	38.47660	H	8.63770	-0.16280	28.49500
H	7.22160	1.12520	40.31010	H	10.21420	0.64250	28.35950
H	5.58750	1.09390	39.68150	H	12.29630	0.28690	32.10690
H	5.88480	-0.09110	36.98240	H	10.89530	-0.77400	31.91520
H	6.06580	-1.80900	37.34840	H	11.93580	-0.42690	30.53540
H	4.82710	-0.87040	38.18000	H	8.40100	-5.96810	28.09800
H	6.85290	3.06110	38.80680	H	6.33400	-5.98940	30.16030
H	8.09340	1.99410	38.13530	H	6.93530	-3.64300	30.59520
H	6.45730	1.99420	37.45950	H	8.07360	-3.63480	29.26660
H	8.61900	-3.34650	39.84560	H	9.49590	-5.32140	30.43970
H	6.52840	-5.25440	39.01190	H	10.42300	-3.76880	32.14560
H	8.03030	-6.20940	37.33730	H	9.98350	-2.89270	30.67280
H	9.36980	-5.14860	37.85830	H	8.93590	-2.80490	32.09540
H	12.08470	2.60760	41.86710	H	9.13250	-5.83670	32.84110
H	11.44840	0.19920	40.37500	H	7.61170	-4.95320	32.84090
H	9.59590	1.76240	40.03050	H	7.77140	-6.43910	31.89180
H	10.37730	0.29640	38.42010	H	18.81160	-7.04820	39.14870
H	9.86360	3.79340	38.69780	H	17.62190	-8.60910	40.59840
H	10.83970	3.96770	40.16910	H	15.61310	-7.40490	40.92480
H	11.62580	3.58940	38.62580	H	15.16590	-8.89760	40.13320
H	13.06740	-0.22380	38.84710	H	15.16000	-5.04380	39.59650
H	15.32270	0.21230	37.95190	H	13.84920	-4.44140	37.52250
H	15.13320	1.89230	38.45330	H	14.35030	-9.80300	37.78480
H	12.14810	1.46430	37.19800	H	12.44170	-5.77700	35.47750
H	12.73480	1.43350	34.35190	H	12.96780	-10.04840	35.73640
H	10.38930	2.48940	34.23730	H	12.03750	-8.06090	34.58930
H	10.36420	2.45210	35.99780	H	21.02640	-6.49160	31.37440
H	10.76570	-0.11410	35.81010	H	21.59660	-4.41290	33.01270
H	10.44480	0.05260	34.08940	H	19.12320	-3.79980	32.60020
H	8.18660	1.17590	34.78920	H	19.77080	-3.76060	34.20600
H	8.57970	0.69680	36.42700	H	18.69890	-6.95330	32.45490
H	8.23890	-1.61910	35.91370	H	17.83190	-4.02490	35.47900
H	6.58800	-0.68400	32.37780	H	16.89840	-8.38490	33.32560
H	7.69720	0.45560	32.98080	H	16.01920	-5.43590	36.33600
H	6.47250	-2.79950	33.21660	H	15.53530	-7.65110	35.27610
H	6.85320	-3.16020	34.89430	H	22.31050	-2.54510	36.47940
H	9.82940	3.36710	29.46080	H	20.57680	-0.75040	34.97340

H	19.87210	0.35310	37.12600
H	20.54510	-1.00360	38.02330
H	17.98220	2.11180	46.60330
H	17.18400	4.29620	44.83840
H	14.84350	3.62700	45.43330
H	15.46470	2.27610	46.36290
H	16.54800	1.33790	44.27890
H	15.88320	2.67950	43.34620
H	13.56480	1.90400	43.89360
H	14.23360	0.59330	44.87270
H	15.36320	-0.36830	42.88450
H	14.77030	0.97690	41.87300
H	12.51910	0.34220	42.33870
H	13.36770	-0.97020	41.62170
H	13.05870	-1.02890	43.21180
H	10.86520	-6.86890	44.21130
H	11.72570	-9.51840	45.02870
H	11.00030	-9.51080	42.70590
H	12.75070	-9.66760	42.78130
H	16.56920	-4.28670	42.73390
H	19.04370	-2.74020	42.84040
H	18.19760	-2.05670	40.76170
H	17.53310	-0.87100	41.87850
H	14.95180	-2.33620	39.42330
H	16.62960	-1.96530	39.15260
H	11.95680	-5.81710	38.74820
H	11.93340	-7.05440	39.75900
H	13.76160	-3.91200	41.66260
H	13.10890	-3.46980	40.35080
O	12.64950	-2.09630	39.33350
P	11.14950	-2.34550	39.38470
O	10.57490	-3.70250	39.71140
O	10.18930	-1.22550	39.04250
O	10.87120	-3.19870	37.21780
C	11.21700	-3.16640	35.84450
C	12.51670	-2.44910	35.53250
H	12.45190	-1.39940	35.84690
O	13.61190	-3.07110	36.22010
C	14.80680	-2.28760	36.11350
H	14.58310	-1.25460	36.42410
O	15.70450	-2.90220	36.98290
C	15.27400	-2.26510	34.64620
H	15.54940	-3.29700	34.38040
O	16.33020	-1.35890	34.44640
C	14.13680	-1.78050	33.72820
H	14.42650	-1.97470	32.68550
O	13.89260	-0.39350	33.92990
C	12.82050	-2.51130	34.02400
H	12.01710	-2.02340	33.46490
O	12.86360	-3.85970	33.55860
H	11.26770	-4.18840	35.45330
H	10.43550	-2.65220	35.26590
H	16.52560	-2.35860	37.09470
H	17.14290	-1.65660	34.92440
H	14.72910	0.02440	34.20720
H	13.48910	-4.33700	34.12580
H	9.79440	-3.10370	37.22880
H	9.48950	0.55510	44.71050
H	8.00730	-7.08580	39.78170
H	10.67050	1.78910	42.17120
H	12.66960	3.80240	33.83310
H	8.99100	2.72310	28.22130
H	7.53740	3.78540	31.12460
H	8.23580	-6.99780	29.34990
H	5.44670	-5.75530	27.74370
H	17.38370	-6.46030	38.59960
H	17.19800	-8.84860	37.55560
H	19.74840	-5.49710	31.13370
H	21.21050	-6.05760	34.84920
H	20.78020	-2.99560	36.04080
H	22.06510	1.00680	35.82430
H	18.45150	2.38220	45.07180
H	17.85900	5.00050	47.25310
H	9.93540	-8.07520	44.78710
H	13.17220	-7.52070	45.89550
H	17.98270	-4.69060	42.02090
H	17.58870	-2.91820	44.99550

WT-Glc6P-P

N	9.02140	0.71980	43.94450
C	9.26570	-0.35090	42.98100
C	7.92580	-0.71840	42.31250
O	6.87410	-0.65790	42.94750
C	9.91280	-1.63090	43.56920

C	10.46030	-2.53260	42.47510	C	8.50930	2.23160	30.02570
O	10.34430	-3.76110	42.52620	C	7.85410	2.73790	31.30290
O	11.06540	-1.84880	41.53480	O	7.60840	2.07560	32.29340
N	8.02560	-1.12360	41.02270	C	9.40270	0.97620	30.21580
C	6.85160	-1.37520	40.19400	C	10.61330	1.24790	31.13430
C	6.65560	-2.84850	39.81410	C	9.81730	0.41640	28.84720
O	5.52110	-3.26410	39.58770	C	11.44870	-0.00150	31.43820
C	6.90010	-0.44050	38.94570	N	7.73080	-6.42890	28.49610
C	6.74060	1.02150	39.41710	C	6.96590	-5.46740	29.29700
C	5.85380	-0.80930	37.88690	C	5.82360	-4.95300	28.41500
C	7.14940	2.06860	38.37680	O	5.41350	-3.81260	28.41550
N	7.76850	-3.63250	39.72550	C	7.76860	-4.32590	29.95710
C	7.65770	-4.95060	39.15430	C	8.98590	-4.78080	30.78940
C	8.23510	-6.02790	40.02950	C	9.79840	-3.57070	31.27780
O	9.15860	-5.91270	40.81400	C	8.59520	-5.67810	31.97440
C	8.36880	-5.11960	37.77540	N	17.99850	-6.94710	39.46560
C	7.86660	-4.08250	36.77230	C	17.26950	-8.19630	39.71890
O	7.10670	-4.45180	35.84430	C	17.41230	-9.21820	38.58740
O	8.27370	-2.88420	36.95800	O	17.59640	-10.40460	38.76150
N	11.86000	1.71770	41.77800	C	15.77910	-7.86560	40.02500
C	11.82530	1.30470	40.36560	C	15.05460	-7.09200	38.95880
C	13.19010	1.60830	39.74920	C	14.88110	-5.72630	38.90680
O	13.78220	2.65430	40.03640	C	14.33340	-7.62550	37.82900
C	10.69380	1.94200	39.50400	N	14.07230	-5.37920	37.84320
O	10.61010	1.25620	38.25770	C	13.74080	-6.52430	37.14830
C	10.86300	3.42530	39.20960	C	14.11190	-8.92490	37.34340
N	13.65220	0.68960	38.86570	C	12.94700	-6.69060	36.00930
C	14.71350	1.00100	37.92420	C	13.32260	-9.09330	36.21230
C	14.20750	1.38670	36.52280	C	12.74670	-7.98750	35.55280
O	15.01070	1.56460	35.60820	N	20.68100	-5.81990	31.43820
N	12.86640	1.52420	36.37380	C	20.83080	-5.22160	32.75800
C	12.33000	2.03060	35.12720	C	21.16910	-6.30470	33.76570
C	12.71900	3.49910	34.90610	O	21.13680	-7.49380	33.53440
O	13.05690	4.26390	35.77770	C	19.62930	-4.36620	33.28730
C	10.78710	1.91040	35.11290	C	18.42170	-5.16640	33.72660
C	10.28030	0.45820	35.08060	C	17.69990	-5.95990	32.82360
C	8.75500	0.38840	35.27320	C	18.00670	-5.14170	35.06540
N	8.19600	-0.95120	35.08840	C	16.62260	-6.73340	33.25350
C	7.70080	-1.43420	33.95360	C	16.91620	-5.89920	35.49480
N	7.74900	-0.71930	32.79720	C	16.22960	-6.71400	34.59310
N	7.18240	-2.66380	33.91340	N	21.60190	-2.40390	35.82360
N	9.10010	3.38790	29.34870	C	21.13010	-1.02430	35.88470

C	22.31370	-0.06940	35.97250	H	5.69610	1.17900	39.71950
O	23.45100	-0.40140	36.23250	H	5.88510	-0.09400	37.05870
C	20.10450	-0.73750	37.02140	H	6.03670	-1.80110	37.47390
C	18.77010	-1.51230	36.82410	H	4.84580	-0.80180	38.31320
O	18.61740	-2.10630	35.72410	H	7.01340	3.08230	38.76970
O	17.92420	-1.47690	37.76960	H	8.20430	1.95330	38.10860
N	18.36450	3.16820	45.67880	H	6.55560	1.99340	37.45960
C	17.02870	3.77160	45.62920	H	8.70210	-3.24320	39.77790
C	16.87140	4.65230	46.87650	H	6.58830	-5.14660	39.02590
O	15.83170	4.82480	47.47260	H	8.16810	-6.12110	37.38550
C	15.84490	2.80650	45.41770	H	9.44530	-4.99300	37.91910
C	15.91470	2.06570	44.07300	H	12.34040	2.61560	41.83300
C	14.74990	1.08710	43.84900	H	11.63190	0.23670	40.33720
C	14.86360	0.41140	42.48020	H	9.76150	1.78560	40.07270
N	13.64630	-0.42240	42.18480	H	10.46710	0.29670	38.46710
N	10.85830	-7.61490	44.89600	H	9.96740	3.79880	38.70520
C	11.87300	-8.57710	44.45870	H	11.00580	3.99480	40.13240
C	13.23500	-8.09740	44.94460	H	11.72720	3.59980	38.56230
O	14.28230	-8.27600	44.36160	H	13.19850	-0.22550	38.81940
C	11.90750	-8.94940	42.96830	H	15.41050	0.16440	37.82000
C	12.12840	-7.80120	41.97490	H	15.27620	1.85570	38.30720
O	11.71590	-6.62850	42.31460	H	12.23070	1.43320	37.16510
O	12.63370	-8.10900	40.87460	H	12.75100	1.44880	34.29930
N	17.79720	-4.28370	42.97200	H	10.39750	2.45190	34.24010
C	18.10480	-2.85960	42.90720	H	10.39470	2.41580	36.00230
C	17.55610	-2.17980	44.16760	H	10.75170	-0.12020	35.87930
O	17.27490	-1.00070	44.25270	H	10.56100	-0.01550	34.13370
C	17.74130	-2.11450	41.60400	H	8.24680	1.09880	34.61070
C	16.30560	-2.37670	41.15330	H	8.50960	0.69390	36.29220
O	15.43000	-2.71640	41.98050	H	8.19770	-1.63200	35.88610
N	16.05430	-2.18310	39.85480	H	7.13400	-1.02690	32.05910
Mg	11.21350	-5.07050	41.11590	H	7.89560	0.28550	32.80070
O	11.62930	-6.21290	39.45430	H	6.84070	-3.00740	33.02950
O	13.03920	-4.00900	41.45500	H	7.11330	-3.32040	34.75240
H	8.06610	0.60930	44.28490	H	9.99090	3.63140	29.77550
H	9.94640	0.00630	42.20710	H	7.64080	1.92870	29.41430
H	9.21350	-2.19430	44.19130	H	8.78430	0.21770	30.70760
H	10.76320	-1.33550	44.20030	H	10.25090	1.66880	32.07960
H	8.91300	-1.02790	40.52490	H	11.25860	2.01320	30.67920
H	5.97250	-1.12930	40.79390	H	10.31270	-0.55170	28.95070
H	7.89670	-0.56940	38.50370	H	8.94740	0.27270	28.19530
H	7.34200	1.18010	40.31970	H	10.51520	1.09090	28.33690

H	12.23140	0.19700	32.17610	H	15.92450	2.80120	43.25560
H	10.80890	-0.79870	31.83260	H	13.79380	1.62200	43.91490
H	11.94290	-0.38850	30.54180	H	14.76040	0.32160	44.63530
H	8.37440	-5.93400	27.88150	H	15.73310	-0.23930	42.44970
H	6.45880	-6.05310	30.08020	H	14.92450	1.15550	41.68330
H	7.08630	-3.73400	30.58440	H	12.81120	0.22460	42.25750
H	8.10590	-3.64290	29.16500	H	13.64010	-0.83450	41.23740
H	9.63810	-5.36570	30.12470	H	13.57220	-1.22710	42.80980
H	10.68730	-3.88890	31.83340	H	10.87650	-6.84690	44.22390
H	10.13020	-2.94470	30.44090	H	11.70880	-9.50590	45.03270
H	9.20030	-2.94040	31.94540	H	10.94790	-9.40870	42.69580
H	9.48590	-6.00440	32.52030	H	12.68550	-9.69370	42.78250
H	7.96100	-5.14430	32.69180	H	16.79970	-4.41310	42.82170
H	8.05550	-6.57850	31.65830	H	19.19640	-2.75900	43.03280
H	18.94620	-7.14130	39.14990	H	18.43270	-2.38670	40.79950
H	17.68730	-8.67750	40.61110	H	17.85630	-1.03420	41.76100
H	15.77550	-7.28560	40.95350	H	15.12600	-2.35000	39.47840
H	15.23430	-8.78940	40.24270	H	16.78970	-1.95220	39.18200
H	15.25130	-4.96510	39.57750	H	12.10710	-5.75440	38.75060
H	13.91300	-4.43580	37.49730	H	12.17250	-6.98870	39.78230
H	14.55040	-9.78090	37.84850	H	13.92450	-4.17040	41.80460
H	12.51910	-5.83990	35.49440	H	13.17110	-3.30840	40.77990
H	13.14380	-10.09320	35.82730	O	12.73470	-1.96160	39.60190
H	12.13830	-8.14930	34.66740	P	11.23190	-2.24780	39.71030
H	20.24180	-6.73230	31.53170	O	10.73290	-3.67380	39.75540
H	21.69580	-4.53760	32.74990	O	10.26280	-1.19990	39.18840
H	19.35910	-3.67420	32.47830	O	10.87120	-3.19870	37.21780
H	19.97550	-3.73120	34.10700	C	11.17800	-3.26350	35.83930
H	17.98160	-5.97130	31.77520	C	12.46830	-2.53130	35.49640
H	18.50680	-4.47730	35.76120	H	12.37280	-1.47260	35.76770
H	16.08080	-7.34850	32.53930	O	13.56010	-3.09600	36.24180
H	16.58070	-5.83310	36.52600	C	14.75050	-2.31020	36.12590
H	15.39100	-7.31230	34.92890	H	14.50410	-1.26290	36.36100
H	22.34530	-2.53460	36.50580	O	15.62570	-2.85760	37.06150
H	20.60650	-0.79220	34.94580	C	15.25820	-2.37350	34.67500
H	19.84530	0.32930	37.06920	H	15.52280	-3.42290	34.46730
H	20.53060	-0.99940	37.99760	O	16.33750	-1.49830	34.45890
H	18.35180	2.35570	46.29270	C	14.14080	-1.90990	33.71950
H	17.04500	4.49390	44.79490	H	14.45360	-2.12620	32.68780
H	14.91080	3.37480	45.49800	O	13.89150	-0.51660	33.88580
H	15.83300	2.08180	46.24370	C	12.81390	-2.63510	34.00080
H	16.85100	1.50000	44.00920	H	12.02500	-2.15970	33.40910

O	12.86570	-3.99280	33.56210
H	11.25610	-4.30800	35.51630
H	10.38130	-2.80870	35.23300
H	16.47530	-2.34990	37.10110
H	17.15050	-1.82340	34.91840
H	14.72520	-0.08510	34.14810
H	13.52050	-4.44930	34.11210
H	9.92670	-2.94480	37.29400
H	9.64170	0.62950	44.74420
H	7.78710	-7.03160	39.87530
H	10.92370	1.82710	42.16650
H	12.61960	3.83430	33.84560
H	9.29340	3.16710	28.37690
H	7.57120	3.80950	31.25740
H	8.30250	-7.01370	29.09870
H	5.39280	-5.73590	27.75310
H	17.53400	-6.43500	38.71650
H	17.34050	-8.78480	37.56140
H	20.09160	-5.24060	30.84900
H	21.43800	-5.91840	34.77410
H	20.82690	-3.01670	36.05650
H	22.05210	1.00130	35.80650
H	18.63300	2.82610	44.76110
H	17.82240	5.13660	47.19080
H	9.93790	-8.03980	44.81260
H	13.18940	-7.56030	45.91920
H	18.27320	-4.79760	42.23380
H	17.46950	-2.86780	45.03440

WT-GlcNAc6P-R

N	9.32330	0.53420	42.98360
C	9.40880	-0.74020	42.25710
C	8.07480	-0.91810	41.51420
O	7.00200	-0.67330	42.06820
C	9.68340	-2.00970	43.12460
C	10.73810	-2.90290	42.46920
O	10.43180	-4.08730	42.15230
O	11.87530	-2.36460	42.27550
N	8.20870	-1.37680	40.25180
C	7.08660	-1.64050	39.36860
C	6.96530	-3.12000	38.99620
O	5.88360	-3.62250	38.70710
C	7.21180	-0.74320	38.09630
C	6.81540	0.69710	38.47860
C	6.40830	-1.26790	36.89790
C	7.23010	1.75770	37.45520
N	8.14290	-3.81050	38.92090
C	8.17570	-5.20850	38.51250
C	8.22810	-6.00180	39.81780
O	9.27100	-6.21530	40.40860
C	9.38020	-5.45390	37.57770
C	9.13440	-4.63600	36.32290
O	8.23970	-4.94650	35.53510
O	9.83140	-3.53440	36.10270
N	11.68600	1.66800	41.34370
C	11.81410	1.16390	39.96910
C	13.15540	1.65610	39.42360
O	13.52690	2.81370	39.63140
C	10.64950	1.56180	39.01620
O	10.73170	0.79230	37.81350
C	10.60930	3.03760	38.64820
N	13.85270	0.76290	38.67640
C	14.90860	1.22500	37.79170
C	14.36970	1.84240	36.49480
O	15.04630	2.61260	35.81930
N	13.10700	1.48050	36.12550
C	12.57300	2.09740	34.92060
C	12.79610	3.61310	34.96870
O	12.36600	4.31400	35.85910
C	11.06590	1.80590	34.78780
C	10.74430	0.32070	34.55940
C	9.26130	0.03140	34.79800
N	8.92400	-1.35910	34.46720
C	8.02600	-1.78700	33.58970
N	7.41470	-0.95890	32.72110
N	7.72140	-3.09620	33.54700
N	9.24620	3.13320	29.32320
C	8.24220	2.23250	29.89120
C	8.05100	2.63410	31.34410
O	7.75350	1.87790	32.25270
C	8.43960	0.70850	29.68770
C	9.77150	0.23010	30.29430
C	8.30870	0.35170	28.20110
C	9.98660	-1.28390	30.26160

N	7.64100	-6.90360	28.71800
C	7.18470	-5.87790	29.65950
C	6.04890	-5.09590	28.99840
O	5.74850	-3.95730	29.28980
C	8.27250	-4.95140	30.24190
C	9.40670	-5.67840	30.99360
C	10.48460	-4.67950	31.44430
C	8.90230	-6.48990	32.19680
N	18.21230	-7.31680	39.93180
C	17.18040	-8.35040	39.77110
C	17.49590	-9.31510	38.62500
O	17.36020	-10.51890	38.68760
C	15.79470	-7.67620	39.56610
C	15.74580	-6.80900	38.34040
C	16.04990	-5.46540	38.26700
C	15.51500	-7.23490	36.98080
N	16.05620	-5.03860	36.95510
C	15.70080	-6.09720	36.14440
C	15.16450	-8.46500	36.40100
C	15.52020	-6.16660	34.75660
C	14.98960	-8.53440	35.02580
C	15.16250	-7.39550	34.21460
N	21.08170	-6.59470	31.33260
C	21.62360	-5.78720	32.41440
C	21.13440	-6.29910	33.75500
O	20.27150	-7.13620	33.89870
C	21.32280	-4.25690	32.31920
C	19.84350	-3.95960	32.28830
C	19.17350	-3.72830	31.08260
C	19.09740	-3.95300	33.47380
C	17.79670	-3.50020	31.06260
C	17.73100	-3.69740	33.46140
C	17.06730	-3.48300	32.25110
N	20.97640	-1.67030	34.74440
C	20.90970	-0.47820	35.57390
C	22.30650	-0.05740	35.99380
O	23.29080	-0.76100	35.92340
C	19.99180	-0.59560	36.82780
C	18.50780	-0.86640	36.47430
O	18.19070	-0.83110	35.25600
O	17.71230	-1.09240	37.43900
N	18.07980	2.67370	46.18560
C	17.00960	3.59070	45.77600
C	16.87040	4.65130	46.87760
O	15.82670	5.15840	47.22340
C	15.66930	2.94230	45.36910
C	15.81310	2.02450	44.14310
C	14.52300	1.28700	43.74260
C	14.77510	0.36220	42.54520
N	13.51950	-0.28150	42.05780
N	10.85310	-7.54490	44.83040
C	11.83990	-8.58990	44.53830
C	13.21740	-8.04390	44.88910
O	14.09300	-8.69750	45.41450
C	11.85780	-9.14220	43.08490
C	12.15430	-8.11110	41.98960
O	11.55520	-6.98480	42.09210
O	12.91900	-8.45080	41.05370
N	17.76840	-4.23620	42.87400
C	18.09150	-2.81500	42.85780
C	17.55880	-2.18080	44.15130
O	17.36490	-0.99210	44.30680
C	17.74450	-2.00090	41.58230
C	16.33840	-2.28370	41.05440
O	15.46610	-2.73570	41.82230
N	16.09780	-2.01500	39.76210
Mg	11.59570	-5.32560	40.93100
O	12.47090	-6.51750	39.42380
O	13.31520	-4.48580	41.77500
H	8.37020	0.62310	43.33590
H	10.22310	-0.66580	41.53200
H	8.76770	-2.56890	43.32900
H	10.10180	-1.68920	44.08640
H	9.15540	-1.44050	39.84920
H	6.16780	-1.39580	39.90660
H	8.27630	-0.75290	37.82650
H	7.27060	0.94160	39.44520
H	5.72840	0.72980	38.63830
H	6.47430	-0.56910	36.05860
H	6.78230	-2.23560	36.55210
H	5.35380	-1.40180	37.15850
H	6.96320	2.76110	37.80380
H	8.31240	1.73430	37.30010
H	6.74530	1.61000	36.48390

H	9.00620	-3.41800	39.28550	H	9.87650	-6.37660	30.28500
H	7.23620	-5.41070	37.99230	H	11.31960	-5.21080	31.91560
H	9.41430	-6.50920	37.29380	H	10.88300	-4.10080	30.60490
H	10.31090	-5.18780	38.08380	H	10.08080	-3.97550	32.18020
H	11.97530	2.64470	41.35620	H	9.73340	-7.00560	32.68860
H	11.80380	0.07440	39.99990	H	8.43800	-5.84420	32.95010
H	9.72500	1.29090	39.55250	H	8.17210	-7.25520	31.91120
H	10.66900	-0.15810	38.09770	H	19.13010	-7.74510	40.03200
H	9.68450	3.26490	38.11190	H	17.13210	-8.95010	40.68640
H	10.64280	3.65770	39.54830	H	15.59260	-7.07990	40.46180
H	11.45220	3.31410	38.01110	H	15.02420	-8.45250	39.53730
H	13.55970	-0.22040	38.67770	H	16.27790	-4.76730	39.06160
H	15.57400	0.39880	37.52980	H	16.02250	-4.04170	36.71020
H	15.49970	1.99830	38.28550	H	15.03520	-9.34620	37.02070
H	12.44430	1.11770	36.81030	H	15.67210	-5.30510	34.11740
H	13.11500	1.71300	34.04850	H	14.72090	-9.48100	34.56540
H	10.66470	2.39010	33.94930	H	15.02940	-7.48000	33.13930
H	10.57780	2.17050	35.69660	H	20.22000	-7.02830	31.65890
H	11.32270	-0.31070	35.24240	H	22.72360	-5.88070	32.44890
H	11.02460	0.04080	33.53980	H	21.80280	-3.88670	31.40590
H	8.62760	0.70950	34.22290	H	21.79730	-3.72130	33.15030
H	9.02850	0.19100	35.85350	H	19.73800	-3.71390	30.15240
H	9.37620	-2.08850	35.02360	H	19.59090	-4.13310	34.42410
H	6.65970	-1.31790	32.16010	H	17.29190	-3.33070	30.11470
H	7.67920	0.00990	32.55900	H	17.19420	-3.65960	34.40260
H	7.08640	-3.43560	32.84210	H	15.99490	-3.31480	32.23540
H	7.96530	-3.74720	34.31530	H	21.34920	-2.43950	35.29590
H	10.17700	2.86600	29.63430	H	20.49430	0.33410	34.96220
H	7.27850	2.51490	29.43060	H	20.01430	0.32190	37.43130
H	7.61850	0.21330	30.22310	H	20.34610	-1.40220	37.48180
H	9.84570	0.56430	31.33350	H	17.71260	2.00120	46.85690
H	10.61350	0.70970	29.78290	H	17.40000	4.15620	44.91220
H	8.33060	-0.73000	28.04960	H	14.93490	3.73400	45.18070
H	7.36730	0.72390	27.78060	H	15.28690	2.36880	46.22530
H	9.13340	0.78200	27.62030	H	16.58530	1.27310	44.33580
H	10.91920	-1.52300	30.77710	H	16.15890	2.62240	43.28730
H	9.17000	-1.81470	30.76220	H	13.73520	2.00800	43.49050
H	10.05850	-1.67120	29.23990	H	14.15710	0.69290	44.59100
H	8.28130	-6.49240	28.04110	H	15.48240	-0.42400	42.80380
H	6.68690	-6.41140	30.48470	H	15.17250	0.93370	41.70050
H	7.80500	-4.21200	30.90390	H	12.77650	0.47780	41.91230
H	8.70360	-4.36970	29.41490	H	13.64310	-0.79580	41.17050

H	13.10190	-1.00090	42.66660
H	10.87050	-6.88260	44.05290
H	11.67290	-9.43790	45.21270
H	10.86260	-9.55470	42.87030
H	12.58110	-9.95790	42.99880
H	16.77160	-4.34740	42.70250
H	19.18380	-2.72910	42.98870
H	18.47920	-2.19790	40.79370
H	17.81210	-0.93050	41.81690
H	15.14170	-2.12910	39.41520
H	16.79810	-1.62660	39.13090
H	13.25500	-6.14530	38.99750
H	12.76010	-7.33850	39.94250
H	14.14570	-4.15910	41.39460
H	12.88150	-3.62380	42.03830
C	14.39950	0.68420	30.28700
C	13.66300	0.12580	31.49400
O	12.45060	0.31750	31.64840
N	14.40030	-0.62360	32.34250
C	13.86580	-1.13500	33.59410
H	13.22130	-0.37430	34.03120
C	15.01420	-1.37890	34.57410
H	15.69420	-2.13140	34.15790
O	15.68560	-0.16120	34.76110
C	14.45410	-1.93920	35.88130
H	13.83800	-1.16730	36.35960
O	15.49680	-2.36400	36.74860
C	13.58260	-3.16780	35.59010
H	14.21340	-3.95010	35.15750
C	12.91750	-3.72830	36.82750
O	11.91280	-2.79580	37.26680
P	11.80570	-2.52760	38.95020
O	10.58570	-1.59060	38.96390
O	11.49250	-3.92880	39.47220
O	13.14430	-1.90080	39.34730
O	12.53020	-2.84510	34.65960
C	12.99310	-2.38390	33.41080
H	12.08480	-2.13390	32.85020
O	13.74800	-3.37130	32.73090
H	15.48020	0.75730	30.43470
H	13.99640	1.67430	30.06520
H	14.20740	0.03880	29.42300
H	15.40820	-0.60950	32.26460
H	16.64160	-0.36370	34.94460
H	16.25470	-1.71750	36.83370
H	12.44500	-4.69290	36.61010
H	13.66790	-3.87500	37.60590
H	13.22020	-4.18210	32.73540
H	9.94650	0.51780	43.78680
H	7.25410	-6.29620	40.25380
H	10.73450	1.57820	41.71680
H	13.30670	4.05160	34.08470
H	9.24340	3.07540	28.30980
H	8.18930	3.71680	31.53360
H	8.16470	-7.62390	29.20640
H	5.49600	-5.67500	28.22680
H	18.24110	-6.73850	39.09310
H	17.88710	-8.82620	37.70400
H	20.83670	-6.00690	30.54170
H	21.63120	-5.82740	34.63320
H	20.02420	-1.90010	34.47490
H	22.36790	0.96810	36.42970
H	18.39790	2.12570	45.39140
H	17.84370	4.92380	47.34310
H	9.92340	-7.95870	44.81670
H	13.37480	-6.97840	44.60710
H	18.25540	-4.74410	42.13970
H	17.40610	-2.91480	44.96950
H	10.63310	-3.28270	36.70740

WT-GlcNAc6P-TS

N	9.12590	-0.42370	44.79870
C	9.57320	-1.22320	43.65810
C	8.30500	-1.54760	42.83710
O	7.25990	-1.84360	43.41300
C	10.30520	-2.53880	44.04690
C	11.04280	-3.14590	42.86300
O	10.79520	-4.33010	42.52830
O	11.87530	-2.36460	42.27550
N	8.43020	-1.47340	41.49010
C	7.28850	-1.64310	40.59570
C	7.11860	-3.07650	40.07690
O	6.04310	-3.66290	40.15110
C	7.37960	-0.63750	39.41210

C	7.20700	0.79740	39.94720	C	6.83460	-5.74030	30.14450
C	6.36650	-0.97260	38.30490	C	6.04890	-5.09590	28.99840
C	7.43100	1.88950	38.89640	O	5.97100	-3.90340	28.79160
N	8.19410	-3.60690	39.42370	C	7.77610	-4.75490	30.86900
C	8.05650	-4.90630	38.78350	C	8.65880	-5.38500	31.96940
C	8.22810	-6.00170	39.81780	C	9.63990	-4.35160	32.54780
O	9.25560	-6.13030	40.47340	C	7.83540	-6.02160	33.09920
C	9.07410	-5.03980	37.63700	N	17.94470	-7.42040	40.18620
C	8.68790	-4.03390	36.54610	C	17.01500	-8.50140	39.83060
O	7.50540	-4.11500	36.10870	C	17.49590	-9.31510	38.62500
O	9.54530	-3.18110	36.16810	O	17.37000	-10.51660	38.51820
N	12.08820	1.80620	41.66280	C	15.60010	-7.91390	39.56140
C	12.16230	1.34440	40.27010	C	15.59880	-6.97230	38.39480
C	13.49410	1.78560	39.67740	C	15.81130	-5.61070	38.44290
O	13.98300	2.88110	39.96250	C	15.57830	-7.32220	36.99510
C	10.97230	1.78800	39.36970	N	15.95650	-5.09860	37.17160
O	10.90920	0.95150	38.21080	C	15.79750	-6.12180	36.25940
C	11.01230	3.23830	38.91210	C	15.39420	-8.53120	36.30240
N	14.05360	0.90840	38.80430	C	15.81870	-6.11100	34.85740
C	15.03780	1.34610	37.82500	C	15.41990	-8.51920	34.91460
C	14.40290	1.93150	36.55680	C	15.62820	-7.32090	34.20240
O	15.04180	2.66630	35.80800	N	22.53760	-5.05970	32.10830
N	13.11290	1.58460	36.29790	C	22.18910	-5.21840	33.51720
C	12.51970	2.11070	35.07970	C	21.13440	-6.29910	33.75500
C	12.79610	3.61310	34.96870	O	20.71730	-7.02590	32.88000
O	12.46370	4.40710	35.82240	C	21.72990	-3.88410	34.16090
C	10.99620	1.90320	35.07310	C	20.33420	-3.48970	33.72550
C	10.53290	0.44280	34.97900	C	20.08540	-3.00710	32.43170
C	9.04850	0.35420	35.35210	C	19.24800	-3.64390	34.59570
N	8.47310	-0.94420	35.00700	C	18.78320	-2.73450	32.01100
C	7.36680	-1.15040	34.30610	C	17.95290	-3.33170	34.18950
N	6.72710	-0.12920	33.67380	C	17.70880	-2.90330	32.88440
N	6.86030	-2.38780	34.24270	N	20.55390	-0.05880	34.31000
N	8.08760	2.64520	28.92740	C	20.81310	0.12080	35.72730
C	7.55400	1.93040	30.08970	C	22.30650	-0.05740	35.99380
C	8.05100	2.63410	31.34410	O	23.05260	-0.70800	35.29150
O	7.99390	2.17290	32.46980	C	20.02060	-0.81750	36.70770
C	7.77040	0.39350	30.14890	C	18.47540	-0.77080	36.56710
C	9.26920	0.03590	30.13160	O	18.00530	-0.24600	35.52430
C	6.97780	-0.30980	29.03660	O	17.78470	-1.31000	37.48950
C	9.54690	-1.44310	30.39700	N	18.45680	3.29950	45.64560
N	7.45070	-6.95880	29.60270	C	17.08620	3.81870	45.61480

C	16.87040	4.65130	46.87760	H	7.04690	-4.96050	38.37230
O	15.81590	4.75550	47.46530	H	9.01240	-6.04750	37.21200
C	15.96420	2.79170	45.40070	H	10.08970	-4.88040	38.00250
C	16.05710	2.07780	44.04900	H	12.50490	2.73530	41.71390
C	14.92430	1.07010	43.84100	H	12.10660	0.26210	40.27650
C	15.02470	0.39390	42.47730	H	10.06960	1.62040	39.97830
N	13.80480	-0.41640	42.20810	H	10.79840	0.03350	38.53670
N	10.84830	-8.26330	44.45030	H	10.05830	3.50890	38.45070
C	12.14720	-8.89650	44.21610	H	11.18830	3.90290	39.76410
C	13.21740	-8.04390	44.88910	H	11.80280	3.40890	38.17630
O	14.34010	-7.86900	44.46450	H	13.64880	-0.01810	38.72750
C	12.51160	-9.27120	42.77220	H	15.67950	0.51150	37.53230
C	12.34420	-8.18610	41.70220	H	15.65970	2.12480	38.26790
O	12.00300	-7.00500	42.07100	H	12.49720	1.21830	37.02010
O	12.55730	-8.55150	40.52260	H	12.98870	1.62050	34.21810
N	17.63150	-4.37590	43.10560	H	10.56860	2.45480	34.22520
C	18.06330	-2.99230	42.95050	H	10.60460	2.37190	35.98250
C	17.55880	-2.18080	44.15130	H	11.10400	-0.20620	35.65240
O	17.40910	-0.97560	44.15980	H	10.68780	0.06990	33.96180
C	17.79280	-2.30400	41.59330	H	8.49110	1.15010	34.85380
C	16.35900	-2.48260	41.09530	H	8.93920	0.51310	36.43030
O	15.44640	-2.78830	41.89950	H	8.91950	-1.79400	35.41930
N	16.15210	-2.27540	39.79040	H	5.92830	-0.38120	33.11300
Mg	11.59570	-5.32560	40.93100	H	7.25610	0.66470	33.31680
O	12.13580	-6.38360	39.20140	H	6.02960	-2.54310	33.69390
O	13.67920	-4.89820	41.34090	H	7.13180	-3.10710	34.98700
H	8.26370	-0.84440	45.14220	H	9.06980	2.40720	28.79450
H	10.26810	-0.62960	43.05200	H	6.46580	2.11980	30.10120
H	9.60250	-3.26060	44.47180	H	7.36590	0.06130	31.11230
H	11.05830	-2.29230	44.80850	H	9.81010	0.61660	30.88670
H	9.33810	-1.28510	41.06370	H	9.69900	0.31290	29.15730
H	6.38580	-1.45650	41.18250	H	6.99180	-1.39610	29.15180
H	8.38840	-0.72850	38.98600	H	5.92430	0.00310	29.03570
H	7.90430	0.94440	40.78090	H	7.39470	-0.07520	28.04800
H	6.20050	0.89650	40.37750	H	10.62010	-1.60930	30.49850
H	6.42000	-0.23610	37.49780	H	9.07960	-1.75890	31.33380
H	6.56100	-1.95150	37.85900	H	9.16260	-2.08490	29.59790
H	5.34340	-0.97540	38.69880	H	8.30400	-6.71500	29.10330
H	7.45860	2.88110	39.36070	H	6.05240	-6.07780	30.84320
H	8.37900	1.73930	38.36920	H	7.17810	-3.93980	31.29720
H	6.63380	1.90340	38.14610	H	8.42210	-4.28450	30.11580
H	9.12360	-3.21500	39.52660	H	9.25620	-6.17940	31.49650

H	10.29840	-4.79650	33.30080	H	10.77210	-7.47140	43.81220
H	10.26830	-3.91420	31.76400	H	12.16190	-9.82710	44.81090
H	9.10350	-3.53770	33.04570	H	11.91110	-10.12600	42.44250
H	8.49350	-6.50590	33.82740	H	13.55900	-9.59370	42.72990
H	7.26530	-5.26580	33.64880	H	16.63690	-4.43260	42.90410
H	7.13530	-6.78270	32.73380	H	19.15650	-2.97530	43.09560
H	18.88990	-7.78530	40.28660	H	18.48520	-2.68440	40.83490
H	16.94060	-9.19760	40.67350	H	17.99240	-1.22870	41.68640
H	15.28770	-7.38900	40.46850	H	15.20040	-2.31930	39.43070
H	14.89010	-8.73320	39.41690	H	16.89090	-1.99190	39.14570
H	15.86610	-4.96090	39.30500	H	12.97890	-6.05510	38.85350
H	15.92850	-4.09220	36.97140	H	12.33340	-7.30400	39.57320
H	15.23860	-9.45770	36.84720	H	14.10060	-4.02700	41.49860
H	15.98610	-5.19940	34.29550	H	13.90260	-5.46620	42.09120
H	15.28100	-9.44680	34.36660	C	13.71920	1.29340	30.89110
H	15.65150	-7.34230	33.11600	C	13.18720	0.23250	31.84020
H	22.83810	-5.96850	31.75680	O	11.98590	-0.04990	31.87820
H	23.09320	-5.54390	34.05720	N	14.10580	-0.35680	32.64350
H	22.46240	-3.11230	33.90840	C	13.70150	-1.15210	33.79290
H	21.75110	-3.99510	35.25060	H	12.92170	-0.61670	34.33890
H	20.92180	-2.82900	31.76110	C	14.90370	-1.33600	34.72340
H	19.41640	-3.99300	35.61150	H	15.62850	-1.99430	34.23820
H	18.60950	-2.37420	31.00010	O	15.45990	-0.06950	34.96680
H	17.14430	-3.39800	34.90780	C	14.40150	-2.01730	35.99320
H	16.69380	-2.69720	32.55800	H	13.73630	-1.31560	36.51180
H	20.98530	-0.92360	33.99890	O	15.46870	-2.41030	36.85830
H	20.55600	1.15420	36.00910	C	13.61340	-3.30240	35.66560
H	20.27140	-0.60880	37.75560	H	14.31980	-4.06590	35.33220
H	20.31380	-1.85630	36.51740	C	12.83610	-3.78790	36.88150
H	18.49720	2.47870	46.24720	O	11.91280	-2.79580	37.26680
H	17.05060	4.55690	44.79570	P	11.81570	-2.32260	39.93140
H	15.00000	3.30310	45.50080	O	10.71110	-1.29830	39.81130
H	16.00380	2.05560	46.21560	O	11.39350	-3.75560	39.79660
H	17.01210	1.54850	43.96850	O	13.25420	-1.86890	39.86300
H	16.02950	2.82140	43.24040	O	12.62290	-3.10680	34.63870
H	13.95520	1.57940	43.92120	C	13.10030	-2.51840	33.45300
H	14.96450	0.30580	44.62720	H	12.21920	-2.40640	32.81320
H	15.89100	-0.25920	42.43270	O	14.09910	-3.30830	32.82150
H	15.08530	1.13530	41.67810	H	14.79320	1.20370	30.70540
H	12.99270	0.24490	42.22380	H	13.52310	2.28270	31.32140
H	13.80570	-0.91000	41.28900	H	13.17410	1.22090	29.94690
H	13.63840	-1.17530	42.87470	H	15.00280	0.10010	32.76930

H	16.43160	-0.14640	35.18910
H	16.23550	-1.77560	36.90970
H	12.33820	-4.73880	36.65050
H	13.52930	-3.95790	37.71020
H	13.73170	-4.19910	32.73490
H	9.81100	-0.47030	45.54860
H	7.39160	-6.70880	39.96890
H	11.12090	1.87010	41.97180
H	13.23650	3.94970	34.00440
H	7.60600	2.34950	28.08420
H	8.46340	3.64840	31.17010
H	7.73440	-7.57140	30.36120
H	5.53970	-5.83830	28.34450
H	17.96490	-6.74020	39.42720
H	18.00140	-8.71310	37.83540
H	21.67430	-4.87850	31.59740
H	20.77430	-6.39480	34.80490
H	19.54730	-0.15550	34.19900
H	22.68050	0.40970	36.93470
H	18.73250	2.98380	44.72060
H	17.79050	5.17550	47.21990
H	10.10360	-8.91060	44.20350
H	12.87220	-7.58410	45.84240
H	18.09910	-4.99250	42.44580
H	17.37430	-2.80340	45.05200
H	11.05260	-2.96070	36.82930

WT-GlcNAc6P-P

N	9.45680	-0.17730	44.54630
C	9.70900	-1.03090	43.39170
C	8.34980	-1.26530	42.69370
O	7.32120	-1.35770	43.36230
C	10.35290	-2.40540	43.73830
C	10.86440	-3.14850	42.51960
O	10.62860	-4.34730	42.34140
O	11.58360	-2.38060	41.72600
N	8.40410	-1.37160	41.34690
C	7.22580	-1.66800	40.53470
C	7.17110	-3.14880	40.13190
O	6.20970	-3.86560	40.40470
C	7.18560	-0.73690	39.29090
C	6.89880	0.70750	39.74150
C	6.17330	-1.23040	38.24550
C	7.05190	1.74540	38.62560
N	8.21240	-3.60220	39.38720
C	8.08700	-4.91310	38.76610
C	8.22810	-6.00180	39.81780
O	9.25420	-6.17900	40.46150
C	9.11940	-5.04120	37.63100
C	8.71450	-4.04100	36.53750
O	7.57840	-4.21460	36.01520
O	9.50810	-3.09080	36.25930
N	11.81040	1.59660	41.52680
C	11.91160	1.08890	40.14850
C	13.24400	1.55680	39.56680
O	13.70110	2.66640	39.86210
C	10.73200	1.46270	39.20790
O	10.75880	0.61610	38.06040
C	10.72220	2.91390	38.74880
N	13.83750	0.69770	38.70310
C	14.88140	1.15000	37.79610
C	14.33020	1.80300	36.52080
O	15.02180	2.54680	35.83200
N	13.04250	1.50840	36.19420
C	12.49320	2.11050	34.98980
C	12.79610	3.61310	34.96870
O	12.44540	4.36650	35.85100
C	10.96990	1.91230	34.94130
C	10.50470	0.45290	34.80630
C	9.04340	0.35410	35.25280
N	8.45470	-0.95000	34.95050
C	7.32820	-1.16000	34.29030
N	6.65230	-0.14020	33.68720
N	6.83090	-2.40520	34.22490
N	8.12020	2.57660	28.92630
C	7.58990	1.88380	30.10270
C	8.05100	2.63410	31.34410
O	7.94950	2.21090	32.48220
C	7.85460	0.35680	30.21250
C	9.36400	0.04810	30.16780
C	7.05830	-0.41310	29.14950
C	9.70840	-1.37580	30.60480
N	7.44190	-6.98130	29.55650
C	6.85930	-5.75580	30.11810

C	6.04890	-5.09590	28.99840	O	15.83950	4.74980	47.50480
O	5.96790	-3.90050	28.80940	C	15.88650	2.82980	45.35690
C	7.83330	-4.78510	30.81790	C	15.97390	2.11650	43.99660
C	8.72760	-5.43090	31.89990	C	14.82200	1.12700	43.75860
C	9.72260	-4.40740	32.47260	C	14.91540	0.45520	42.38490
C	7.91690	-6.06740	33.03910	N	13.71470	-0.40720	42.14110
N	17.92250	-7.33510	40.09210	N	10.85600	-8.16940	44.76030
C	17.01980	-8.45860	39.80300	C	12.06570	-8.93080	44.42970
C	17.49590	-9.31510	38.62500	C	13.21740	-8.04390	44.88910
O	17.40370	-10.52430	38.58450	O	14.05030	-7.54490	44.16160
C	15.58160	-7.92480	39.54290	C	12.21520	-9.42870	42.98570
C	15.51750	-6.99330	38.36780	C	12.19730	-8.37870	41.86940
C	15.63980	-5.61870	38.41270	O	11.60430	-7.26310	42.10110
C	15.47480	-7.34060	36.96750	O	12.72440	-8.71690	40.78610
N	15.69520	-5.09630	37.14060	N	17.51150	-4.42690	43.21160
C	15.58060	-6.12450	36.23210	C	17.94370	-3.05740	42.95230
C	15.35020	-8.55420	36.26870	C	17.55880	-2.18080	44.15130
C	15.54980	-6.10160	34.83220	O	17.31010	-0.99310	44.09530
C	15.32560	-8.53120	34.88110	C	17.57390	-2.43960	41.58590
C	15.42290	-7.31670	34.17210	C	16.11050	-2.62260	41.17810
N	21.09000	-6.56050	31.26970	O	15.23950	-2.94660	42.01980
C	21.59270	-5.77980	32.39340	N	15.82950	-2.39690	39.88860
C	21.13440	-6.29910	33.75500	Mg	11.45780	-5.63350	40.86840
O	20.53840	-7.34140	33.91230	O	12.13430	-6.73570	39.24740
C	21.26430	-4.27510	32.23270	O	13.46590	-5.24310	41.63370
C	19.77560	-4.00380	32.25190	H	8.56110	-0.45820	44.94350
C	19.04080	-3.93920	31.06170	H	10.39030	-0.51990	42.70400
C	19.09030	-3.85950	33.46550	H	9.65160	-3.04330	44.28100
C	17.65970	-3.73890	31.08760	H	11.21970	-2.22200	44.38870
C	17.71700	-3.63300	33.49360	H	9.30230	-1.35640	40.84930
C	16.98990	-3.58430	32.30190	H	6.34740	-1.50710	41.16260
N	20.94870	-1.36950	34.45860	H	8.18430	-0.76290	38.83350
C	20.89000	-0.37720	35.52020	H	7.57970	0.95700	40.56440
C	22.30650	-0.05740	35.99380	H	5.88470	0.75630	40.16270
O	23.27550	-0.75600	35.78670	H	6.12550	-0.53880	37.39900
C	19.98000	-0.75160	36.73850	H	6.44900	-2.20930	37.84350
C	18.47750	-0.96020	36.37680	H	5.16940	-1.30930	38.67930
O	18.15940	-0.86250	35.16130	H	6.98690	2.76460	39.02190
O	17.67980	-1.21890	37.33110	H	8.02140	1.63960	38.12960
N	18.39570	3.26270	45.60950	H	6.27750	1.64140	37.85880
C	17.04020	3.82460	45.59410	H	9.05190	-3.03760	39.24140
C	16.87040	4.65130	46.87760	H	7.08300	-4.99220	38.33930

H	9.08220	-6.05210	37.21290	H	10.34860	-3.96890	31.68710
H	10.12450	-4.84960	38.01180	H	9.19710	-3.59190	32.98070
H	12.18920	2.54370	41.54260	H	8.58170	-6.56290	33.75370
H	11.89300	0.00690	40.18940	H	7.36290	-5.31020	33.60310
H	9.81870	1.25490	39.78960	H	7.20560	-6.82050	32.67940
H	10.69940	-0.31400	38.38510	H	18.88450	-7.66250	40.15290
H	9.79850	3.13050	38.20620	H	16.98190	-9.11980	40.67630
H	10.77840	3.58650	39.61060	H	15.25240	-7.40630	40.44910
H	11.56390	3.13780	38.08870	H	14.90790	-8.77720	39.41790
H	13.47180	-0.25240	38.64470	H	15.68580	-4.96770	39.27410
H	15.52380	0.31390	37.50780	H	15.68730	-4.09070	36.92580
H	15.49670	1.89950	38.29560	H	15.27780	-9.49330	36.80760
H	12.41110	1.07010	36.86090	H	15.63320	-5.17530	34.27730
H	12.96940	1.66500	34.10930	H	15.23290	-9.46310	34.33040
H	10.56100	2.48720	34.10110	H	15.40830	-7.32820	33.08560
H	10.56190	2.36070	35.85410	H	21.15250	-7.54650	31.52030
H	11.10170	-0.22650	35.42460	H	22.69350	-5.86560	32.41700
H	10.61740	0.12670	33.76820	H	21.70070	-3.94240	31.28630
H	8.45460	1.14690	34.78680	H	21.75970	-3.70500	33.02620
H	8.99970	0.50790	36.33570	H	19.55960	-4.04540	30.11190
H	8.88820	-1.78620	35.41090	H	19.63920	-3.89350	34.40210
H	5.89600	-0.41580	33.07960	H	17.10430	-3.69530	30.15420
H	7.16740	0.67140	33.35020	H	17.22830	-3.47140	34.44830
H	5.92020	-2.52990	33.81170	H	15.91530	-3.42380	32.31570
H	7.15000	-3.13260	34.93310	H	21.39510	-2.21020	34.81740
H	9.11010	2.36640	28.81930	H	20.47460	0.54870	35.09710
H	6.49770	2.03930	30.09440	H	20.01930	0.01640	37.52270
H	7.48590	0.05200	31.19890	H	20.34750	-1.67790	37.20050
H	9.91830	0.73490	30.81710	H	18.41270	2.42660	46.19100
H	9.73930	0.21500	29.14770	H	17.02580	4.57760	44.78750
H	7.13570	-1.49560	29.28020	H	14.93600	3.36840	45.44850
H	5.99160	-0.15940	29.18740	H	15.89390	2.08750	46.16750
H	7.41990	-0.17730	28.14080	H	16.91850	1.56520	43.92620
H	10.79200	-1.51180	30.62150	H	15.97570	2.86600	43.19240
H	9.34310	-1.56650	31.61930	H	13.85990	1.65060	43.83760
H	9.26860	-2.12920	29.94430	H	14.84280	0.35450	44.53860
H	8.28760	-6.75020	29.03810	H	15.80560	-0.16910	42.32300
H	6.09320	-6.08350	30.83940	H	14.93060	1.19990	41.58550
H	7.26120	-3.95890	31.25860	H	12.86770	0.22070	42.17910
H	8.47060	-4.33000	30.04800	H	13.68150	-0.89610	41.21040
H	9.31170	-6.22580	31.41200	H	13.64780	-1.16960	42.81590
H	10.38060	-4.86600	33.21890	H	10.71490	-7.49710	44.00570

H	12.09290	-9.80520	45.09880
H	11.38980	-10.12030	42.77170
H	13.13870	-10.00370	42.87190
H	16.50430	-4.47750	43.07950
H	19.04630	-3.05360	43.00660
H	18.20870	-2.87090	40.80380
H	17.79120	-1.36410	41.59970
H	14.84240	-2.38530	39.60460
H	16.53470	-2.09890	39.21940
H	12.90690	-6.32200	38.82920
H	12.45770	-7.58170	39.69860
H	13.95420	-4.40060	41.71610
H	13.70450	-5.81460	42.37840
C	13.99020	1.08550	30.52990
C	13.32520	0.28600	31.63840
O	12.09980	0.31630	31.80100
N	14.15260	-0.44640	32.41970
C	13.68630	-1.07450	33.64570
H	13.04960	-0.37120	34.18460
C	14.88590	-1.39920	34.53940
H	15.48920	-2.18200	34.06580
O	15.63390	-0.21920	34.70370
C	14.34830	-1.94310	35.86070
H	13.77700	-1.14630	36.35220
O	15.39380	-2.40150	36.71390
C	13.40340	-3.13110	35.61330
H	13.98820	-3.97410	35.22970
C	12.67340	-3.54470	36.87970
O	11.87220	-2.47840	37.35640
P	11.73070	-2.53260	39.92380
O	10.48950	-1.71100	39.54810
O	11.64540	-4.03030	39.69790
O	13.09460	-1.84540	39.81520
O	12.37050	-2.80120	34.66260
C	12.82300	-2.31930	33.42290
H	11.91470	-2.05880	32.86960
O	13.58280	-3.28960	32.71750
H	15.03900	0.82190	30.37150
H	13.92650	2.14930	30.78010
H	13.43210	0.92720	29.60360
H	15.14220	-0.23220	32.41310
H	16.58540	-0.45890	34.87440
H	16.18840	-1.79620	36.76300
H	12.06510	-4.43370	36.67330
H	13.39830	-3.80600	37.65310
H	13.04640	-4.09470	32.70060
H	10.17270	-0.30630	45.25570
H	7.35980	-6.66370	39.98520
H	10.84210	1.62910	41.83780
H	13.28280	3.99080	34.04430
H	7.66280	2.23300	28.08680
H	8.47770	3.63940	31.15090
H	7.73090	-7.60330	30.30590
H	5.52350	-5.82930	28.34790
H	17.88230	-6.67480	39.31610
H	17.95610	-8.74010	37.78890
H	20.08880	-6.38260	31.19160
H	21.43150	-5.66920	34.62670
H	19.99150	-1.59250	34.19870
H	22.39360	0.87230	36.60580
H	18.66450	2.96450	44.67660
H	17.80520	5.16840	47.18860
H	10.05020	-8.78890	44.76580
H	13.20240	-7.82370	45.97800
H	17.92390	-5.07430	42.54280
H	17.55540	-2.73720	45.11230
H	10.96480	-2.63400	37.00310

M1-Glc6P-R

N	11.31890	1.98010	44.93400
C	11.21030	1.04430	43.81700
C	9.84280	1.24950	43.14720
O	8.90090	1.74380	43.76260
C	11.32650	-0.45100	44.19890
C	11.54020	-1.35940	42.98610
O	11.00270	-2.50570	43.01900
O	12.26250	-0.90950	42.04650
N	9.73770	0.80130	41.86020
C	8.45110	0.81180	41.17950
C	7.76370	-0.55610	41.07670
O	6.53980	-0.62790	41.15840
C	8.55070	1.52240	39.80030
C	8.82010	3.02170	40.04570
C	7.28890	1.30120	38.95440

C	9.17300	3.81650	38.78510	O	2.66470	-2.38540	32.06670
N	8.53720	-1.64320	40.78570	N	15.94820	-8.37070	39.72010
C	7.89870	-2.90960	40.46030	C	14.68240	-9.05600	40.00110
C	7.90310	-3.80090	41.70180	C	14.49660	-10.17300	38.97070
O	8.93370	-4.24000	42.17450	O	13.42930	-10.66020	38.66910
C	8.59620	-3.64350	39.29410	C	13.43520	-8.14340	40.15290
C	8.55010	-2.85650	38.00940	C	13.15930	-7.28560	38.94820
O	9.05410	-1.63200	37.97940	C	13.79400	-6.10720	38.59210
O	8.02070	-3.33340	37.00140	C	12.17480	-7.52570	37.91990
N	14.03780	2.09320	41.05370	N	13.27320	-5.62190	37.41610
C	13.58820	1.49270	39.78790	C	12.25970	-6.44990	36.98980
C	14.80580	1.35490	38.85990	C	11.21580	-8.53400	37.71710
O	15.66500	2.24110	38.83050	C	11.39440	-6.33830	35.89130
C	12.47100	2.30700	39.07450	C	10.35680	-8.42630	36.63270
O	11.88630	1.53430	38.02910	C	10.44160	-7.33630	35.73880
C	12.93480	3.62870	38.47540	N	17.51000	-8.45650	30.70890
N	14.79440	0.26360	38.05640	C	17.68740	-8.13950	32.11730
C	15.61490	0.13850	36.86570	C	17.42730	-9.38920	32.94620
C	14.87110	0.48970	35.56500	O	16.95440	-10.41460	32.50820
O	15.41190	0.35050	34.47130	C	16.80200	-6.96670	32.67190
N	13.61020	0.99170	35.70160	C	15.36630	-7.38680	32.85520
C	12.96870	1.60490	34.55760	C	14.51720	-7.56200	31.75360
C	13.73050	2.83240	34.07700	C	14.88450	-7.73090	34.12780
O	14.41710	3.53540	34.78070	C	13.23440	-8.09070	31.91700
C	11.55260	2.10240	34.92400	C	13.60380	-8.25470	34.29630
C	10.59580	0.94630	35.18900	C	12.77650	-8.44240	33.18750
C	9.25210	1.37170	35.78430	N	19.65660	-5.60560	34.30250
N	8.34390	0.22190	35.89830	C	19.88880	-4.17250	34.12350
C	7.89980	-0.42570	34.80750	C	21.30860	-3.91870	33.63600
N	7.77620	0.19730	33.63160	O	22.20780	-4.73280	33.67370
N	7.54950	-1.71210	34.88980	C	19.62770	-3.31680	35.39280
N	8.92840	3.91220	29.69640	C	18.14560	-3.33050	35.88130
C	8.19340	2.95740	30.52820	O	17.29710	-3.88060	35.11810
C	8.03690	3.58150	31.90740	O	17.94090	-2.76590	36.98760
O	7.96120	2.97180	32.95800	N	21.49170	1.30030	43.30710
C	8.68950	1.48840	30.55300	C	20.54270	2.41830	43.36190
C	10.10720	1.36040	31.14810	C	21.08790	3.43830	44.37040
C	8.58250	0.86570	29.15440	O	20.40580	4.12730	45.09590
C	10.62540	-0.07820	31.25240	C	19.06470	2.05340	43.60640
N	4.07750	-3.45250	29.05330	C	18.48080	1.16440	42.49690
C	4.04670	-3.64030	30.50690	C	17.01470	0.76570	42.73280
C	2.75720	-2.99280	31.02450	C	16.50130	-0.12310	41.59750

N	15.04720	-0.44140	41.75950	H	11.62990	0.64470	38.38620
N	11.38390	-5.35200	46.16530	H	12.07460	4.19500	38.10970
C	11.48200	-6.78660	45.88800	H	13.45160	4.23390	39.22500
C	12.95450	-7.16100	45.75350	H	13.62390	3.46370	37.64250
O	13.37330	-8.08790	45.09390	H	14.22360	-0.52700	38.36360
C	10.66010	-7.34690	44.71360	H	16.02900	-0.87050	36.77060
C	10.99460	-6.76380	43.33610	H	16.45590	0.83320	36.94050
O	11.44510	-5.56230	43.30040	H	13.20810	1.15440	36.62270
O	10.75590	-7.48690	42.34140	H	12.92440	0.88220	33.73180
N	18.05290	-5.50070	41.89510	H	11.16440	2.72440	34.10590
C	18.68830	-4.20940	41.64030	H	11.63110	2.74370	35.80760
C	18.62920	-3.37860	42.92980	H	11.06390	0.24320	35.88290
O	18.80320	-2.17740	42.98090	H	10.42030	0.41480	34.24760
C	18.22170	-3.43530	40.38660	H	8.77890	2.16690	35.19960
C	16.70370	-3.27760	40.33290	H	9.39940	1.76260	36.79080
O	16.04780	-3.33010	41.40180	H	8.50690	-0.37990	36.71000
N	16.15170	-3.05260	39.13880	H	7.64270	-0.38330	32.81190
Mg	11.37790	-4.15160	41.83690	H	7.91820	1.19400	33.51540
O	10.90770	-5.69670	40.52330	H	7.18600	-2.14320	34.05310
O	13.40340	-3.48640	42.09100	H	7.71630	-2.28730	35.73580
H	10.37770	2.14260	45.28930	H	9.92270	3.87350	29.90670
H	12.00120	1.24370	43.08450	H	7.15710	2.95300	30.14590
H	10.45660	-0.78260	44.77270	H	7.99630	0.94200	31.19980
H	12.20980	-0.57840	44.84080	H	10.11150	1.81020	32.14720
H	10.54810	0.37500	41.41880	H	10.80950	1.94840	30.53940
H	7.77990	1.38930	41.81730	H	8.75060	-0.21260	29.19790
H	9.41240	1.09170	39.27090	H	7.58840	1.02920	28.72120
H	9.63540	3.11740	40.77200	H	9.32470	1.29210	28.46940
H	7.93600	3.46100	40.52790	H	11.59810	-0.10930	31.74930
H	7.33680	1.87050	38.02320	H	9.92810	-0.70830	31.81440
H	7.16140	0.24960	38.69010	H	10.75750	-0.53380	30.26550
H	6.39460	1.61010	39.50460	H	4.38460	-2.50810	28.82890
H	9.45850	4.84330	39.03800	H	3.90420	-4.72040	30.67840
H	10.01270	3.35380	38.25920	H	15.80070	-7.72790	38.94190
H	8.33130	3.87610	38.08730	H	14.81620	-9.59500	40.95320
H	9.54950	-1.61850	40.84030	H	13.58620	-7.51480	41.03860
H	6.86380	-2.67130	40.20330	H	12.56350	-8.76470	40.37760
H	8.10160	-4.59840	39.10830	H	14.56110	-5.54830	39.11170
H	9.63740	-3.84990	39.55460	H	13.52390	-4.75650	36.94900
H	14.74230	2.79730	40.83390	H	11.17020	-9.38120	38.39220
H	13.15740	0.51510	40.00560	H	11.48990	-5.52890	35.17670
H	11.71030	2.50280	39.84940	H	9.60630	-9.19440	36.46560

H	9.75370	-7.28180	34.89840	C	6.58010	-3.84290	30.89100
H	16.69520	-9.05460	30.59740	C	7.76620	-3.24110	31.64370
H	18.72880	-7.83480	32.30380	O	7.76140	-2.03400	31.94230
H	16.89000	-6.13720	31.96010	N	8.78550	-4.05170	31.95630
H	17.23030	-6.59240	33.60460	H	5.38470	-2.07530	31.16430
H	14.86300	-7.28060	30.76320	H	5.07930	-3.30630	32.36530
H	15.52280	-7.58200	34.99510	H	6.52630	-4.92650	31.05280
H	12.58900	-8.21350	31.05150	H	6.76520	-3.69200	29.81870
H	13.24080	-8.50100	35.28730	H	9.54980	-3.71800	32.55890
H	11.77430	-8.83450	33.32780	H	8.74490	-5.03950	31.75820
H	20.47190	-6.01370	34.75550	O	13.61260	-1.65920	39.68800
H	19.20090	-3.79780	33.35190	P	12.21170	-2.07210	39.21810
H	19.88300	-2.26220	35.22000	O	11.50680	-3.16070	40.01830
H	20.26820	-3.65730	36.21560	O	11.18470	-0.89460	38.96520
H	21.31560	0.66500	44.08370	O	12.60800	-2.62790	37.71970
H	20.61910	2.93810	42.39140	C	11.69830	-2.67230	36.63030
H	18.48580	2.97910	43.70620	C	12.51430	-2.62760	35.35490
H	18.98800	1.54040	44.57530	H	13.20170	-1.77550	35.41850
H	19.07170	0.24580	42.40920	O	13.26210	-3.84290	35.29540
H	18.55680	1.69010	41.53410	C	14.15690	-3.93500	34.17680
H	16.38560	1.66190	42.80920	H	14.62950	-4.91720	34.30160
H	16.93190	0.22360	43.68430	O	15.08290	-2.90640	34.16200
H	17.04800	-1.06270	41.57460	C	13.34410	-3.87630	32.86850
H	16.60090	0.38300	40.63390	H	14.06280	-3.83960	32.03650
H	14.85490	-1.00550	42.58750	O	12.44040	-4.96120	32.73570
H	14.52020	0.46580	41.80940	C	12.49830	-2.59070	32.83510
H	14.62490	-0.96810	40.95850	H	11.81990	-2.64780	31.97610
H	11.49520	-4.87040	45.27180	O	13.29860	-1.42870	32.66690
H	11.16410	-7.31770	46.80220	C	11.64470	-2.42730	34.10970
H	9.59660	-7.14140	44.89300	H	11.28460	-1.39560	34.10690
H	10.77750	-8.43130	44.65110	O	10.51870	-3.32150	34.06560
H	17.04570	-5.35980	41.94110	H	11.10110	-3.59130	36.67380
H	19.76740	-4.39850	41.51560	H	11.02520	-1.81060	36.65130
H	18.58200	-3.90520	39.46580	H	15.91540	-3.18840	34.64920
H	18.65800	-2.42830	40.40020	H	12.91390	-5.78830	32.90430
H	15.17270	-2.75790	39.12500	H	14.11690	-1.52320	33.19380
H	16.72510	-2.98440	38.27790	H	9.88480	-3.05910	34.74910
H	11.52940	-5.85830	39.80010	H	9.95340	-1.36180	38.55740
H	10.85420	-6.53190	41.08960	H	11.86390	1.56400	45.68370
H	14.26220	-3.67340	41.66420	H	6.92930	-4.02160	42.17960
H	13.19190	-2.57710	41.79420	H	13.54580	3.08970	33.00650
C	5.27060	-3.15620	31.29700	H	8.82770	3.67490	28.71450

H	13.26560	2.55040	41.53280
H	7.98160	4.68890	31.89140
H	4.73700	-4.08940	28.61740
H	1.89220	-3.12850	30.33870
H	16.19410	-7.79080	40.51970
H	15.45230	-10.53430	38.52840
H	17.36370	-7.60960	30.16830
H	17.68550	-9.28660	34.02350
H	18.85700	-5.70750	34.92230
H	21.48840	-2.88210	33.26810
H	21.34580	0.75610	42.46200
H	22.19940	3.49440	44.37420
H	10.44170	-5.13820	46.48360
H	13.63270	-6.50490	46.34430
H	18.24110	-6.14490	41.13170
H	18.45520	-3.98710	43.84180

M1-Glc6P-TS

N	2.62120	-5.45230	-0.39480
C	3.49170	-4.27630	-0.34320
C	4.34270	-4.28140	-1.61680
O	4.81360	-5.32940	-2.06040
C	4.45880	-4.16560	0.87160
C	4.71150	-2.70000	1.24390
O	5.90790	-2.31770	1.45120
O	3.68220	-1.97780	1.34150
N	4.56050	-3.05570	-2.14160
C	5.45100	-2.82900	-3.26710
C	6.70180	-2.05960	-2.83450
O	7.83160	-2.41280	-3.17530
C	4.68330	-2.12100	-4.41820
C	3.72820	-3.13740	-5.07510
C	5.63410	-1.47890	-5.43640
C	2.61340	-2.50020	-5.90920
N	6.50170	-0.93370	-2.10190
C	7.65370	-0.08470	-1.83880
C	8.51380	-0.82180	-0.77620
O	8.17580	-0.90820	0.39310
C	7.28140	1.33490	-1.36980
C	6.17920	2.12200	-2.06060
O	5.17290	1.47470	-2.61630
O	6.21230	3.36050	-2.04170
N	0.19960	-3.32330	-0.62810
C	0.39580	-2.01430	-1.26480
C	-0.79060	-1.11630	-0.88050
O	-1.95140	-1.53110	-0.94940
C	0.59920	-2.02380	-2.80550
O	0.99150	-0.71420	-3.23540
C	-0.61510	-2.45150	-3.61260
N	-0.43680	0.12600	-0.47720
C	-1.35250	1.23170	-0.29030
C	-1.11910	2.34500	-1.32020
O	-1.48360	3.50050	-1.11990
N	-0.51190	1.95140	-2.47410
C	-0.54000	2.82600	-3.62280
C	-1.96500	3.10540	-4.07290
O	-2.93550	2.45060	-3.77890
C	0.22520	2.18340	-4.80050
C	1.73340	2.27330	-4.57710
C	2.56400	1.33360	-5.45520
N	4.00100	1.60350	-5.27380
C	4.53120	2.78110	-5.63810
N	4.03250	3.48040	-6.66170
N	5.62780	3.26250	-5.02410
N	0.38460	6.47900	-9.81660
C	1.53590	5.78070	-9.24370
C	1.20530	4.29620	-9.19290
O	1.73980	3.48560	-8.45890
C	2.09920	6.29800	-7.89820
C	1.04920	6.21240	-6.77340
C	2.68950	7.70490	-8.05030
C	1.62060	6.44510	-5.37450
N	6.81660	9.09130	-8.92470
C	7.78630	8.46350	-8.02250
C	8.89630	7.85010	-8.88740
O	9.48300	6.82500	-8.62540
N	4.50220	4.18990	7.00200
C	5.86810	3.76880	6.68410
C	6.75900	5.01220	6.60100
O	7.83670	5.04620	6.04850
C	6.02990	2.82450	5.46250
C	5.47970	3.38640	4.18080
C	4.16950	3.35200	3.74590
C	6.22680	4.04610	3.13480

N	4.06400	3.93190	2.49710	N	2.32750	0.30670	6.81760
C	5.30960	4.36370	2.09110	C	0.90660	0.08760	6.54760
C	7.58900	4.36440	2.97220	C	0.63300	-1.42130	6.59050
C	5.71510	4.99100	0.90320	O	-0.32060	-1.96410	6.06920
C	7.99420	4.95670	1.78470	C	0.31840	0.78200	5.30110
C	7.06850	5.26240	0.76330	C	1.17010	0.53560	4.05580
N	2.29860	11.76590	3.92860	O	1.88730	-0.48040	3.98420
C	1.63240	10.61740	4.52200	N	1.05520	1.43870	3.06590
C	2.44420	10.11940	5.70840	Mg	6.59420	-0.47680	1.97090
O	3.55280	10.51620	5.99300	O	7.15700	1.46420	1.75240
C	1.35560	9.40660	3.56210	O	4.74590	0.03500	2.80310
C	2.64040	8.69710	3.21340	H	3.16470	-6.22330	-0.78110
C	3.54350	9.24060	2.28840	H	2.86470	-3.38180	-0.32000
C	3.02850	7.55200	3.92370	H	5.39450	-4.70160	0.69740
C	4.81470	8.68440	2.11730	H	3.97110	-4.61680	1.74600
C	4.29450	6.99460	3.75250	H	4.03950	-2.24760	-1.78770
C	5.20070	7.56850	2.86090	H	5.81290	-3.80270	-3.60330
N	-1.13570	7.29750	5.10470	H	4.08480	-1.32810	-3.95030
C	-2.29560	6.73850	4.41110	H	3.27500	-3.75560	-4.29200
C	-3.58790	7.16910	5.09520	H	4.32140	-3.82290	-5.69590
O	-3.65400	7.63590	6.21350	H	5.07760	-1.04470	-6.27220
C	-2.27760	5.18910	4.28570	H	6.21910	-0.67320	-4.98330
C	-1.04110	4.61550	3.51900	H	6.33860	-2.21690	-5.83400
O	-0.28660	5.45000	2.93460	H	1.98350	-3.26680	-6.37260
O	-0.92290	3.36500	3.56430	H	1.96980	-1.87690	-5.28050
N	-4.51430	-3.65790	5.59540	H	3.01060	-1.87290	-6.71520
C	-4.29800	-4.38600	4.34000	H	5.58330	-0.62250	-1.80420
C	-4.95860	-5.75870	4.46660	H	8.24940	-0.04280	-2.75800
O	-4.57450	-6.76640	3.91500	H	8.17650	1.96140	-1.36850
C	-2.84800	-4.47430	3.83720	H	6.94500	1.29360	-0.33220
C	-2.27390	-3.10660	3.45340	H	-0.76940	-3.60800	-0.75540
C	-0.83140	-3.18120	2.93910	H	1.31770	-1.58670	-0.87210
C	-0.39960	-1.83710	2.36170	H	1.42620	-2.72730	-2.99290
N	0.97070	-1.92580	1.77740	H	1.83940	-0.52790	-2.76050
N	9.80840	-2.52710	4.75240	H	-0.36350	-2.47080	-4.67660
C	9.20350	-1.75870	5.84020	H	-0.93700	-3.45310	-3.31340
C	7.93380	-2.47050	6.30680	H	-1.44950	-1.76600	-3.45300
O	7.07820	-1.95400	6.99050	H	0.56080	0.32040	-0.31860
C	8.97520	-0.25100	5.60800	H	-1.25790	1.67300	0.70740
C	8.38060	0.13270	4.25390	H	-2.37570	0.86480	-0.41340
O	7.74180	-0.76680	3.59950	H	-0.23580	0.98200	-2.60330
O	8.54920	1.32090	3.88020	H	-0.09610	3.79910	-3.36710

H	-0.04460	2.68110	-5.74140	H	6.18410	7.13020	2.73010
H	-0.09260	1.13770	-4.88700	H	-1.24560	7.14460	6.10500
H	1.96010	2.05040	-3.53240	H	-2.31150	7.13610	3.38630
H	2.05060	3.30710	-4.75920	H	-3.16980	4.82460	3.75880
H	2.30370	1.41930	-6.51640	H	-2.30020	4.73070	5.28210
H	2.38840	0.29650	-5.16850	H	-3.82290	-3.95440	6.28270
H	4.43810	1.18660	-4.45410	H	-4.90370	-3.87250	3.57310
H	4.32120	4.45080	-6.71910	H	-2.81780	-5.15630	2.97950
H	3.20690	3.20770	-7.17970	H	-2.23070	-4.93680	4.62020
H	5.85950	4.21950	-5.27270	H	-2.29490	-2.43160	4.31740
H	5.75020	3.04340	-4.04290	H	-2.91100	-2.65960	2.67620
H	-0.35030	6.57890	-9.12080	H	-0.74950	-3.94750	2.15670
H	2.34110	5.83700	-9.99650	H	-0.16220	-3.46270	3.76190
H	2.92220	5.63000	-7.63350	H	-0.39760	-1.06440	3.12540
H	0.58340	5.21950	-6.79510	H	-1.07060	-1.53580	1.55640
H	0.24490	6.93730	-6.96340	H	1.69180	-2.12890	2.47500
H	3.16250	8.02660	-7.12080	H	0.93810	-2.65540	1.03070
H	3.45260	7.73170	-8.83670	H	1.31280	-1.04820	1.33820
H	1.91690	8.44010	-8.30250	H	9.24480	-2.37930	3.91580
H	0.86200	6.27560	-4.60810	H	9.87320	-1.85020	6.71160
H	2.46340	5.77270	-5.18130	H	9.90790	0.30980	5.72160
H	1.99090	7.46760	-5.25020	H	8.28640	0.12130	6.37750
H	6.18860	8.38670	-9.30770	H	2.86170	0.01230	6.00110
H	8.28480	9.28430	-7.47980	H	0.34950	0.46600	7.42120
H	4.03450	4.47210	6.14110	H	0.18240	1.85650	5.46120
H	6.25160	3.23350	7.56760	H	-0.68400	0.37970	5.10450
H	5.52260	1.88120	5.69940	H	1.53720	1.23920	2.19050
H	7.08960	2.58820	5.33160	H	0.40020	2.24020	3.13900
H	3.30020	2.89610	4.19940	H	6.52950	2.19990	1.76240
H	3.21000	4.15570	1.99460	H	7.76250	1.57930	2.56760
H	8.29500	4.12500	3.75800	H	4.45660	0.85980	2.38420
H	4.99970	5.28240	0.14390	H	4.14540	-0.64180	2.38620
H	9.04520	5.18780	1.63650	C	7.22670	7.44770	-7.01500
H	7.41660	5.72150	-0.15710	C	6.22070	8.05180	-6.02430
H	3.30420	11.61200	3.93960	C	5.47090	6.95720	-5.26960
H	0.65300	10.92510	4.92190	O	5.08000	5.95220	-5.89510
H	0.86180	9.80710	2.66830	N	5.24640	7.12280	-3.96320
H	0.63710	8.72800	4.03430	H	6.72870	6.64130	-7.56410
H	3.25430	10.11150	1.70690	H	8.05870	6.98150	-6.47670
H	2.32870	7.09900	4.62140	H	6.71080	8.75040	-5.33610
H	5.50470	9.12440	1.40200	H	5.45940	8.62180	-6.57350
H	4.57770	6.10270	4.30230	H	4.74640	6.41320	-3.40070

H	5.58290	7.93970	-3.47720
O	2.11010	0.36650	0.39360
P	3.31670	0.34860	-0.55430
O	4.67200	0.61250	0.05790
O	3.24600	-0.61770	-1.73940
O	3.02920	1.94530	-1.57040
C	3.17700	2.98500	-0.56340
C	2.31710	4.20010	-0.81740
H	1.26520	3.90050	-0.89520
O	2.51510	4.97320	0.38010
C	1.71650	6.16470	0.46370
H	2.02150	6.60960	1.42000
O	0.36110	5.89140	0.42290
C	2.05600	7.09250	-0.71610
H	1.32160	7.91180	-0.70610
O	3.38540	7.58500	-0.65990
C	1.89980	6.33520	-2.04820
H	2.29400	6.97560	-2.84280
O	0.53160	6.07980	-2.34430
C	2.67600	4.99930	-2.07090
H	2.31270	4.43530	-2.93740
O	4.07400	5.26540	-2.21580
H	2.86530	2.57760	0.39630
H	4.22640	3.26290	-0.47120
H	0.06790	5.57020	1.32410
H	3.60920	7.76710	0.26520
H	0.08290	5.78910	-1.52590
H	4.62450	4.45840	-2.16410
H	4.13200	1.81070	-2.20010
H	2.32860	-5.71610	0.54230
H	9.44210	-1.30500	-1.12380
H	-2.04270	3.97550	-4.76990
H	0.64580	7.41770	-10.10100
H	0.81600	-4.04990	-0.99880
H	0.42390	3.98120	-9.91490
H	6.23770	9.76300	-8.43010
H	9.12180	8.44740	-9.79840
H	3.97610	3.40050	7.36920
H	6.34140	5.89720	7.13130
H	2.00690	11.88670	2.96360
H	1.94050	9.32680	6.30380
H	-0.32540	6.77650	4.77780
H	-4.50990	6.97650	4.49860
H	-4.35640	-2.66440	5.45350
H	-5.85890	-5.75230	5.12160
H	10.73330	-2.15960	4.54770
H	7.87810	-3.53610	5.99460
H	2.50580	1.29890	6.94880
H	1.38000	-1.98820	7.18500

M1-Glc6P-P

N	13.37710	1.57540	44.68550
C	12.98040	0.59480	43.68710
C	11.43530	0.53510	43.68510
O	10.80960	0.71050	44.72910
C	13.54150	-0.83650	43.94690
C	13.23680	-1.78550	42.81310
O	12.68520	-2.87120	42.99020
O	13.62720	-1.31060	41.63940
N	10.87570	0.25260	42.48780
C	9.43280	0.16110	42.28690
C	8.99610	-1.30240	42.11540
O	8.26030	-1.87480	42.92670
C	9.01170	1.03430	41.07240
C	9.34620	2.51420	41.33510
C	7.53060	0.82350	40.72920
C	9.14490	3.41830	40.11280
N	9.42400	-1.92930	41.00220
C	8.78300	-3.19270	40.67760
C	9.03010	-4.13700	41.84880
O	10.15740	-4.43000	42.22390
C	9.31560	-3.81870	39.37600
C	9.22230	-2.92890	38.14280
O	9.31970	-1.66910	38.31400
O	9.11270	-3.48680	37.01330
N	14.48450	2.51120	40.34290
C	13.79140	1.85590	39.22280
C	14.74860	1.77600	38.03500
O	15.54430	2.69200	37.80030
C	12.44210	2.50910	38.81350
O	11.70250	1.60710	37.99490
C	12.56590	3.82830	38.06460
N	14.61910	0.65750	37.28080
C	15.21400	0.52950	35.96380

C	14.26560	0.90100	34.81360
O	14.64180	0.82450	33.64520
N	13.02920	1.33800	35.16150
C	12.14040	1.83620	34.13160
C	12.71760	3.08030	33.45780
O	13.41800	3.89600	34.01040
C	10.78140	2.22980	34.75090
C	9.99520	1.00570	35.22300
C	8.86130	1.33510	36.19990
N	8.14130	0.11390	36.57800
C	7.38850	-0.56430	35.70780
N	6.78820	0.03860	34.66490
N	7.14550	-1.87180	35.90860
N	6.93770	2.83810	29.73780
C	6.60450	2.06030	30.93350
C	6.72380	2.98810	32.13480
O	6.94980	2.63650	33.27710
C	7.31530	0.69780	31.13150
C	8.84760	0.84840	31.23410
C	6.88930	-0.29340	30.04120
C	9.58470	-0.45650	31.55270
N	3.61460	-3.51440	30.10250
C	3.71480	-4.29880	31.33690
C	2.34580	-4.27120	32.02840
O	2.18060	-4.37070	33.22300
N	17.68480	-7.86340	38.46770
C	16.58340	-8.54850	39.14840
C	15.99140	-9.58920	38.19500
O	14.93630	-10.15480	38.37850
C	15.49050	-7.63560	39.76450
C	14.77200	-6.81000	38.73370
C	15.20380	-5.63420	38.14110
C	13.50480	-7.11990	38.11470
N	14.27920	-5.19770	37.22040
C	13.22120	-6.07920	37.18490
C	12.57620	-8.16460	38.28310
C	12.03820	-6.04890	36.42770
C	11.40250	-8.13120	37.54430
C	11.13770	-7.08460	36.63260
N	16.38590	-8.18200	29.93590
C	17.28700	-7.51040	30.85880
C	17.88980	-8.53190	31.80940
O	17.52750	-9.68420	31.89560
C	16.66990	-6.34040	31.70180
C	15.72860	-6.85190	32.76460
C	14.45710	-7.34490	32.43490
C	16.14600	-6.93690	34.10050
C	13.64670	-7.94150	33.40390
C	15.33450	-7.52650	35.06850
C	14.08730	-8.04550	34.72420
N	19.73240	-4.50550	32.68730
C	19.75710	-3.04580	32.62540
C	21.02480	-2.56320	31.92770
O	22.00360	-3.25040	31.72220
C	19.62550	-2.34230	34.00570
C	18.28430	-2.63180	34.75310
O	17.41360	-3.31510	34.13570
O	18.19670	-2.13970	35.90860
N	22.06950	3.18390	41.05940
C	20.88480	3.93240	41.48820
C	21.31550	4.91310	42.57760
O	20.62310	5.25980	43.50760
C	19.66220	3.10750	41.91030
C	19.09500	2.23990	40.78280
C	17.83650	1.49130	41.22510
C	17.23330	0.65970	40.09790
N	15.94910	0.05650	40.56570
N	12.67620	-6.60760	45.73530
C	13.71460	-7.45300	45.14500
C	15.03020	-6.68000	45.12830
O	15.96560	-6.93050	44.39900
C	13.42010	-8.10370	43.78310
C	12.94470	-7.17450	42.66050
O	12.78880	-5.93050	42.91500
O	12.72550	-7.73020	41.55320
N	19.10030	-4.52120	41.01680
C	19.57910	-3.27750	40.41860
C	19.65540	-2.20710	41.51120
O	19.69940	-1.01120	41.29920
C	18.86230	-2.78550	39.14640
C	17.34900	-2.66060	39.32770
O	16.83740	-2.69890	40.47440
N	16.63680	-2.49320	38.20940
Mg	12.38010	-4.40460	41.61140

O	11.77380	-5.77750	40.22640
O	14.53120	-4.47970	41.07970
H	12.72580	1.49750	45.46550
H	13.33470	0.91260	42.69950
H	13.13840	-1.24670	44.87500
H	14.63350	-0.76700	44.03900
H	11.44490	0.10870	41.64630
H	8.95120	0.50980	43.20280
H	9.61210	0.70780	40.21270
H	10.38960	2.58150	41.66270
H	8.74160	2.87920	42.17640
H	7.24420	1.41240	39.85300
H	7.32120	-0.22290	40.49220
H	6.88770	1.12040	41.56690
H	9.54830	4.41990	40.29400
H	9.65570	3.00550	39.23670
H	8.08780	3.53450	39.85780
H	9.95890	-1.45930	40.28010
H	7.69730	-3.03560	40.60280
H	8.80310	-4.76180	39.17250
H	10.37320	-4.04990	39.50710
H	15.04520	3.27690	39.96840
H	13.51740	0.85440	39.53880
H	11.89870	2.67590	39.75870
H	11.67270	0.74250	38.45970
H	11.57100	4.25520	37.90630
H	13.16280	4.54370	38.63800
H	13.04680	3.69010	37.09150
H	14.03250	-0.09390	37.64010
H	15.58760	-0.48570	35.79320
H	16.06640	1.21090	35.89910
H	12.72900	1.35610	36.13280
H	12.00630	1.06230	33.36360
H	10.19250	2.79560	34.01630
H	10.97960	2.90920	35.58850
H	10.66300	0.30620	35.73140
H	9.58350	0.48420	34.35060
H	8.16490	2.07720	35.79170
H	9.28310	1.75500	37.11580
H	8.58940	-0.48340	37.29520
H	6.57680	-0.57980	33.89000
H	6.98930	0.99980	34.42130
H	6.66830	-2.32870	35.14330
H	7.88430	-2.44500	36.34960
H	7.94600	2.94710	29.66120
H	5.51840	1.86720	30.88040
H	6.96230	0.29660	32.08380
H	9.07460	1.58190	32.01780
H	9.24130	1.26330	30.29510
H	7.27210	-1.29130	30.25970
H	5.79670	-0.36560	29.97780
H	7.26640	0.00470	29.05570
H	10.65010	-0.28280	31.72220
H	9.16340	-0.93220	32.44350
H	9.50320	-1.17800	30.73320
H	3.69950	-2.52150	30.31350
H	3.84530	-5.35120	31.03230
H	17.29910	-7.17620	37.82110
H	17.02720	-9.13920	39.96450
H	15.97170	-6.99050	40.51040
H	14.76410	-8.24300	40.30890
H	16.09670	-5.05260	38.32040
H	14.35750	-4.41250	36.57800
H	12.77960	-8.96000	38.98920
H	11.84730	-5.26830	35.70040
H	10.66820	-8.92120	37.67390
H	10.20540	-7.08450	36.07500
H	15.86580	-8.89810	30.43660
H	18.12850	-7.07170	30.29930
H	16.15320	-5.67390	31.00020
H	17.47710	-5.74890	32.14400
H	14.09850	-7.26000	31.41320
H	17.10770	-6.51590	34.38230
H	12.66470	-8.31760	33.12950
H	15.65940	-7.56290	36.10310
H	13.45700	-8.49400	35.48450
H	20.65180	-4.83930	32.96890
H	18.90230	-2.70690	32.02190
H	19.69410	-1.25090	33.90430
H	20.45380	-2.64240	34.65960
H	22.25730	2.43460	41.72200
H	20.60030	4.57710	40.64010
H	18.89600	3.79400	42.28740
H	19.94440	2.47230	42.76100

H	19.84210	1.50760	40.45620
H	18.85700	2.87100	39.91470
H	17.08470	2.21060	41.57560
H	18.08730	0.83170	42.06460
H	17.90540	-0.14180	39.80020
H	16.99870	1.27930	39.23060
H	16.10000	-0.61490	41.31980
H	15.34020	0.85060	40.86380
H	15.40280	-0.49370	39.85460
H	12.39180	-5.93800	45.02060
H	13.91410	-8.27000	45.86030
H	12.65540	-8.88150	43.89170
H	14.32240	-8.60510	43.41720
H	18.10020	-4.44100	41.18910
H	20.63710	-3.43380	40.14960
H	19.06770	-3.44720	38.29830
H	19.25600	-1.80690	38.84690
H	15.64020	-2.31110	38.33580
H	17.09630	-2.41380	37.27910
H	12.02030	-5.73040	39.29420
H	12.10030	-6.66510	40.59500
H	14.55370	-5.03050	40.27660
H	15.09620	-3.71310	40.86470
C	4.83880	-3.90310	32.30730
C	6.25120	-4.08270	31.73160
C	7.27910	-3.35030	32.59240
O	7.02470	-2.19340	32.97420
N	8.41960	-3.98150	32.89190
H	4.71270	-2.84870	32.57790
H	4.72750	-4.47650	33.23360
H	6.49930	-5.14380	31.61360
H	6.30560	-3.62350	30.73580
H	9.15280	-3.56100	33.48710
H	8.59040	-4.92120	32.56910
O	14.13360	-1.45420	39.18380
P	12.88230	-1.61810	40.05340
O	12.27130	-3.00480	40.13430
O	11.88290	-0.46340	39.98900
O	11.70540	-1.19130	37.10190
C	12.32490	-2.43510	36.79910
C	12.73780	-2.40380	35.33930
H	13.34550	-1.50860	35.17040
O	13.55080	-3.56650	35.12940
C	14.14120	-3.63550	33.82150
H	14.70770	-4.57440	33.84140
O	14.95740	-2.54610	33.56140
C	13.02820	-3.69090	32.76000
H	13.51440	-3.61510	31.77590
O	12.24760	-4.87360	32.86290
C	12.06830	-2.49530	32.91270
H	11.20120	-2.67880	32.26920
O	12.66020	-1.28180	32.46650
C	11.55890	-2.32850	34.36140
H	11.14620	-1.31870	34.43190
O	10.53530	-3.29290	34.62160
H	13.21340	-2.58010	37.41680
H	11.65680	-3.27930	36.99000
H	15.88420	-2.75630	33.87130
H	12.82290	-5.60140	33.13900
H	13.56610	-1.20410	32.81810
H	10.13960	-3.16650	35.51170
H	10.90200	-1.34680	37.63560
H	14.31370	1.38510	45.03040
H	8.15210	-4.60230	42.32610
H	12.37570	3.22690	32.40620
H	6.63620	2.34440	28.90350
H	13.81570	2.89680	41.00590
H	6.56620	4.05920	31.88910
H	4.37220	-3.74220	29.46630
H	1.49630	-4.15970	31.31750
H	18.22710	-7.33740	39.14900
H	16.63210	-9.81340	37.31290
H	15.72190	-7.52240	29.54330
H	18.69850	-8.12800	32.45750
H	19.05440	-4.76040	33.40010
H	21.01070	-1.48500	31.64440
H	21.89250	2.73300	40.16710
H	22.34990	5.29900	42.43870
H	11.85670	-7.17290	45.94300
H	15.07340	-5.86350	45.88320
H	19.24660	-5.30110	40.38210
H	19.70940	-2.62490	42.53900

M1-GlcNAc6P-R

N	9.47340	0.13170	43.68810	N	8.29640	-0.94380	35.26250
C	9.56850	-0.98970	42.74900	C	7.69120	-1.36790	34.15830
C	8.19390	-1.14190	42.08980	N	7.31440	-0.52620	33.16490
O	7.15520	-1.03140	42.74220	N	7.36180	-2.66130	34.04700
C	9.99620	-2.36630	43.33330	N	7.52070	2.70080	28.89380
C	10.86950	-3.14320	42.33590	C	7.65430	1.84640	30.07600
O	10.58000	-4.36400	42.11150	C	7.80050	2.73090	31.30400
O	11.82640	-2.49810	41.82940	O	7.95300	2.29720	32.43330
N	8.24550	-1.43410	40.76960	C	8.74550	0.73540	30.04250
C	7.05890	-1.73820	39.98740	C	10.15950	1.32060	29.85530
C	7.01670	-3.20590	39.55160	C	8.39780	-0.33000	28.99670
O	5.96650	-3.84250	39.50650	C	11.27210	0.26660	29.89490
C	6.95830	-0.76860	38.77430	N	8.21100	-6.19830	29.20810
C	6.56210	0.62700	39.29680	C	7.28630	-5.33300	29.94490
C	5.99300	-1.27800	37.69520	C	6.11480	-5.01480	29.00300
C	6.77280	1.76250	38.29210	O	5.54860	-3.94550	28.95140
N	8.20940	-3.73630	39.14840	C	7.88730	-4.06750	30.58840
C	8.27760	-5.11300	38.66760	C	8.95470	-4.38080	31.64120
C	8.31700	-6.00310	39.91910	C	9.66510	-3.13620	32.19320
O	9.34550	-6.32950	40.48570	H	9.72740	-5.03080	31.20850
C	9.48220	-5.25220	37.71160	N	16.78070	-8.01980	40.75700
C	9.19370	-4.37000	36.50780	C	16.28180	-8.85110	39.65850
O	9.80300	-3.20000	36.34950	C	17.41730	-9.20110	38.70550
O	8.33600	-4.69790	35.68650	O	17.34200	-10.10230	37.89590
N	11.63160	1.49140	41.76210	C	15.10370	-8.23340	38.84010
C	11.70490	1.10270	40.34590	C	15.45820	-6.90050	38.25820
C	12.98720	1.70940	39.76770	C	15.25130	-5.66870	38.85030
O	13.32640	2.85820	40.06980	C	16.25890	-6.65550	37.07900
C	10.48120	1.50540	39.46630	N	15.88390	-4.68610	38.11800
O	10.52010	0.78000	38.23470	C	16.49990	-5.25560	37.02470
C	10.38930	2.98980	39.14080	C	16.80230	-7.47680	36.07240
N	13.66610	0.92220	38.89830	C	17.26100	-4.66280	36.00820
C	14.68400	1.46740	38.02290	C	17.55470	-6.89430	35.06310
C	14.13790	1.90880	36.66060	C	17.77860	-5.50340	35.03090
O	14.84550	2.51560	35.85630	N	22.58780	-5.68240	31.89620
N	12.84950	1.58520	36.36950	C	22.36240	-5.59950	33.33530
C	12.30240	2.09450	35.11650	C	21.10250	-6.32660	33.79690
C	12.69690	3.55930	34.95370	O	20.37410	-6.93880	33.04450
O	12.39500	4.41220	35.76120	C	22.35950	-4.12750	33.83030
C	10.77770	1.95910	35.09540	C	21.19970	-3.31240	33.29680
C	10.30990	0.50400	34.94730	C	21.21610	-2.78330	31.99860
C	8.87260	0.38680	35.45490	C	20.07480	-3.07730	34.09720

C	20.12840	-2.05970	31.50790	H	10.63310	-2.18540	44.21010
C	18.99650	-2.33210	33.61780	H	9.16030	-1.47580	40.30320
C	19.01490	-1.83020	32.31720	H	6.19260	-1.60430	40.63870
N	21.62550	-2.21920	36.91850	H	7.96810	-0.70690	38.34350
C	21.13080	-0.93870	36.41630	H	7.14310	0.84090	40.20160
C	22.30140	-0.04920	36.00770	H	5.50930	0.59430	39.61280
O	23.46710	-0.29320	36.24320	H	5.89640	-0.54780	36.88720
C	20.17190	-0.19940	37.38820	H	6.33910	-2.21520	37.24790
C	18.86750	-0.99090	37.69960	H	4.99990	-1.46640	38.11750
O	18.53690	-1.14020	38.89060	H	6.49670	2.72750	38.73060
O	18.20430	-1.41580	36.68240	H	7.82370	1.82230	37.99420
N	18.36410	3.28020	45.55370	H	6.17190	1.63200	37.38510
C	16.99810	3.81600	45.59190	H	9.08800	-3.23490	39.29670
C	16.86980	4.65120	46.87840	H	7.34350	-5.31870	38.13530
O	15.86370	4.74920	47.54430	H	9.55880	-6.28540	37.36580
C	15.85660	2.79500	45.39120	H	10.40950	-4.97230	38.21700
C	15.91450	2.09520	44.01930	H	11.91700	2.46610	41.84180
C	14.78790	1.07100	43.78820	H	11.74690	0.01470	40.29120
C	14.85890	0.46990	42.37860	H	9.59000	1.19880	40.03820
N	13.69450	-0.43870	42.11870	H	10.50790	-0.18590	38.47430
N	10.79500	-8.38100	44.62400	H	9.43460	3.21000	38.65560
C	12.08710	-9.02480	44.38040	H	10.46090	3.58880	40.05300
C	13.18210	-8.12920	44.95310	H	11.19750	3.29770	38.47150
O	14.29140	-8.00030	44.47870	H	13.42660	-0.07900	38.85760
C	12.39390	-9.49100	42.94850	H	15.47320	0.73190	37.84310
C	12.20690	-8.46500	41.82600	H	15.13390	2.34770	38.48660
O	11.87400	-7.26860	42.13890	H	12.21390	1.23470	37.08520
O	12.39220	-8.89080	40.65870	H	12.71840	1.55050	34.26310
N	17.51710	-4.46420	43.38840	H	10.36850	2.55210	34.26840
C	18.03140	-3.13370	43.06790	H	10.38970	2.39930	36.02060
C	17.58420	-2.17230	44.16470	H	10.94780	-0.17760	35.52100
O	17.43910	-0.97390	44.02160	H	10.36990	0.18770	33.89770
C	17.74600	-2.59390	41.65940	H	8.22310	1.13440	34.98880
C	16.25110	-2.47390	41.35830	H	8.86590	0.58520	36.52710
O	15.39050	-2.77190	42.21310	H	8.62750	-1.67200	35.89280
N	15.96400	-2.01180	40.13260	H	7.38240	-0.95100	32.24790
Mg	11.55540	-5.69800	40.89300	H	7.66150	0.43040	33.15700
O	12.02170	-6.79090	39.21380	H	6.81900	-2.95180	33.25050
O	13.38900	-4.74530	41.38500	H	7.75410	-3.38830	34.66540
H	8.56770	0.07180	44.15330	H	8.43080	3.06320	28.62020
H	10.31400	-0.74120	41.99150	H	6.68210	1.34850	30.21920
H	9.13940	-2.95910	43.66350	H	8.73960	0.25060	31.02240

H	10.34500	2.05400	30.65100	H	14.89810	3.31130	45.52090
H	10.21490	1.87140	28.90430	H	15.91140	2.04700	46.19440
H	9.08370	-1.17500	29.07610	H	16.87170	1.57240	43.91150
H	7.38240	-0.71840	29.14070	H	15.87150	2.85710	43.22750
H	8.46400	0.07110	27.97750	H	13.80990	1.55110	43.92610
H	12.24930	0.74410	29.98670	H	14.87170	0.26560	44.52910
H	11.14330	-0.39570	30.75650	H	15.77690	-0.09980	42.24550
H	11.27000	-0.35480	28.99230	H	14.81110	1.25780	41.62340
H	8.80240	-5.64210	28.59510	H	12.81390	0.12230	42.24700
H	6.83820	-5.95980	30.73510	H	13.65060	-0.83360	41.15900
H	8.31990	-3.43370	29.80800	H	13.69840	-1.27610	42.70570
H	7.07400	-3.47330	31.01880	H	10.69090	-7.63150	43.94060
H	8.52610	-4.93990	32.48540	H	12.12820	-9.91600	45.03130
O	9.44710	-2.00500	31.74480	H	11.76670	-10.35210	42.69120
N	10.53950	-3.38490	33.18860	H	13.43150	-9.83730	42.88860
H	10.64690	-4.31260	33.56660	H	16.52680	-4.47990	43.15660
H	11.07090	-2.65230	33.64570	H	19.12690	-3.17130	43.19120
H	16.80090	-7.04690	40.44680	H	18.19730	-3.24940	40.90720
H	15.92820	-9.80390	40.07320	H	18.21240	-1.61550	41.50970
H	14.26120	-8.15310	39.52610	H	14.99800	-1.99220	39.79410
H	14.82240	-8.94650	38.05840	H	16.72910	-1.73160	39.51330
H	14.70030	-5.40830	39.74560	H	12.80690	-6.47140	38.74390
H	15.74120	-3.68770	38.21240	H	12.21570	-7.70080	39.61200
H	16.64800	-8.54890	36.09400	H	13.08660	-3.80270	41.36400
H	17.46460	-3.59670	36.01650	H	13.80910	-4.85330	42.24750
H	18.00270	-7.50670	34.29050	C	15.78370	1.75100	31.14640
H	18.37060	-5.08420	34.22710	C	14.79820	0.97370	31.99920
H	22.54240	-6.66290	31.62430	O	13.58790	1.17770	31.95870
H	23.20130	-6.09910	33.84720	N	15.36070	0.02860	32.82410
H	23.30970	-3.68700	33.51410	C	14.58420	-0.46950	33.95970
H	22.33600	-4.10140	34.92530	H	14.07140	0.36200	34.43600
H	22.09280	-2.93540	31.37490	C	15.55160	-1.05640	34.98130
H	20.05800	-3.44550	35.11730	H	16.05110	-1.94080	34.54940
H	20.15740	-1.66390	30.49760	O	16.49090	-0.04520	35.27000
H	18.17120	-2.11600	34.28380	C	14.73320	-1.53510	36.18740
H	18.17030	-1.25410	31.94250	H	14.12670	-0.71090	36.57250
H	22.55790	-2.08010	37.29760	O	15.56770	-2.04080	37.21570
H	20.55530	-1.10390	35.49390	C	13.75750	-2.61180	35.67120
H	19.86030	0.75370	36.93910	H	14.34350	-3.41960	35.21010
H	20.67750	0.01900	38.33150	C	12.89600	-3.27130	36.72260
H	18.41720	2.44290	46.13120	O	11.93840	-2.36610	37.35170
H	16.93780	4.56690	44.78540	P	11.86350	-2.46980	38.99670

O	13.13120	-1.72580	39.46240
O	10.51020	-1.73220	39.18030
O	11.79030	-3.97000	39.30970
O	12.87780	-2.02620	34.68100
C	13.53030	-1.50510	33.53870
H	12.73820	-1.01690	32.95900
O	14.10360	-2.56780	32.80720
H	16.13610	2.61590	31.71940
H	15.27550	2.11250	30.25190
H	16.65320	1.15060	30.86450
H	16.32760	0.20010	33.09090
H	17.25970	-0.45240	35.74130
H	16.51770	-1.79640	37.06600
H	12.32310	-4.07930	36.25330
H	13.53930	-3.71810	37.48040
H	14.87750	-2.20450	32.35030
H	10.19500	0.05320	44.39980
H	7.33240	-6.26970	40.34080
H	10.70040	1.37880	42.16830
H	13.19530	3.81240	33.99380
H	7.18460	2.15290	28.10750
H	7.73690	3.82320	31.11940
H	8.82630	-6.70000	29.83970
H	5.82820	-5.87130	28.35510
H	18.33290	-8.57570	38.79970
H	21.78270	-5.26150	31.43350
H	20.87620	-6.26420	34.88400
H	21.02770	-2.55360	37.66680
H	22.00520	0.87620	35.46430
H	18.59870	2.98450	44.61060
H	17.81290	5.17720	47.14740
H	10.04420	-9.04450	44.44940
H	12.87280	-7.59560	45.87880
H	17.97430	-5.16930	42.81650
H	17.42400	-2.66840	45.14620
H	16.12460	-8.05270	41.53400
H	10.62840	-2.90380	36.91110

M1-GlcNAc6P-TS

N	9.61420	0.06720	43.92970
C	9.68470	-1.03280	42.96540
C	8.29800	-1.16100	42.32410
O	7.26930	-1.00510	42.98160
C	10.13130	-2.42740	43.49480
C	10.94970	-3.17680	42.42420
O	10.69270	-4.40890	42.22920
O	11.82640	-2.49810	41.82940
N	8.32880	-1.48080	41.00880
C	7.12690	-1.75510	40.23500
C	7.07410	-3.21580	39.77490
O	6.04900	-3.88810	39.85360
C	7.01220	-0.75860	39.04610
C	6.63100	0.62740	39.60340
C	6.02900	-1.24650	37.97270
C	6.77740	1.77540	38.60190
N	8.21860	-3.70040	39.21520
C	8.25220	-5.06770	38.70320
C	8.31700	-6.00310	39.91910
O	9.35550	-6.24900	40.51100
C	9.43310	-5.21690	37.72190
C	9.17170	-4.26460	36.55740
O	9.75950	-3.10430	36.51400
O	8.33280	-4.60030	35.69910
N	11.68500	1.46810	41.82800
C	11.74290	1.07630	40.41270
C	13.02190	1.67900	39.81830
O	13.37970	2.81820	40.13230
C	10.51010	1.47340	39.54830
O	10.53240	0.73160	38.32610
C	10.42370	2.95400	39.20750
N	13.67230	0.89860	38.91990
C	14.66800	1.44050	38.01460
C	14.08550	1.89840	36.67260
O	14.78500	2.48330	35.84470
N	12.77820	1.62370	36.43160
C	12.19760	2.13720	35.19350
C	12.69690	3.55930	34.95370
O	12.47530	4.47090	35.72140
C	10.66770	2.05830	35.24190
C	10.18700	0.60800	35.06650
C	8.77720	0.42540	35.62930
N	8.24590	-0.91820	35.40030
C	7.59320	-1.29540	34.30490
N	7.20490	-0.40820	33.35870

N	7.21270	-2.57570	34.18600	N	21.24000	-2.05080	35.10080
N	7.56680	2.60280	28.89220	C	20.99420	-0.77370	35.75550
C	7.65320	1.79680	30.11220	C	22.30140	-0.04920	36.00770
C	7.80050	2.73090	31.30400	O	23.40050	-0.55480	35.93530
O	7.93320	2.34240	32.45240	C	20.14160	-0.86180	37.06000
C	8.70220	0.64210	30.13850	C	18.69160	-1.33080	36.77060
C	10.14500	1.16220	29.96570	O	17.88470	-1.45810	37.73670
C	8.33010	-0.43300	29.11020	O	18.40120	-1.51930	35.55440
C	11.20780	0.05710	29.92960	N	18.35780	3.23100	45.60480
N	8.14140	-6.26740	29.39470	C	17.00430	3.79630	45.60770
C	7.19030	-5.35760	30.03990	C	16.86980	4.65120	46.87840
C	6.11480	-5.01480	29.00300	O	15.85340	4.77610	47.52320
O	5.58640	-3.93030	28.89570	C	15.84430	2.79800	45.40620
C	7.78010	-4.09840	30.70170	C	15.90620	2.07560	44.04780
C	8.76140	-4.42150	31.82880	C	14.76830	1.06320	43.82560
C	9.50760	-3.19160	32.36580	C	14.87260	0.40250	42.44570
H	9.52390	-5.12620	31.47250	N	13.69640	-0.48290	42.17370
N	17.60410	-7.43400	40.38150	N	10.78780	-8.31490	44.68850
C	16.74560	-8.52300	39.90360	C	12.05190	-9.00860	44.43060
C	17.41730	-9.20110	38.70550	C	13.18210	-8.12920	44.95310
O	16.88740	-10.06520	38.04370	O	14.26630	-7.99510	44.42340
C	15.27450	-8.12590	39.58400	C	12.30820	-9.51430	43.00460
C	15.19360	-7.12350	38.47040	C	12.16770	-8.49470	41.86790
C	15.43450	-5.76510	38.55860	O	11.85550	-7.28620	42.16370
C	14.98900	-7.40090	37.06810	O	12.36480	-8.93980	40.71230
N	15.41250	-5.19370	37.30470	N	17.69700	-4.36070	43.10890
C	15.12040	-6.16650	36.37270	C	18.08150	-2.96060	42.94910
C	14.69320	-8.56840	36.34460	C	17.58420	-2.17230	44.16470
C	14.93940	-6.07870	34.98680	O	17.38080	-0.97450	44.17720
C	14.51110	-8.47890	34.97240	C	17.73300	-2.28470	41.60800
C	14.63090	-7.24480	34.30070	C	16.26610	-2.46780	41.20780
N	20.94780	-6.52030	31.31100	O	15.40640	-2.77120	42.06240
C	21.44440	-5.73280	32.43210	N	15.98500	-2.25230	39.91670
C	21.10250	-6.32660	33.79690	Mg	11.55540	-5.69800	40.89300
O	20.58840	-7.41210	33.95030	O	12.04570	-6.84520	39.25250
C	20.99040	-4.25590	32.33410	O	13.54660	-5.09860	41.46620
C	19.48610	-4.10880	32.41680	H	8.71290	0.01220	44.40060
C	18.70550	-4.00770	31.25840	H	10.41480	-0.77020	42.19720
C	18.83420	-4.11280	33.65670	H	9.28910	-3.02900	43.84990
C	17.31530	-3.91650	31.34050	H	10.80960	-2.27390	44.34760
C	17.45100	-3.99790	33.74650	H	9.23470	-1.55210	40.53700
C	16.68290	-3.91050	32.58450	H	6.27420	-1.63160	40.90530

H	8.01330	-0.69210	38.59570
H	7.25430	0.83700	40.48010
H	5.59750	0.58350	39.97310
H	5.93610	-0.51400	37.16710
H	6.36210	-2.18370	37.51810
H	5.03640	-1.41940	38.40200
H	6.54770	2.73730	39.07380
H	7.80150	1.82770	38.22070
H	6.10590	1.66310	37.74380
H	9.09300	-3.17320	39.25590
H	7.30680	-5.25110	38.18360
H	9.45840	-6.23940	37.33950
H	10.37680	-5.00190	38.22360
H	11.97150	2.44260	41.90670
H	11.78160	-0.01190	40.36250
H	9.62510	1.17790	40.13500
H	10.52220	-0.22800	38.57290
H	9.47160	3.17180	38.71690
H	10.49280	3.56060	40.11490
H	11.23530	3.25500	38.53920
H	13.41650	-0.09420	38.86880
H	15.44740	0.70270	37.79900
H	15.13940	2.31320	38.47190
H	12.16620	1.24430	37.15250
H	12.53010	1.54800	34.33830
H	10.23980	2.67960	34.44660
H	10.32330	2.47760	36.19330
H	10.85780	-0.09030	35.58000
H	10.20840	0.33320	34.00400
H	8.08010	1.16480	35.22130
H	8.80910	0.58020	36.70830
H	8.66480	-1.67740	35.95000
H	7.03080	-0.81300	32.44800
H	7.64570	0.50620	33.29230
H	6.81750	-2.86920	33.30690
H	7.64500	-3.31320	34.77810
H	8.49460	2.91900	28.61770
H	6.66080	1.33920	30.25810
H	8.65200	0.17870	31.12780
H	10.38110	1.84140	30.79480
H	10.21880	1.75550	29.04170
H	8.96690	-1.30940	29.23900
H	7.29120	-0.76410	29.22940
H	8.45090	-0.06790	28.08310
H	12.20630	0.49460	29.99800
H	11.08030	-0.62940	30.77130
H	11.14850	-0.52830	29.00500
H	8.80040	-5.73850	28.82600
H	6.65790	-5.95280	30.80180
H	8.28630	-3.49270	29.94200
H	6.95490	-3.47090	31.06150
H	8.25890	-4.92390	32.66720
O	9.38780	-2.07560	31.84870
N	10.30720	-3.45080	33.41820
H	10.20810	-4.31660	33.92540
H	10.85610	-2.72620	33.86940
H	17.51880	-6.65420	39.73010
H	16.72090	-9.28890	40.69250
H	14.82700	-7.72400	40.49810
H	14.70420	-9.02480	39.33670
H	15.56690	-5.14740	39.43640
H	15.54420	-4.19630	37.11390
H	14.63140	-9.52200	36.85550
H	15.05230	-5.14220	34.45900
H	14.28240	-9.37500	34.40240
H	14.49260	-7.20230	33.22380
H	21.08780	-7.50520	31.53310
H	22.54800	-5.72800	32.39960
H	21.35590	-3.86640	31.37920
H	21.46590	-3.66360	33.12460
H	19.19580	-4.00310	30.28790
H	19.41020	-4.19730	34.57250
H	16.72480	-3.84700	30.43040
H	16.98820	-3.95220	34.72590
H	15.60200	-3.82790	32.64830
H	21.80110	-2.64170	35.70910
H	20.41850	-0.14790	35.05940
H	20.06950	0.10970	37.56860
H	20.61150	-1.55020	37.77430
H	18.38190	2.40330	46.19720
H	16.97560	4.53460	44.78860
H	14.89560	3.33610	45.51390
H	15.87170	2.06230	46.22160
H	16.85790	1.53960	43.95920

H	15.88000	2.82300	43.24200
H	13.79620	1.56520	43.91300
H	14.81440	0.28820	44.60130
H	15.77720	-0.19840	42.37980
H	14.87760	1.15960	41.65790
H	12.82850	0.10960	42.27160
H	13.67530	-0.90090	41.22270
H	13.64530	-1.30170	42.78200
H	10.68880	-7.58810	43.98000
H	12.08050	-9.88320	45.10450
H	11.62180	-10.33720	42.77230
H	13.32020	-9.92770	42.93140
H	16.70810	-4.45310	42.88650
H	19.17790	-2.91230	43.05930
H	18.38070	-2.66080	40.80830
H	17.92910	-1.20730	41.68470
H	15.00730	-2.26180	39.60790
H	16.70950	-1.98250	39.25280
H	12.87510	-6.56220	38.83480
H	12.22030	-7.75000	39.67170
H	13.62430	-4.12570	41.51880
H	13.70420	-5.43540	42.36010
C	15.25150	2.07020	31.28370
C	14.31760	1.19910	32.10390
O	13.10110	1.38020	32.12470
N	14.93170	0.20370	32.82000
C	14.25230	-0.40870	33.96000
H	13.79830	0.36120	34.57450
C	15.30520	-1.10070	34.82010
H	15.77540	-1.91330	34.24980
O	16.26070	-0.13290	35.17380
C	14.61370	-1.71950	36.03290
H	14.12710	-0.93020	36.61920
O	15.52340	-2.45690	36.83420
C	13.53110	-2.68280	35.53160
H	14.01420	-3.47730	34.95350
C	12.75390	-3.34370	36.64420
O	11.93840	-2.36610	37.35170
P	11.89890	-2.53270	39.16310
O	13.17690	-1.76130	39.50490
O	10.55010	-1.80240	39.30280
O	11.87750	-4.04020	39.37340
O	12.58900	-1.99210	34.68610
C	13.14460	-1.38010	33.53820
H	12.31110	-0.83970	33.07860
O	13.63000	-2.37590	32.65500
H	15.64300	2.86460	31.93000
H	14.68950	2.52890	30.46950
H	16.09930	1.50710	30.88320
H	15.91830	0.34660	33.02120
H	17.11880	-0.62420	35.28440
H	16.36810	-1.95990	37.02070
H	12.08750	-4.11030	36.23990
H	13.42700	-3.82310	37.34950
H	14.34070	-1.95800	32.14570
H	10.34180	-0.03210	44.63210
H	7.35740	-6.40860	40.28370
H	10.76350	1.34900	42.25250
H	13.18650	3.72380	33.97200
H	7.22390	2.03090	28.12550
H	7.75640	3.81510	31.07240
H	8.68680	-6.77460	30.08430
H	5.85660	-5.86730	28.33790
H	18.45720	-8.85980	38.50080
H	19.93410	-6.41100	31.27750
H	21.40760	-5.70480	34.67180
H	20.33090	-2.48060	34.96310
H	22.18110	1.01860	36.31070
H	18.60450	2.91590	44.67180
H	17.81710	5.16340	47.15860
H	10.01250	-8.96010	44.55950
H	12.92140	-7.60130	45.89840
H	18.21840	-4.95170	42.46670
H	17.45650	-2.80050	45.07170
H	17.23360	-7.09690	41.26820
H	10.84230	-2.68850	37.04470

M1-GlcNAc6P-P

N	9.83080	-0.31240	44.76220
C	10.02440	-1.15160	43.58730
C	8.63750	-1.37550	42.94600
O	7.63510	-1.45610	43.65430
C	10.67040	-2.53740	43.88180
C	11.09770	-3.25730	42.61960

O	10.79200	-4.43290	42.40030
O	11.82640	-2.49810	41.82940
N	8.63960	-1.49290	41.59870
C	7.42670	-1.77690	40.83610
C	7.39780	-3.23850	40.36550
O	6.51880	-4.02520	40.72420
C	7.28820	-0.77540	39.65700
C	7.04490	0.64110	40.21210
C	6.19000	-1.20870	38.67520
C	7.08180	1.74060	39.14580
N	8.35470	-3.61280	39.48290
C	8.20530	-4.91880	38.85900
C	8.31700	-6.00310	39.91910
O	9.33890	-6.18710	40.56650
C	9.25250	-5.10630	37.74250
C	8.89050	-4.11250	36.63620
O	9.38880	-2.94140	36.72270
O	8.06580	-4.48810	35.76190
N	11.98710	1.51260	41.63410
C	12.03230	1.03490	40.24230
C	13.32670	1.53000	39.60150
O	13.79230	2.63530	39.89880
C	10.80240	1.41280	39.36760
O	10.77500	0.58160	38.20900
C	10.75780	2.86910	38.92700
N	13.87270	0.69750	38.68140
C	14.85640	1.17510	37.72150
C	14.23100	1.75960	36.44820
O	14.89270	2.44570	35.67240
N	12.91940	1.48340	36.21640
C	12.30640	2.08930	35.04050
C	12.69690	3.55930	34.95370
O	12.47010	4.35630	35.83960
C	10.77920	1.96130	35.08890
C	10.27140	0.51680	34.95550
C	8.86340	0.41620	35.55640
N	8.24500	-0.88920	35.35160
C	7.47590	-1.20950	34.32250
N	7.08670	-0.28880	33.40410
N	6.97380	-2.45500	34.24920
N	7.12740	2.48430	28.98740
C	7.38240	1.74440	30.22670
C	7.80050	2.73090	31.30400
O	8.06540	2.39980	32.44600
C	8.34220	0.51620	30.14230
C	9.75530	0.91780	29.67310
C	7.71540	-0.57810	29.27280
C	10.70160	-0.26980	29.47940
N	7.77660	-6.54880	29.84440
C	6.97380	-5.37830	30.21090
C	6.11480	-5.01480	29.00300
O	5.79480	-3.88530	28.70220
C	7.73210	-4.15970	30.76110
C	8.50400	-4.48050	32.03450
C	9.36240	-3.31830	32.55110
H	9.18170	-5.32510	31.86000
N	17.68110	-7.28850	40.20550
C	16.78520	-8.39550	39.84560
C	17.41730	-9.20110	38.70550
O	16.86460	-10.13010	38.16000
C	15.32420	-7.98380	39.50100
C	15.26730	-7.07940	38.30460
C	15.53400	-5.72290	38.28290
C	15.07420	-7.46690	36.92470
N	15.55130	-5.25580	36.98700
C	15.23090	-6.29300	36.13470
C	14.76310	-8.68470	36.29170
C	15.05040	-6.31010	34.74490
C	14.59010	-8.70070	34.91370
C	14.72670	-7.52320	34.14990
N	21.15900	-6.62320	31.36490
C	21.56460	-5.76180	32.46590
C	21.10250	-6.32660	33.79690
O	20.38900	-7.29730	33.92210
C	21.10150	-4.27730	32.35100
C	19.59800	-4.13740	32.36400
C	18.86390	-4.03300	31.17740
C	18.89650	-4.16410	33.57590
C	17.47000	-3.96570	31.20300
C	17.50980	-4.07410	33.61050
C	16.78810	-3.98700	32.41920
N	21.18910	-2.02160	35.08140
C	20.97020	-0.71350	35.68680
C	22.30140	-0.04920	36.00770

O	23.37080	-0.61950	36.03850	H	6.10140	-0.49580	37.85090
C	20.02270	-0.72870	36.92880	H	6.41220	-2.18220	38.23050
C	18.57360	-1.17280	36.56640	H	5.21780	-1.27570	39.17830
O	17.74380	-1.34560	37.51120	H	7.06090	2.73520	39.60350
O	18.32090	-1.29840	35.33710	H	7.99330	1.66850	38.54390
N	18.46720	3.40890	45.54860	H	6.22970	1.67700	38.46190
C	17.07540	3.87270	45.57240	H	9.14500	-3.01130	39.24590
C	16.86980	4.65120	46.87840	H	7.19900	-4.98840	38.42800
O	15.84180	4.66800	47.51790	H	9.19100	-6.12560	37.35410
C	15.99000	2.80580	45.33750	H	10.25130	-4.92790	38.14720
C	16.09100	2.12750	43.96200	H	12.36660	2.45940	41.65540
C	14.99270	1.07880	43.73850	H	12.03000	-0.04780	40.25980
C	15.07860	0.43360	42.35330	H	9.92050	1.19200	39.99110
N	13.91900	-0.49000	42.14670	H	10.76030	-0.35150	38.52610
N	10.82230	-8.32250	44.73470	H	9.80590	3.08400	38.43430
C	12.06980	-9.03540	44.43900	H	10.85320	3.53130	39.79300
C	13.18210	-8.12920	44.95310	H	11.56430	3.10820	38.22850
O	14.08010	-7.66360	44.27840	H	13.50990	-0.25330	38.62120
C	12.28520	-9.51940	42.99950	H	15.54260	0.37400	37.43350
C	12.17490	-8.48920	41.86790	H	15.44120	1.97420	38.17970
O	11.70990	-7.32530	42.14250	H	12.32550	1.05740	36.92560
O	12.52660	-8.89030	40.73540	H	12.68040	1.61290	34.13340
N	17.76120	-4.42630	43.26690	H	10.34130	2.56600	34.28550
C	18.10660	-3.03260	43.00880	H	10.44220	2.40390	36.03290
C	17.58420	-2.17230	44.16470	H	10.92200	-0.19160	35.48100
O	17.35970	-0.98100	44.09260	H	10.26310	0.21430	33.90140
C	17.76250	-2.46150	41.61760	H	8.20810	1.19980	35.16050
C	16.30440	-2.67460	41.19720	H	8.93670	0.57390	36.63510
O	15.44490	-3.04860	42.03070	H	8.63550	-1.68520	35.90510
N	16.02300	-2.41810	39.91470	H	6.71320	-0.66330	32.54490
Mg	11.55540	-5.69800	40.89300	H	7.64000	0.55140	33.25720
O	12.08990	-6.83100	39.25630	H	6.59220	-2.74630	33.36300
O	13.58170	-5.35980	41.66360	H	7.38430	-3.20810	34.84860
H	8.95000	-0.58870	45.19430	H	8.00850	2.72130	28.53760
H	10.67730	-0.63560	42.87580	H	6.40730	1.37040	30.57540
H	9.99070	-3.17560	44.45070	H	8.45160	0.10810	31.15210
H	11.57510	-2.37360	44.48340	H	10.19940	1.59120	30.41480
H	9.51380	-1.46670	41.06050	H	9.68940	1.47780	28.72750
H	6.58270	-1.67810	41.52150	H	8.29370	-1.49700	29.34980
H	8.24560	-0.77140	39.11660	H	6.69220	-0.81560	29.58770
H	7.80270	0.85120	40.97630	H	7.67780	-0.28450	28.21630
H	6.07810	0.65900	40.73450	H	11.72010	0.08550	29.31020

H	10.70990	-0.90240	30.37010	H	16.00040	-0.13610	42.24700
H	10.40840	-0.88270	28.62150	H	15.02280	1.18880	41.56600
H	8.58700	-6.25730	29.30160	H	13.05140	0.09710	42.23930
H	6.24990	-5.72360	30.96870	H	13.85700	-0.96170	41.20580
H	8.42530	-3.79040	29.99760	H	13.92320	-1.26780	42.80740
H	7.02470	-3.33950	30.92470	H	10.69520	-7.62540	44.00070
H	7.83240	-4.80290	32.84250	H	12.10240	-9.91570	45.10210
O	9.41250	-2.22300	31.97750	H	11.54770	-10.30010	42.77460
N	10.05400	-3.63240	33.65870	H	13.26690	-9.99400	42.89970
H	9.78990	-4.45030	34.18720	H	16.78260	-4.57120	43.03180
H	10.68000	-2.98030	34.12290	H	19.20030	-2.94640	43.12300
H	17.63260	-6.59000	39.46250	H	18.41640	-2.90800	40.85990
H	16.74700	-9.08250	40.70350	H	17.96920	-1.38430	41.60390
H	14.89230	-7.48750	40.37830	H	15.03750	-2.42670	39.62690
H	14.72860	-8.88630	39.33770	H	16.72470	-2.08160	39.25540
H	15.68810	-5.03740	39.10660	H	12.88270	-6.51520	38.79670
H	15.59240	-4.25740	36.75670	H	12.31400	-7.72220	39.67350
H	14.67800	-9.59430	36.87560	H	14.08800	-4.52810	41.72970
H	15.15680	-5.41380	34.14950	H	13.76580	-5.90530	42.44250
H	14.34960	-9.63610	34.41180	C	14.55330	1.59660	30.85130
H	14.58310	-7.56220	33.07270	C	13.74790	0.72660	31.80180
H	20.44680	-7.26550	31.71030	O	12.52810	0.83640	31.91640
H	22.66760	-5.72900	32.54120	N	14.48270	-0.17470	32.52690
H	21.51410	-3.87890	31.41570	C	13.91410	-0.79830	33.71900
H	21.54320	-3.68260	33.15940	H	13.37300	-0.05960	34.30670
H	19.38980	-3.98890	30.22550	C	15.05640	-1.34990	34.57480
H	19.43820	-4.25870	34.51120	H	15.55300	-2.16870	34.03850
H	16.91610	-3.89150	30.27040	O	15.95790	-0.29510	34.81170
H	17.00540	-4.05540	34.57000	C	14.45250	-1.91150	35.86080
H	15.70430	-3.92850	32.44410	H	13.94700	-1.09720	36.39250
H	21.67340	-2.62410	35.74350	O	15.43280	-2.52290	36.69210
H	20.47320	-0.08030	34.93830	C	13.39490	-2.97130	35.51620
H	19.94260	0.26770	37.38630	H	13.87980	-3.82860	35.03790
H	20.42140	-1.39510	37.70460	C	12.62180	-3.44410	36.73740
H	18.55590	2.56460	46.11160	O	11.93840	-2.36610	37.35170
H	16.99100	4.64100	44.78470	P	11.90980	-2.59310	40.00890
H	15.00720	3.27530	45.46280	O	13.26330	-1.89550	39.86010
H	16.06750	2.04880	46.13070	O	10.65600	-1.76730	39.69750
H	17.06530	1.63550	43.85930	O	11.83190	-4.08240	39.72490
H	16.02880	2.89040	43.17270	O	12.41780	-2.42000	34.60760
H	14.00580	1.54670	43.85230	C	12.89770	-1.89360	33.39680
H	15.08140	0.29740	44.50380	H	12.01680	-1.47520	32.89750

O	13.46410	-2.92750	32.60140
H	14.90190	2.48270	31.39450
H	13.90790	1.92360	30.03470
H	15.42770	1.07620	30.45020
H	15.46840	0.04090	32.65210
H	16.85710	-0.68700	34.97910
H	16.24230	-1.95940	36.83660
H	11.92080	-4.23130	36.43130
H	13.31660	-3.88420	37.45710
H	14.04490	-2.47620	31.96930
H	10.57580	-0.45600	45.43790
H	7.44340	-6.65810	40.07510
H	11.03170	1.54040	41.98280
H	13.11800	3.88320	33.97800
H	6.62810	1.89140	28.33250
H	7.82800	3.79700	30.99810
H	8.13470	-7.01300	30.67320
H	5.79510	-5.89650	28.40480
H	18.44770	-8.88730	38.42520
H	20.72110	-6.07240	30.63230
H	21.48250	-5.77320	34.68490
H	20.26760	-2.40610	34.89730
H	22.22410	1.03620	36.25830
H	18.73580	3.14990	44.60410
H	17.77340	5.21840	47.19500
H	10.03680	-8.96530	44.67980
H	13.07880	-7.87220	46.03000
H	18.32160	-5.05120	42.69450
H	17.46120	-2.73920	45.11150
H	17.30150	-6.82700	41.03190
H	10.97800	-2.50420	37.19370
